# In-Hospital and One-Year Outcomes of Patients after Early and Late Resuscitated Cardiac Arrest Complicating Acute Myocardial Infarction—Data from a Nationwide Database

**DOI:** 10.3390/jcm11030609

**Published:** 2022-01-26

**Authors:** Robert Kowalik, Marek Gierlotka, Krzysztof Ozierański, Przemysław Trzeciak, Anna Fojt, Piotr Feusette, Agnieszka Tycińska, Grzegorz Opolski, Marcin Grabowski, Mariusz Gąsior

**Affiliations:** 1 First Department of Cardiology, Medical University of Warsaw, 02-097 Warsaw, Poland; rjkowalik@wp.pl (R.K.); anna.fojt@o2.pl (A.F.); grzegorz.opolski@wum.edu.pl (G.O.); marcin.grabowski@wum.edu.pl (M.G.); 2 Department of Cardiology, Institute of Medical Sciences, University of Opole, 45-401 Opole, Poland; marek.gierlotka@gmail.com (M.G.); feusette@wp.pl (P.F.); 3 3rd Department of Cardiology, Faculty of Medical Sciences in Zabrze, Silesian Centre for Heart Diseases in Zabrze, Medical University of Silesia in Katowice, 41-800 Zabrze, Poland; przemyslaw.t@wp.pl (P.T.); m.gasior@op.pl (M.G.); 4 Department of Cardiology, Medical University of Bialystok, 15-276 Bialystok, Poland; agnieszka.tycinska@gmail.com

**Keywords:** acute coronary syndrome, cardiac rehabilitation, sudden cardiac death, life-threatening ventricular arrhythmia, early and late cardiac arrest, secondary prevention of sudden cardiac death

## Abstract

The prognostic role of early (less than 48 h) resuscitated cardiac arrest (ErCA) complicating acute myocardial infarction (AMI) is still controversial. The present study aimed to analyse the short-term and one-year outcomes of patients after ErCA and late resuscitated cardiac arrest (LrCA) compared to patients without cardiac arrest (CA) complicating AMI. Data from the prospective nationwide Polish Registry of Acute Coronary Syndromes (PL-ACS) were used to assess patients with resuscitated cardiac arrest (rCA) after AMI. Baseline clinical characteristics and the predictors of all-cause death were assessed. The all-cause mortality rate, complications, performed procedures, and re-hospitalisations were assessed for the in-hospital period, 30 days after discharge, and 6- and 12-month follow-ups. Among 167,621 cases of AMI, CA occurred in 3564 (2.1%) patients, that is, 3100 (87%) and 464 (13%) patients with ErCA and LrCA, respectively. The mortality rates in the ErCA vs. LrCA and CA vs. non-CA groups were as follows: in-hospital: 32.1% vs. 59.1% (*p* < 0.0001) and 35.6% vs. 6.0% (*p* < 0.0001); 30-day: 2.2% vs. 3.2% (*p* = 0.42) and 9.9% vs. 5.2% (*p* < 0.0001); 6-month: 9.2% vs. 17.9% (*p* = 0.0001) and 12.3% vs. 21.1% (*p* < 0.0001); and 12-month: 12.3% vs. 21.1% (*p* = 0.001) and 13% vs. 7.7% (*p* < 0.0001), respectively. ErCA (hazard ratio (HR): 1.54, confidence interval (CI):1.28–1.89; *p* < 0.0001) and LrCA (HR: 2.34, CI: 1.39–3.93; *p* = 0.001) increased the risk of 12-month mortality. During the 12-month follow-up, patients after LrCA more frequently required hospitalisation due to heart failure compared to patients after ErCA. ErCA was related to a higher hospitalisation rate due to coronary-related causes and a higher rate of percutaneous coronary intervention. An episode of LrCA was associated with higher in-hospital and long-term mortality compared to ErCA. ErCA and LrCA were independent risk factors for one-year mortality.

## 1. Introduction

In recent decades, the incidence of cardiac arrest (CA) prior to hospital admission has decreased due to the fast reperfusion strategy and pharmacotherapy [1,2]. Despite this, acute myocardial infarction (AMI) is still the leading cause of life-threatening ventricular arrhythmias and CA [1,3,4]. Approximately 6–8% of patients develop hemodynamically unstable sustained ventricular tachycardia (VT) or ventricular fibrillation (VF) during the acute phase of AMI [1]. The predictors of CA differ regarding the time after AMI onset [5]. The in-hospital mortality rate of AMI is 10.5%, and it depends mainly on age and baseline clinical presentation (e.g., signs of heart failure, completeness of coronary revascularisation, and the occurrence of CA) [6]. CA requires an early introduction of guideline-recommended therapy and qualification for an implantable cardioverter–defibrillator (ICD) for the secondary prevention of sudden cardiac death (SCD), usually in patients presenting with CA occurring 48 h after AMI onset [1]. The available data suggest that patients after resuscitated CA (rCA) during hospitalisation for AMI are at a higher risk of in-hospital and post-discharge adverse events [7]. However, the prognostic role of early rCA (ErCA) (less than 48 h from the onset of AMI), particularly in long-term observations, is still controversial, and new biomarkers could improve risk stratification [8]. It is postulated that early CA is related to possible reversible causes of CA, such as acute ischemia, acute heart failure, and/or electrolyte disturbances, inducing malignant ventricular arrhythmia, and it has no impact on long-term prognosis, similar to patients without a CA episode [9].

Further data on the significance of ErCA are warranted for better early and long-term risk stratification. The present study aimed to compare the short-term and 12-month outcomes of patients after ErCA and late rCA (LrCA) to patients without CA complicating AMI.

## 2. Methods

### 2.1. Study Design

Data from the prospective nationwide Polish Registry of Acute Coronary Syndromes (PL-ACS) from 2005 to 2014 were used for the analysis. The registry was designed to gather comprehensive data on the management and long-term outcomes of AMI patients in Poland. A detailed description of the database and the methods used have been previously published [10]. In brief, the ongoing registry is linked to the government and National Health Fund data, which is the only public healthcare insurer in Poland. Data were collected using questionnaires, including demographics, medical history, clinical condition on admission, type of AMI, related procedures, and treatment. The all-cause mortality rate was assessed for the in-hospital period, 30 days after discharge, and 6-month and 12-month follow-ups. At the one-year follow-up, data on re-hospitalisations (e.g., heart failure, recurrent AMI or CA, stroke), procedures (e.g., coronary angiography, percutaneous coronary intervention (PCI), coronary artery bypass grafting (CABG), ablation, cardiac device implantation), and cardiac rehabilitation were gathered. For one-year outcome analyses, only patients with CA after AMI, who survived the index hospitalisation, were included. All analyses were performed for patients with CA (ErCA and LrCA), as well as for patients without CA complicating AMI.

The present study is a retrospective analysis of the prospective Polish Registry of Acute Coronary Syndromes (PL-ACS), which is a nationwide official government registry within the Polish health system: https://isap.sejm.gov.pl/isap.nsf/DocDetails.xsp?id=WDU20180001063 (accessed on 2 December 2021). Therefore, official approval of the study was not required from the Ethics Committee.

### 2.2. Definitions

ErCA patients were defined as those with CA within the first 48 h of AMI onset, regardless of whether the CA appeared during the pre-hospital or in-hospital phase. LrCA patients were defined as those with CA after the first 48 h of the onset of AMI.

### 2.3. Statistical Analysis

Continuous variables were presented as means (SD) or median values and interquartile ranges (IQRs) and compared between the groups by means of a Student’s *t*-test or Mann–Whitney U test according to data distribution, respectively. Categorical data were presented as numbers of patients and percentages and compared with the use of the χ^2^ test with Pearson modification. Logistic regression was used to define predictors of in-hospital mortality and rCA occurrence during AMI among survivors. 

The associations between the analysed groups and follow-up outcomes were analysed using the unadjusted and adjusted Kaplan–Meier method for multiple group comparisons. Adjusted survival and hazard ratios were calculated using the inverse probability method. A *p* value of less than 0.05 was considered statistically significant. All reported *p* values are two-sided. Analyses were performed with the use of Statistica version 13 (TIBCO Software Inc., Palo Alto, CA, USA (2017)) and R version 4.0.3 (R Foundation for Statistical Computing, Vienna, Austria).

## 3. Results

### 3.1. Study Population

The study included 167,621 cases of AMI from the 2009–2014 period. CA occurred in 3564 (2.1%) patients, that is, 3100 (87%) and 464 (13%) patients with ErCA and LrCA, respectively. Compared to the non-CA group, patients with an rCA episode were younger, more predominantly male, smokers, and more likely to have a diagnosis of STEMI, and they had more intra-hospital complications. In addition, compared to the non-CA group, patients after rCA were more likely to have a history of stroke, ischemic heart disease, and heart failure chronic obstructive pulmonary disease. Patients with rCA less frequently had a history of hypertension, hypercholesterolemia, and a previous AMI compared to the non-CA group (Table S1).

From the ErCA group and the LrCA group, 2106 patients and 190 patients, respectively, survived until discharge from the hospital. There was no difference between the groups regarding gender. The LrCA patients were older (67.9 (±12.3) vs. 63.1 (±11.5) years old (*p* < 0.001)), had a lower left ventricular ejection fraction (38 (±13) vs. 44 (±12)%), and a longer hospital stay, and they more often required implantation of a pacemaker or ICD during the index hospitalisation compared to the ErCA patients. In the ErCA group, myocardial infarction with ST-segment elevation was more frequent, while in the LrCA group, myocardial infarction without ST-segment elevation was more frequent. The ErCA patients were more likely to have Killip–Kimball class 4 during hospitalisation, as well as coronary angiography. Additionally, the LrCA group had a pulmonary oedema, recurrent AMI, and massive bleeding with blood transfusion episodes more often during the index hospitalisation. Both groups received optimal guideline-recommended therapy. There was no difference in pharmacotherapy at discharge, except for diuretics, vitamin K antagonists, and antidiabetic medications, which were more commonly prescribed in the LrCA group. The detailed clinical characteristics of both groups are presented in Table 1, Table 2, Table 3 and Table 4.

### 3.2. In-Hospital and One-Year Observation Results

The overall mortality in AMI patients was higher in the CA group than in patients without CA: 35.6% and 6.0% in hospital, 2.3% and 1.1% at 30 days, 9.9% and 5.2% at 6 months, and 13% and 7.7% at 12 months (all *p* < 0.0001), respectively. There was no statistically significant difference in 30-day mortality between the ErCA and LrCA groups (2.2% vs. 3.2%; *p* = 0.42). At the 6- and 12-month follow-up periods, all-cause mortality was higher in the LrCA patients than in the ErCA patients (17.9% vs. 9.2%; *p* = 0.0001 and 21.1% vs. 12.3%; *p* = 0.001, respectively). Detailed data on the one-year outcomes are presented in Table 5 and Supplementary Materials.

**Figure 1:**
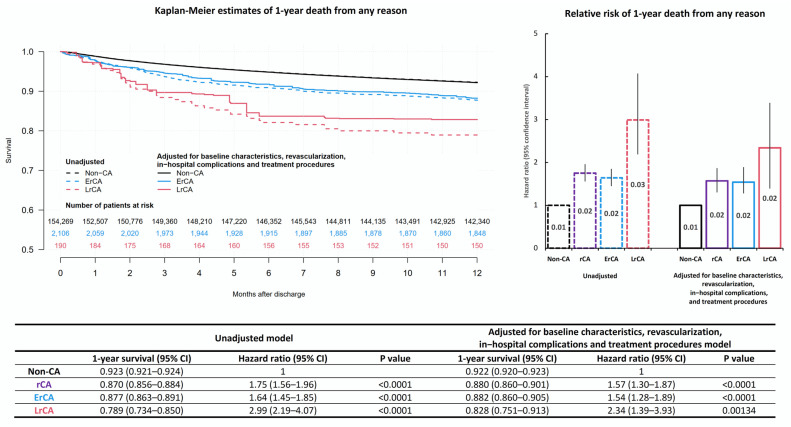
Central illustration. Risk of death from any reason for patients discharged home after resuscitated cardiac arrest during acute myocardial infarction.

In a multivariable model adjusted for all relevant risk factors, both ErCA (HR 1.54; 1.28–1.89; *p* < 0001) and LrCA (HR 2.34; CI 1.39–3.93; *p* = 0.001) increased the risk of one-year death compared to patients without CA after AMI (Figure 1).

In the multivariable analysis, the occurrence of an LrCA episode was also the second strongest factor (after Killip class 4) associated with an over 10-fold increase in the risk of in-hospital death (HR 10.30; CI 8.02–13.20; *p* < 0.0001), whereas ErCA increased the risk almost 2.5-fold (HR 2.35; CI 2.1–2.63; *p* < 0.0001) (Figure S1). The factors associated with an increased risk of CA occurrence after AMI are presented in Figure S2. 

During the one-year observation, the ErCA patients were more frequently hospitalised due to chronic coronary syndrome, coronary angiography, and PCI when compared to the LrCA patients. The latter group was more likely to be hospitalised due to heart failure (17.9% vs. 10.8%; *p* = 0.003). Interestingly, only 17.9% of patients in the LrCA group underwent cardiac rehabilitation compared to 27.5% (*p* = 0.004) of the ErCA group. Figure S3 presents 1-year survival, re-hospitalisations, and procedures in AMI patients discharged home who survived cardiac arrest (early or late) compared to patients without cardiac arrest.

**Figure 2:**
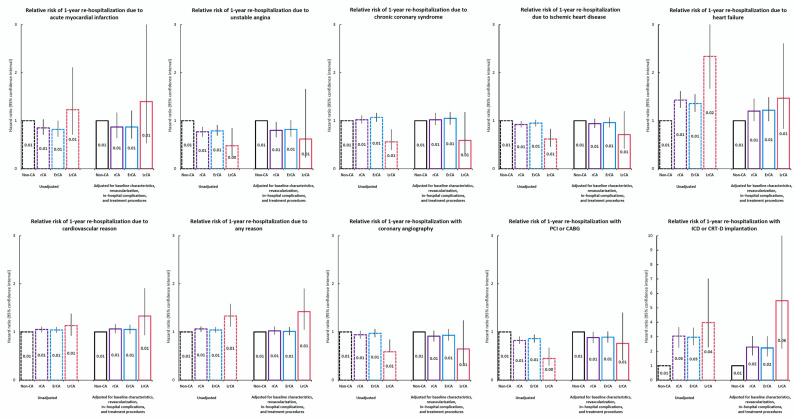
Relative risk of 1-year re-hospitalisation.

A comparison of the calculated relative risk of different re-hospitalisations associated with the type of CA is presented in Figure 2.

## 4. Discussion

The main observation of the present study is that ErCA, as a complication of AMI, is an independent prognostic factor of one-year mortality. ErCA, after adjustment of many factors influencing statistical significance, increases the risk of death in the 12-month follow-up (adjusted HR = 1.5).

The available data do not show a clear assessment of the impact of ErCA on the long-term prognosis of patients after AMI. Our study shows the influence of ErCA on mortality, which may be important for future recommendations for the secondary prevention of cardiac arrest.

In one of the thrombolysis-era studies based on data from the GISSI-2 database, both early and late CA episodes increased in-hospital mortality (2.5 times and 4 times, respectively), while they had no significant effect on mortality in the 6-month follow-up [11]. The results from one of the prospective cohort studies by Bougouin et al. showed that patients with AMI complicated by early ventricular fibrillation had a similar number of sudden cardiac death events during the 5-year follow-up to the group without ventricular fibrillation [12].

After an ErCA episode, which occurred within an established time of less than 48 h from the onset of AMI, the patients were classified as a non-homogeneous group with a different prognosis. This group included both patients who survived out-of-hospital CA (OHCA) and in-hospital CA (IHCA) prior to coronary reperfusion and those who had an arrhythmia during or after reperfusion. OHCA patients had by far the worst prognosis. It was demonstrated by Nair et al. that only 4% of patients from this group survived until discharge from the hospital [13].

A recent study by Podolecki et al. showed that the risk of death in long-term observation was associated with the occurrence of life-threatening ventricular arrhythmia only in the pre-reperfusion period [14]. However, this effect on long-term prognosis lost its significance after excluding patients who died within the first 30 days from admission. A similar effect of pre-reperfusion CA on long-term prognosis has also been shown in two other studies by Piccini et al. and Liang et al. [15,16]. Moreover, all three cited papers showed a significantly increased effect of early ventricular arrhythmias on in-hospital mortality [14,15,16].

Interestingly, the assessment of the effect of ventricular arrhythmia during revascularisation procedures gives conflicting results in terms of short-term prognosis, but most of the studies did not show an increase in mortality in the long-term follow-up [17].

Based on the current analysis, mainly LrCA, but also ErCA, is one of the strongest predictors of in-hospital and one-year mortality. LrCA increased the risk of in-hospital death by more than 10-fold (4-fold more than in the ErCA group). This observation is in line with the results of previously published papers [14,18], which showed that the occurrence of late ventricular arrhythmia is associated with a higher risk of in-hospital death by over 8-fold. In terms of the long-term prognosis, the occurrence of late ventricular arrhythmia increased the risk of death in long-term observation by nearly 3.5 times [14]. Another study by Mehta et al. showed that at any stage of follow-up, mortality in the LrCA group remained at a higher level than in the ErCA group [19]. This last observation is in line with our results. There were no differences in mortality in the 30-day observation between the ErCA and LrCA groups in our study, but the mortality was significantly higher in the LrCA group at 6- and 12-months post-hospital discharge. 

We did not analyse the rhythm presentation of CA, but we collected data about the ICD and CRT-D implantation rate. In general, VF or VT is a main mechanism of CA in MI (about 80%) [9]. In our study, only 14.2% patients after LrCA, who survived until discharge from the hospital, had an implanted ICD or CRT-D, which suggests a high percentage of non-defibrillated rhythms of CA, probably due to the high percentage of severe in-hospital complications.

The next important finding from our study is the fact that ErCA episodes occur more often in younger patients. This observation was also confirmed in the study by Sulzgruber et al. [20]. This study found an inverse correlation of patient age with the risk of developing ventricular arrhythmia in the acute phase of AMI. In addition, in this study, and in that of Piccini et al., diabetes and hypertension were found to be paradoxically protective against ventricular arrhythmias in the ErCA group [15]. This observation also coincides with the results of the present research. It could be assumed that the pharmacological treatment (beta-blockers, angiotensin-converting enzyme inhibitors, and statins) frequently used in these diseases might be responsible for such an effect.

Another important observation comes from the 12-month follow-up. The patients after ErCA were more often admitted to the hospital due to coronary events (chronic coronary syndromes, unstable angina, or recurrent AMI). They were also referred to coronary angiography and PCI more frequently than patients after LrCA. These results emphasise the importance of optimising medical care in this group. 

Finally, it is worth emphasising the importance of early revascularisation strategies, which were significantly less frequently used in the LrCA group. Consequently, a significant increase in the number of repeated AMI and more frequent cardiogenic shock were observed in this study. Perhaps this observation partially explains the problem of such highly unfavourable short- and long-term prognoses of patients in older age groups treated for AMI complicated by CA.

## 5. Limitations

The results of the present study need to be interpreted considering some limitations. This was a retrospective analysis of an observational registry, which may result in a selection error. The ErCA group was analysed as a homogenous group without consideration of the diversity of this population, thus creating significant differences in prognosis. In the registry, we did not collect data on the rhythm presentation of CA and the timing to the return of spontaneous circulation, which are important in terms of predicting recovery from CA. To reduce the important influences of these factors, we only analysed patients who recovered after CA (resuscitated CA), and we focused on the long-term outcomes. There was a lack of data on treatment methods and the degree of neurological dysfunction of the out-of-hospital CA patients.

## 6. Conclusions

Both ErCA and LrCA were independent risk factors for one-year mortality. An episode of LrCA was associated with higher in-hospital and one-year mortality than ErCA. The ErCA patients were younger and had less comorbidities than LrCA patients. Further well-designed studies are required to identify clinical outcomes after ErCA (OHCA and IHCA) in long term follow-ups.

## Figures and Tables

**Table 1:** Baseline clinical characteristics of patients without cardiac arrest and with ErCA and LrCA after acute myocardial infarction (only patients who were discharged after index hospitalisation).

Variable	Non-CA *N* = 154,266	rCA *N* = 2296	*p* Value	ErCA *N* = 2106	LrCA *N* = 190	*p* Value
Age, years, mean (SD)	65.8 (11.9)	63.5 (11.7)	<0.0001	63.1 (11.5)	67.9 (12.3)	<0.0001
Age ≥ 65 years	80,187 (52%)	990 (43.1%)	<0.0001	875 (41.6%)	115 (60.5%)	<0.0001
Age ≥ 75 years	41,892 (27.2%)	476 (20.7%)	<0.0001	413 (19.6%)	63 (33.2%)	<0.0001
Age ≥ 85 years	7548 (4.9%)	68 (3%)	<0.0001	52 (2.5%)	16 (8.4%)	<0.0001
Male gender	98,794 (64.1%)	1578 (68.9%)	<0.0001	1449 (68.9%)	129 (67.9%)	0.77
From home admission	88,130 (57.2%)	1461 (63.7%)	<0.0001	1344 (63.9%)	117 (61.6%)	0.53
Transfer from other hospital	66,041 (42.8%)	834 (36.3%)	<0.0001	761 (36.2%)	73 (38.4%)	0.53
Hipercholesterolemia	64,577 (41.9%)	879 (38.3%)	0.0006	798 (37.9%)	81 (42.6%)	0.2
Hypertension	112,427 (72.9%)	1440 (62.7%)	<0.0001	1311 (62.3%)	129 (67.9%)	0.12
Obesity	31,882 (20.7%)	445 (19.4%)	0.13	402 (19.1%)	43 (22.6%)	0.24
Diabetes mellitus	38,799 (25.9%)	474 (21.5%)	<0.0001	406 (20.1%)	68 (37%)	<0.0001
Smoking (current or past)	91,654 (59.4%)	1480 (64.5%)	<0.0001	1371 (65.1%)	109 (57.4%)	0.033
Current smoker	45,942 (29.8%)	800 (34.8%)	<0.0001	761 (36.1%)	39 (20.5%)	<0.0001
Ischemic heart disease diagnosed before AMI	21,800 (14.1%)	394 (17.2%)	<0.0001	347 (16.5%)	47 (24.7%)	0.004
Family history of CAD	17,452 (11.3%)	266 (11.6%)	0.68	239 (11.4%)	27 (14.2%)	0.24
History of heart failure	11,821 (7.7%)	215 (9.4%)	0.002	182 (8.6%)	33 (17.4%)	<0.0001
History of stroke	5689 (3.7%)	114 (5%)	0.001	104 (4.9%)	10 (5.3%)	0.84
Chronic kidney disease	9256 (6%)	149 (6.5%)	0.33	122 (5.8%)	27 (14.2%)	<0.0001
Peripheral artery disease	7026 (4.6%)	122 (5.3%)	0.084	111 (5.3%)	11 (5.8%)	0.76
Chronic obstructive pulmonary disease	6031 (3.9%)	120 (5.2%)	0.0013	107 (5.1%)	13 (6.8%)	0.3
Prior myocardial infarction	19,708 (12.8%)	259 (11.3%)	0.036	364 (17.3%)	39 (20.5%)	0.26
Prior PCI	27,128 (17.6%)	403 (17.6%)	0.97	232 (11%)	27 (14.2%)	0.18
Prior CABG	5090 (3.3%)	80 (3.5%)	0.62	72 (3.4%)	8 (4.2%)	0.57
NSTEMI	83,073 (53.9%)	721 (31.4%)	<0.0001	645 (30.6%)	76 (40%)	0.008
STEMI	71,194 (46.1%)	1575 (68.6%)	<0.0001	1461 (69.4%)	114 (60%)	0.008
CA before admission	0 (0%)	1169 (50.9%)	<0.0001	1160 (55.1%)	9 (4.7%)	<0.0001
ECG heart rate, bpm, mean (SD)	79.1 (24.3)	846 (27.7)	<0.0001	84.3 (27.7)	88 (28.1)	0.078
Sinus rhythm	140,391 (91.6%)	1918 (83.6%)	<0.0001	1775 (84.4%)	143 (75.3%)	0.0012
Atrial fibrillation	8692 (5.7%)	197 (8.6%)	<0.0001	167 (7.9%)	30 (15.8%)	0.0002
Other rhythm	2421 (1.6%)	147 (6.4%)	<0.0001	136 (6.5%)	11 (5.8%)	0.72
Normal QRS	127,755 (83.5%)	1765 (77.4%)	0.0012	1625 (77.7%)	140 (74.1%)	0.25
LBBB	5843 (3.8%)	117 (5.1%)	0.057	106 (5.1%)	11 (5.8%)	0.65
RBBB	5255 (3.4%)	95 (4.2%)	<0.0001	81 (3.9%)	14 (7.4%)	0.02
Other QRS abnormalities	14,184 (9.3%)	303 (13.3%)	<0.0001	279 (13.3%)	24 (12.7%)	0.8
Normal ST segment	12,279 (8%)	79 (3.4%)	<0.0001	66 (3.1%)	13 (6.8%)	0.007
ST-segment elevation	69,412 (45.2%)	1526 (66.5%)	<0.0001	1422 (67.6%)	104 (54.7%)	0.0003
ST-segment depression	36,818 (24%)	359 (15.6%)	<0.0001	320 (15.2%)	39 (20.5%)	0.053
Negative T waves	14,164 (9.2%)	87 (3.8%)	<0.0001	77 (3.7%)	10 (5.3%)	0.27
Other ST-segment abnormalities	19,835 (12.9%)	233 (10.2%)	<0.0001	209 (9.9%)	24 (12.6%)	0.24
Systolic BP, mmHg, median (IQR)	140 (120–155)	120 (100–140)	<0.0001	120 (100–140)	130 (110–142)	0.11
Killip class 1	12,1579 (79.3%)	1217 (53.1%)	<0.0001	1107 (52.6%)	110 (57.9%)	0.16
Killip class 2	20,415 (13.3%)	501 (21.8%)	<0.0001	451 (21.4%)	50 (26.3%)	0.12
Killip class 3	3728 (2.4%)	118 (5.1%)	<0.0001	106 (5%)	12 (6.3%)	0.45
Killip class 4	1988 (1.3%)	370 (16.1%)	<0.0001	361 (17.2%)	9 (4.7%)	<0.0001

Abbreviations: ACE-I—angiotensin-converting enzyme inhibitor; AMI—acute myocardial infarction; ARB—angiotensin receptor blocker; CA—cardiac arrest; CABG—coronary artery bypass grafting; CAD—coronary artery disease; ErCA—early resuscitated cardiac arrest; CRT-D—cardiac resynchronisation therapy defibrillator; CRT-P—cardiac resynchronisation therapy pacemaker; Cx—circumflex artery; IABP—intra-aortic balloon pump; ICD—implantable cardioverter–defibrillator; ECG—electrocardiogram; IQR—interquartile range; LAD—left anterior descending; LBBB—left bundle branch block; LM—left main; LMWH—low-molecular-weight heparin; LrCA—late resuscitated cardiac arrest; NSTEMI—nonST-segment myocardial infarction; PCI—percutaneous coronary intervention; RCA—right coronary artery; RBBB—right bundle branch block; rCA—resuscitated cardiac arrest; SD—standard deviation; STEMI—ST-segment myocardial infarction; TIMI—the thrombolysis in myocardial infarction risk score; VSD—ventricular septal defect.

**Table 2:** In-hospital treatment and procedures of patients without cardiac arrest and with ErCA and LrCA after acute myocardial infarction (only patients who were discharged after index hospitalisation).

Variable	Non-CA *N* = 154,266	rCA *N* = 2296	*p* Value	ErCA *N* = 2106	LrCA *N* = 190	*p* Value
Thrombolysis	521 (0.4%)	23 (11%)	<0.0001	20 (1%)	3 (1.7%)	0.39
Glycoprotein IIb/IIIa inhibitors	33,532 (21.7%)	881 (38.4%)	0.0032	814 (38.7%)	67 (35.3%)	0.36
Anticoagulation (not associated with PCI)	79,366 (51.8%)	1259 (54.9%)	0.013	1147 (54.5%)	112 (59%)	0.24
Coronary angiography	141,438 (91.7%)	2137 (93.2%)	0.7	1967 (93.5%)	170 (89.5%)	0.036
Infarct-related artery—RCA	42,924 (30.3%)	759 (35.5%)	<0.0001	708 (36%)	51 (30%)	0.2
Infarct-related artery—LM	2877 (2%)	74 (3.5%)	0.00031	63 (3.2%)	11 (6.5%)	0.025
Infarct-related artery—LAD	48,046 (34%)	806 (37.7%)	<0.0001	730 (37.1%)	76 (44.7%)	0.049
Infarct-related artery—Cx	27,891 (19.7%)	342 (16%)	0.29	321 (16.3%)	21 (12.4%)	0.18
Infarct-related artery—bypass	1530 (1.1%)	18 (0.8%)	<0.0001	16 (0.8%)	2 (1.2%)	0.62
PCI	118,119 (76.6%)	1956 (85.2%)	<0.0001	1802 (85.6%)	154 (81.1%)	0.096
TIMI 1 before PCI	72,425 (63.1%)	1450 (77.7%)	<0.0001	1344 (78.5%)	106 (68.8%)	0.006
TIMI 3 after PCI	107,885 (93.6%)	1704 (91.3%)	0.044	1569 (91.6%)	135 (87.7%)	0.098
CABG	4297 (2.8%)	48 (2.1%)	<0.0001	46 (2.2%)	2 (1.1%)	0.44
Pacemaker	810 (0.5%)	36 (1.6%)	<0.0001	28 (1.3%)	8 (4.2%)	0.0022
ICD	243 (0.2%)	60 (2.6%)	<0.0001	34 (1.6%)	26 (13.7%)	<0.0001
CRT-D	25 (0%)	7 (0.3%)	<0.0001	6 (0.3%)	1 (0.5%)	0.91
ICD or CRT-D	268 (0.2%)	67 (2.9%)	<0.0001	40 (1.9%)	27 (14.2%)	<0.0001
Blood transfusion	4912 (3.2%)	213 (9.3%)	<0.0001	171 (8.1%)	42 (22.1%)	<0.0001
Ablation	24 (0%)	4 (0.2%)	0.28	4 (0.2%)	0 (0%)	0.76
Heart valve surgery	286 (0.2%)	2 (0.1%)	<0.0001	2 (0.1%)	0 (0%)	0.39
IABP	1382 (0.9%)	167 (7.3%)	<0.0001	150 (7.1%)	17 (9%)	0.35

For abbreviations, see Table 1.

**Table 3:** In-hospital complications of patients without cardiac arrest and with ErCA and LrCA after acute myocardial infarction (only patients who were discharged after index hospitalisation).

Variable	Non-CA *N* = 154,266	rCA *N* = 2296	*p* Value	ErCA *N* = 2106	LrCA *N* = 190	*p* Value
Massive bleeding	1638 (1.1%)	114 (5%)	<0.0001	93 (4.4%)	21 (11.1%)	<0.0001
Recurrent myocardial infarction	274 (0.2%)	28 (1.2%)	<0.0001	19 (0.9%)	9 (4.7%)	<0.0001
Stroke	241 (0.2%)	31 (1.4%)	<0.0001	29 (1.4%)	2 (1.1%)	0.97
Pulmonary edema	1088 (0.7%)	102 (4.4%)	<0.0001	87 (4.1%)	15 (7.9%)	0.016
Cardiogenic shock	661 (0.4%)	227 (9.9%)	<0.0001	204 (9.7%)	23 (12.1%)	0.28
Hospital CA	0 (0%)	1216 (53.0%)	<0.0001	1026 (48.7%)	190 (100%)	<0.0001
Mechanical complication: heart rupture	28 (0%)	5 (0.2%)	0.0037	4 (0.2%)	1 (0.5%)	0.89
Mechanical complication: mitral regurgitation	42 (0%)	3 (0.1%)	0.54	3 (0.1%)	0 (0%)	0.6
Mechanical complication: VSD	25 (0%)	0 (0%)	<0.0001	0 (0%)	0 (0%)	1.00
Mechanical complication: heart rupture or VSD	51 (0%)	5 (0.2%)	<0.0001	4 (0.2%)	1 (0.5%)	0.89
Mechanical complications (all)	92 (0.1%)	8 (0.4%)	<0.0001	7 (0.3%)	1 (0.5%)	0.84

For abbreviations, see Table 1.

**Table 4:** Discharge data of patients without cardiac arrest and with ErCA and LrCA after acute myocardial infarction (only patients who were discharged after index hospitalisation).

Variable	Non-CA *N* = 154,266	rCA *N* = 2296	*p* Value	ErCA *N* = 2106	LrCA *N* = 190	*p* Value
LVEF, %, mean (SD)	47.9 (10.6)	43.2 (12.0)	<0.0001	43.7 (11.8)	38.4 (12.9)	<0.0001
Hospitalisation length, days, median (IQR)	5 (3–7)	7 (4–11)	0.0031	6 (4–10)	12 (8–19)	<0.0001
Aspirin at discharge	141,512 (91.7%)	2069 (90.1%)	0.0052	1893 (89.9%)	176 (92.6%)	0.22
P2Y12 inhibitor at discharge	130,658 (84.7%)	1955 (85.2%)	0.55	1786 (84.8%)	169 (89%)	0.12
Acenocoumarol/warfarin at discharge	3727 (2.4%)	68 (3%)	0.091	57 (2.7%)	11 (5.8%)	0.016
Beta-blocker at discharge	128,332 (83.2%)	1822 (79.4%)	<0.0001	1665 (79.1%)	157 (82.6%)	0.24
LMWH at discharge	7399 (4.8%)	197 (8.6%)	<0.0001	176 (8.4%)	21 (11.1%)	0.2
ACE-I at discharge	119,911 (77.7%)	1693 (73.7%)	<0.0001	1554 (73.8%)	139 (73.2%)	0.85
ARB at discharge	3765 (2.4%)	46 (2%)	0.18	41 (2%)	5 (2.6%)	0.71
Fibrate at discharge	1688 (1.1%)	18 (0.8%)	0.16	18 (0.9%)	0 (0%)	0.39
Statin at discharge	135,497 (87.8%)	1966 (85.6%)	0.0014	1803 (85.6%)	163 (85.8%)	0.95
Calcium channel blocker at discharge	14,877 (9.6%)	133 (5.8%)	<0.0001	116 (5.5%)	17 (9%)	0.052
Nitrate at discharge	17,305 (11.2%)	188 (8.2%)	<0.0001	168 (8%)	20 (10.5%)	0.22
Diuretic at discharge	38,629 (25%)	773 (33.7%)	<0.0001	675 (32.1%)	98 (51.6%)	<0.0001
Diabetes treated with insulin at discharge	13,410 (8.7%)	189 (8.2%)	0.44	161 (7.6%)	28 (14.7%)	0.0007
Diabetes treated with oral medication at discharge	14,642 (9.5%)	154 (6.7%)	<0.0001	132 (6.3%)	22 (11.6%)	0.0051

For abbreviations, see Table 1.

**Table 5:** One-year follow-up outcomes of patients without cardiac arrest and with ErCA and LrCA after acute myocardial infarction (only patients who were discharged after index hospitalisation).

Variable	Non-CA *N* = 154,266	rCA *N* = 2296	*p* Value	ErCA *N* = 2106	LrCA *N* = 190	*p* Value
Mortality after discharge						
Death: 30 days	1765 (1.1%)	53 (2.3%)	<0.0001	47 (2.2%)	6 (3.2%)	0.42
Death: 6 months	7942 (5.2%)	228 (9.9%)	<0.0001	194 (9.2%)	34 (17.9%)	0.0001
Death: 12 months	11,947 (7.7%)	299 (13.0%)	<0.0001	259 (12.3%)	40 (21.1%)	0.0006
Re-hospitalisation with main diagnosis						
All cause	88,016 (57.1%)	1348 (58.7%)	0.11	1224 (58.1%)	124 (65.3%)	0.055
Cardiovascular cause	71,290 (46.2%)	1095 (47.7%)	0.15810	1000 (47.5%)	95 (50%)	0.51
Chronic coronary syndrome	36,659 (23.8%)	557 (24.3%)	0.57925	530 (25.2%)	27 (14.2%)	0.0007
Unstable angina	19,390 (12.6%)	225 (9.8%)	0.00007	213 (10.1%)	12 (6.3%)	0.092
Myocardial infarction	8552 (5.5%)	109 (4.8%)	0.09758	96 (4.6%)	13 (6.8%)	0.16
Chronic coronary syndrome or unstable angina or myocardial infarction	55,008 (35.7%)	766 (33.4%)	0.02262	720 (34.2%)	46 (24.2%)	0.0052
Heart failure	12,491 (8.1%)	262 (11.4%)	<0.0001	228 (10.8%)	34 (17.9%)	0.0033
Stroke	2205 (1.4%)	23 (1%)	0.08595	20 (1%)	3 (1.6%)	0.65
Cardiac rehabilitation after 30 days	32,079 (20.8%)	541 (23.6%)	0.00119	511 (24.3%)	30 (15.8%)	0.0084
Cardiac rehabilitation after 6 months	35,748 (23.2%)	592 (25.8%)	0.00326	561 (26.6%)	31 (16.3%)	0.0018
Cardiac rehabilitation after 12 months	37,258 (24.2%)	614 (26.7%)	0.00401	580 (27.5%)	34 (17.9%)	0.004
Re-hospitalisation with procedure						
Coronary angiography	39,549 (25.6%)	559 (24.4%)	0.15983	528 (25.1%)	31 (16.3%)	0.0071
PCI	31,015 (20.1%)	376 (16.4%)	0.00001	361 (17.1%)	15 (7.9%)	0.001
CABG	8081 (5.2%)	112 (4.9%)	0.44160	103 (4.9%)	9 (4.7%)	0.92
Pacemaker implantation	1276 (0.8%)	18 (0.8%)	0.82060	17 (0.8%)	1 (0.5%)	0.99
CRT-P implantation	51 (0.03%)	0 (0%)	0.38355	0 (0%)	0 (0%)	1.00
ICD implantation	2171 (1.4%)	94 (4.1%)	<0.0001	83 (3.9%)	11 (5.8%)	0.22
CRT-D implantation	365 (0.2%)	18 (0.8%)	<0.0001	17 (0.8%)	1 (0.5%)	0.99
ICD or CRT-D implantation	2531 (1.6%)	111 (4.8%)	<0.0001	99 (4.7%)	12 (6.3%)	0.32

For abbreviations, see Table 1.

## Data Availability

No data available.

## Supplementary Materials

The following supporting information can be downloaded at: https://www.mdpi.com/article/10.3390/jcm11030609/s1, Figure S1: Multivariate logistic regression model of factors affecting in-hospital mortality in the whole cohort of patients; Figure S2: Risk factors of the occurrence of resuscitated cardiac arrest (both early or late) in the cohort of patients that survived in-hospital period multivariate logistic regression model; Figure S3: 1-year survival (A), rehospitalizations (B-H) and procedures (I-K) in acute myocardial infarction patients discharged home who survived cardiac arrest (early or late) compared to patients without cardiac arrest; Table S1: Baseline clinical characteristics of patients without cardiac arrest and with ErCA and LrCA in the whole cohort of patients with acute myocardial infarction.
