# Supplementary material for: In-Hospital and One-Year Outcomes of Patients after Early and Late Resuscitated Cardiac Arrest Complicating Acute Myocardial Infarction—Data from a Nationwide Database
Source: J Clin Med. 2022 Jan 26;11(3):609. doi: 10.3390/jcm11030609 (PMC8836754; doi:10.3390/jcm11030609)
Supplement: Supplementary file 1 [file jcm-11-00609-s001.zip › jcm-1517013-supplementary.pdf]

Supplementary Material

Supplementary Table S1. Baseline clinical characteristics of patients without cardiac arrest and with ErCA and LrCA in the whole cohort of patients with acute myocardial infarction.

Variable	Non-CA N=164057	rCA N=3564	*P value*	ErCA N=3100	LrCA N=464	*P value*
Age ≥ 65 years	87880 (53.57%)	1910 (53.59%)	0.97695	1567 (50.6%)	343 (73.9%)	<0.0001
Age ≥ 75 years	47236 (28.79%)	1071 (30.05%)	0.10094	844 (27.2%)	227 (48.9%)	<0.0001
Age ≥ 85 years	9179 (5.6%)	235 (6.59%)	0.01041	175 (5.7%)	60 (12.9%)	<0.0001
Male gender	104179 (63.6%)	2326 (65.34%)	0.03264	2049 (66.2%)	277 (59.7%)	0.0062
From home admission	93941 (57.3%)	2252 (63.22%)	<0.0001	1960 (63.3%)	292 (62.9%)	0.89
Transfer from other hospital	70013 (42.7%)	1310 (36.78%)	<0.0001	1138 (36.7%)	172 (37.1%)	0.89
Hipercholesterolemia	67362 (41.06%)	1242 (34.85%)	<0.0001	1079 (34.8%)	163 (35.1%)	0.89
Hypertension	118610 (72.3%)	2247 (63.05%)	<0.0001	1918 (61.9%)	329 (70.9%)	0.0002
Obesity	33764 (20.58%)	721 (20.23%)	0.60851	617 (19.9%)	104 (22.4%)	0.21
Diabetes mellitus	42119 (26.47%)	900 (26.12%)	0.65383	719 (24.1%)	181 (39.8%)	<0.0001
Smoking (current or past)	96191 (58.63%)	2142 (60.1%)	0.07823	1900 (61.3%)	242 (52.2%)	0.0002
Current smoking	47571 (29%)	1078 (30.25%)	0.10374	993 (32%)	85 (18.3%)	<0.0001
Ischemic heart disease diagnosed before AMI	23693 (14.44%)	667 (18.71%)	0.62379	562 (18.1%)	105 (22.6%)	0.20
Family history of CAD	18289 (11.15%)	388 (10.89%)	<0.0001	331 (10.7%)	57 (12.3%)	0.30
History of heart failure	13436 (8.19%)	418 (11.73%)	<0.0001	319 (10.3%)	99 (21.3%)	<0.0001
History of stroke	6497 (3.96%)	205 (5.75%)	<0.0001	170 (5.5%)	35 (7.5%)	0.076
Chronic kidney disease	10871 (6.63%)	383 (10.75%)	<0.0001	281 (9.1%)	102 (22%)	<0.0001
Peripheral artery disease	7927 (4.83%)	234 (6.57%)	<0.0001	189 (6.1%)	45 (9.7%)	0.0035
Chronic obstructive pulmonary disease	6674 (4.07%)	201 (5.64%)	<0.0001	160 (5.2%)	41 (8.8%)	0.0014
Prior myocardial infarction	29184 (17.79%)	727 (20.4%)	0.00006	619 (20%)	108 (23.3%)	0.099
Prior PCI	20749 (12.65%)	428 (12.01%)	0.25638	370 (11.9%)	58 (12.5%)	0.73
Prior CABG	5348 (3.26%)	119 (3.34%)	0.79254	99 (3.2%)	20 (4.3%)	0.21
NSTEMI	86909 (52.97%)	1208 (33.89%)	<0.0001	1011 (32.6%)	197 (42.5%)	<0.0001
STEMI	77148 (47.03%)	2356 (66.11%)	<0.0001	2089 (67.4%)	267 (57.5%)	<0.0001
CA before admission	684 (0.42%)	1603 (44.98%)	<0.0001	1587 (51.2%)	16 (3.5%)	<0.0001
Sinus rhythm	148001 (90.8%)	2884 (81.01%)	<0.0001	2523 (81.5%)	361 (77.8%)	0.059
Atrial fibrillation	9950 (6.1%)	365 (10.25%)	<0.0001	301 (9.7%)	64 (13.8%)	0.0070
Other rhythm	3053 (1.87%)	244 (6.85%)	<0.0001	220 (7.1%)	24 (5.2%)	0.12
Normal QRS	134312 (82.53%)	2585 (72.96%)	<0.0001	2265 (73.5%)	320 (69.1%)	0.046
LBBB	6661 (4.09%)	231 (6.52%)	<0.0001	197 (6.4%)	34 (7.3%)	0.44
RBBB	5890 (3.62%)	176 (4.97%)	0.00002	141 (4.6%)	35 (7.6%)	0.0059
Other QRS abnormalities	15889 (9.76%)	551 (15.55%)	<0.0001	477 (15.5%)	74 (16%)	0.78
Normal ST segment	12740 (7.8%)	126 (3.54%)	<0.0001	102 (3.3%)	24 (5.2%)	0.041
ST segment elevation	75016 (45.93%)	2270 (63.71%)	<0.0001	2019 (65.2%)	251 (54.1%)	<0.0001
ST segment depression	38866 (23.8%)	621 (17.43%)	<0.0001	522 (16.8%)	99 (21.3%)	0.017
Negative T waves	14509 (8.88%)	121 (3.4%)	<0.0001	98 (3.2%)	23 (5%)	0.047
Other ST segment abnormalities	21099 (12.92%)	410 (11.51%)	0.01288	346 (11.2%)	64 (13.8%)	0.098
Killip class 1	124975 (76.64%)	1575 (44.22%)	<0.0001	1365 (44.1%)	210 (45.3%)	0.63
Killip class 2	22920 (14.06%)	789 (22.15%)	<0.0001	650 (21%)	139 (30%)	<0.0001
Killip class 3	4676 (2.87%)	254 (7.13%)	<0.0001	204 (6.6%)	50 (10.8%)	0.0011
Killip class 4	4674 (2.87%)	825 (23.16%)	<0.0001	781 (25.2%)	44 (9.5%)	<0.0001
Thrombolysis	609 (0.39%)	30 (0.89%)	<0.0001	26 (0.9%)	4 (0.9%)	0.84
Glycoprotein IIb/IIIa inhibitors	35829 (21.84%)	1255 (35.21%)	<0.0001	1110 (35.8%)	145 (31.3%)	0.055
Anticoagulation (not associated with PCI)	84890 (52.08%)	2020 (56.71%)	<0.0001	1730 (55.8%)	290 (62.5%)	0.0069
Coronary angiography	149224 (90.99%)	3239 (90.93%)	0.90814	2839 (91.6%)	400 (86.2%)	0.0001
Infarct-related artery – RCA	44659 (29.92%)	1039 (32.06%)	0.00848	932 (32.8%)	107 (26.8%)	0.015
Infarct-related artery – LM	3556 (2.38%)	159 (4.91%)	<0.0001	130 (4.6%)	29 (7.3%)	0.020
Infarct-related artery – LAD	51152 (34.27%)	1243 (38.35%)	<0.0001	1075 (37.8%)	168 (42%)	0.11
Infarct-related artery – Cx	28996 (19.42%)	517 (15.95%)	<0.0001	457 (16.1%)	60 (15%)	0.58
Infarct-related artery – bypass	1591 (1.07%)	28 (0.86%)	0.26724	24 (0.8%)	4 (1%)	0.98
PCI	124365 (75.81%)	2920 (81.93%)	<0.0001	2570 (82.9%)	350 (75.4%)	0.0001
TIMI 0 or 1 before PCI	76990 (63.71%)	2157 (76.95%)	<0.0001	1921 (78.1%)	236 (68.6%)	<0.0001
TIMI 3 after PCI	112057 (92.35%)	2419 (86.24%)	<0.0001	2128 (86.5%)	291 (84.4%)	0.28
CABG	4627 (2.82%)	58 (1.63%)	0.00002	53 (1.7%)	5 (1.1%)	0.32
Pacemaker	842 (0.51%)	44 (1.23%)	<0.0001	33 (1.1%)	11 (2.4%)	0.017
ICD	249 (0.15%)	62 (1.74%)	<0.0001	35 (1.1%)	27 (5.8%)	<0.0001
CRT-D	25 (0.02%)	7 (0.2%)	<0.0001	6 (0.2%)	1 (0.2%)	0.64
ICD or CRT-D	274 (0.17%)	69 (1.94%)	<0.0001	41 (1.3%)	28 (6%)	<0.0001
Blood transfusion	6087 (3.71%)	412 (11.56%)	<0.0001	325 (10.5%)	87 (18.8%)	<0.0001
Ablation	27 (0.02%)	4 (0.11%)	0.00003	4 (0.1%)	0 (0%)	0.98
Heart valve surgery	360 (0.22%)	8 (0.22%)	0.94938	7 (0.2%)	1 (0.2%)	0.63
IABP	2700 (1.65%)	406 (11.39%)	<0.0001	349 (11.3%)	57 (12.3%)	0.52
Massive bleeding	2013 (1.23%)	228 (6.4%)	<0.0001	175 (5.7%)	53 (11.4%)	<0.0001
Recurrent myocardial infarction	408 (0.25%)	67 (1.88%)	<0.0001	42 (1.4%)	25 (5.4%)	<0.0001

Stroke	415 (0.25%)	51 (1.43%)	<0.0001	44 (1.4%)	7 (1.5%)	0.88
Pulmonary edema	1840 (1.12%)	263 (7.38%)	<0.0001	203 (6.6%)	60 (12.9%)	<0.0001
Cardiogenic shock	2545 (1.55%)	686 (19.25%)	<0.0001	568 (18.3%)	118 (25.4%)	0.0003
Hospital CA	3002 (1.83%)	2224 (62.4%)	<0.0001	1766 (57%)	458 (98.7%)	<0.0001
Mechanical complication: Heart rapture	135 (0.08%)	24 (0.67%)	<0.0001	16 (0.5%)	8 (1.7%)	0.0030
Mechanical complication: Mitral regurgitation	65 (0.04%)	4 (0.11%)	0.03450	4 (0.1%)	0 (0%)	0.98
Mechanical complication: VSD	83 (0.05%)	3 (0.08%)	0.38109	2 (0.1%)	1 (0.2%)	0.85
Mechanical complication: Heart rapture or VSD	214 (0.13%)	27 (0.76%)	<0.0001	18 (0.6%)	9 (1.9%)	0.0042
Mechanical complications (all)	277 (0.17%)	30 (0.84%)	<0.0001	21 (0.7%)	9 (1.9%)	0.012
In-hospital death	9790 (5.97%)	1268 (35.58%)	<0.0001	994 (32.1%)	274 (59.1%)	<0.0001

Abbreviations: ACE-I – angiotensin converting enzyme inhibitor; AMI – acute myocardial infarction; ARB – angiotensin receptor blocker; CA – cardiac arrest; CABG – coronary artery bypass grafting; CAD – coronary artery disease; ErCA – early resuscitated cardiac arrest; CRT-D – cardiac resynchronization therapy defibrillator; CRT-P – cardiac resynchronization therapy pacemaker; Cx – circumflex artery; IABP – intra-aortic balloon pump; ICD – implantable cardioverter defibrillator; ECG – electrocardiogram; LAD – left anterior descending; LBBB – left bundle branch block; LM – left main; LMWH – low molecular weight heparin; LrCA - late resuscitated cardiac arrest; NSTEMI – nonST segment myocardial infarction; PCI – percutaneous coronary intervention; RCA – right coronary artery; RBBB – right bundle branch block; rCA - resuscitated cardiac arrest; SD – standard deviation; STEMI – ST segment myocardial infarction; TIMI – The thrombolysis in myocardial infarction risk score; VSD – ventricular septal defect;.

Supplementary Figure S1.

Multivariate logistic regression model of factors affecting in-hospital mortality in the whole cohort of patients.

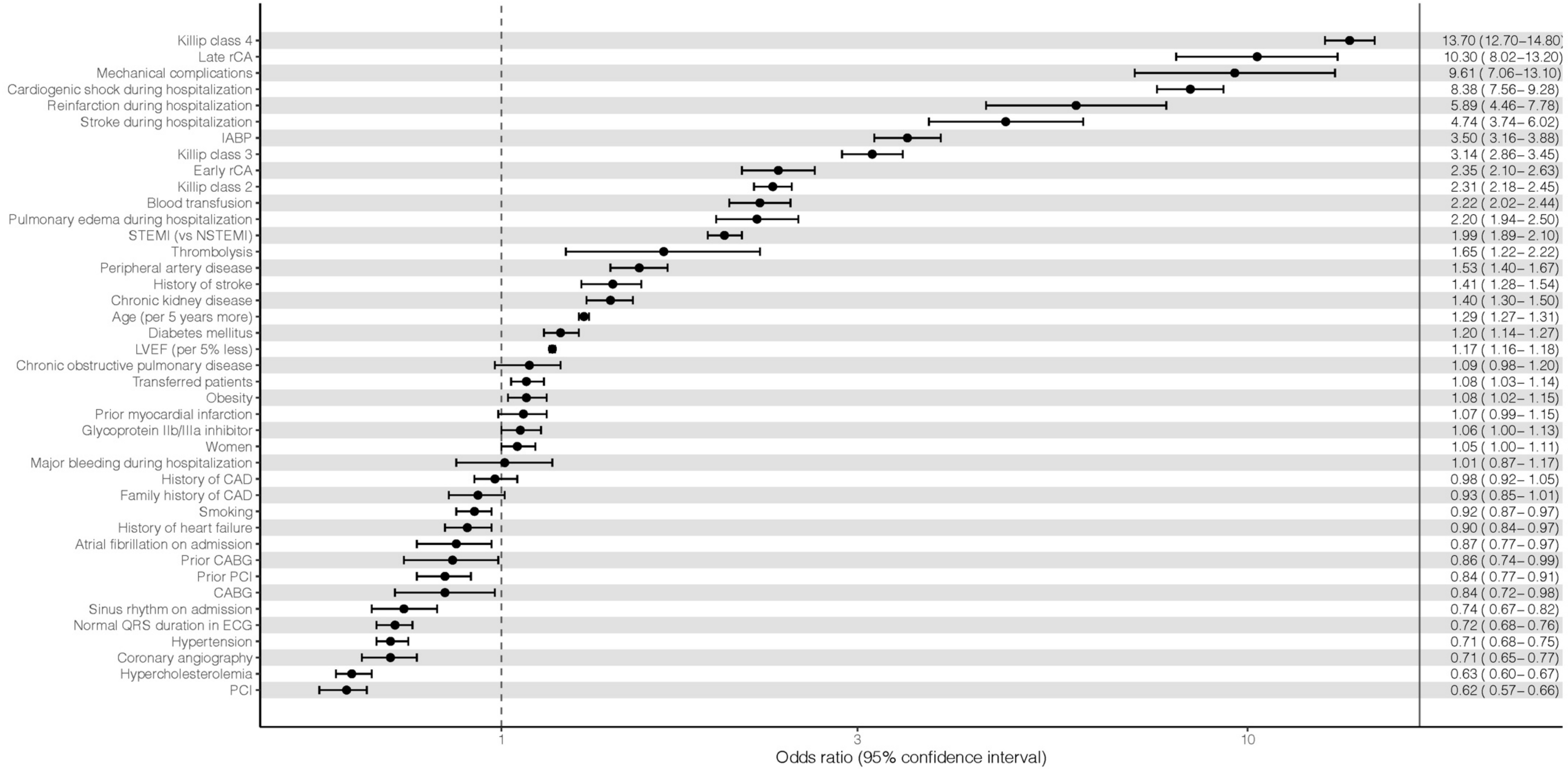

Supplementary Figure S2.

Risk factors of the occurrence of resuscitated cardiac arrest (both early or late) in the cohort of patients that survived in-hospital period multivariate logistic regression model.

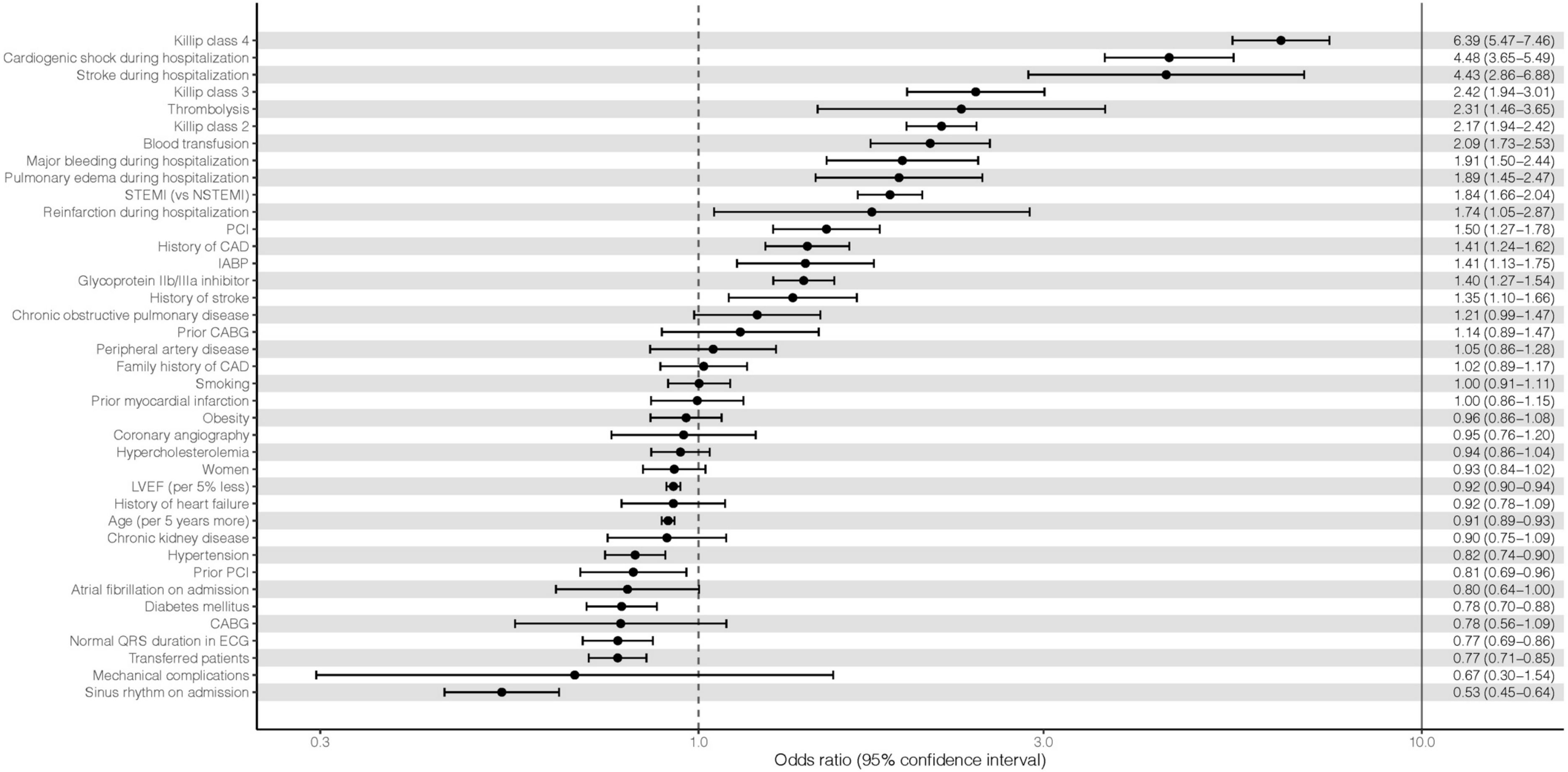

Supplementary Figure S3.

1-year survival (A), rehospitalizations (B-H) and procedures (I-K) in acute myocardial infarction patients discharged home who survived cardiac arrest (early or late) compared to patients without cardiac arrest.

A) 1-year survival after discharge

Unadjusted

	1-year survival (95% CI)	Hazard ratio (95% CI)	P value
**Non-CA**	0.923 (0.921 - 0.924)	1	
**rCA**	0.870 (0.856 - 0.884)	1.75 (1.56 – 1.96)	<0.0001
**ErCA**	0.877 (0.863 - 0.891)	1.64 (1.45 – 1.85)	<0.0001
**LrCA**	0.789 (0.734 – 0.850)	2.99 (2.19 – 4.07)	<0.0001

CI – confidence interval; CA – cardiac arrest; rCA – resuscitated cardiac arrest; ErCA – early resuscitated cardiac arrest; LrCA – late resuscitated cardiac arrest

Adjusted for baseline characteristics and revascularization

	1-year survival (95% CI)	Hazard ratio (95% CI)	P value
**Non-CA**	0.922 (0.921 - 0.924)	1	
**rCA**	0.878 (0.858 - 0.897)	1.62 (1.37 – 1.92)	<0.0001
**ErCA**	0.880 (0.859 - 0.902)	1.58 (1.31 – 1.91)	<0.0001
**LrCA**	0.834 (0.764 – 0.911)	2.28 (1.40 – 3.72)	<0.0001

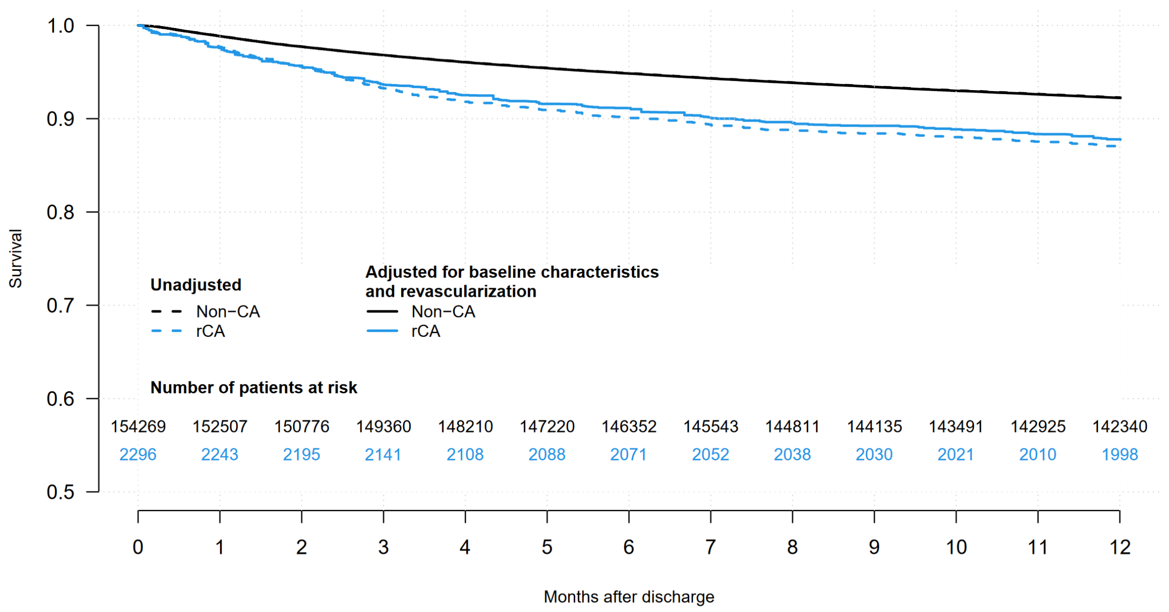

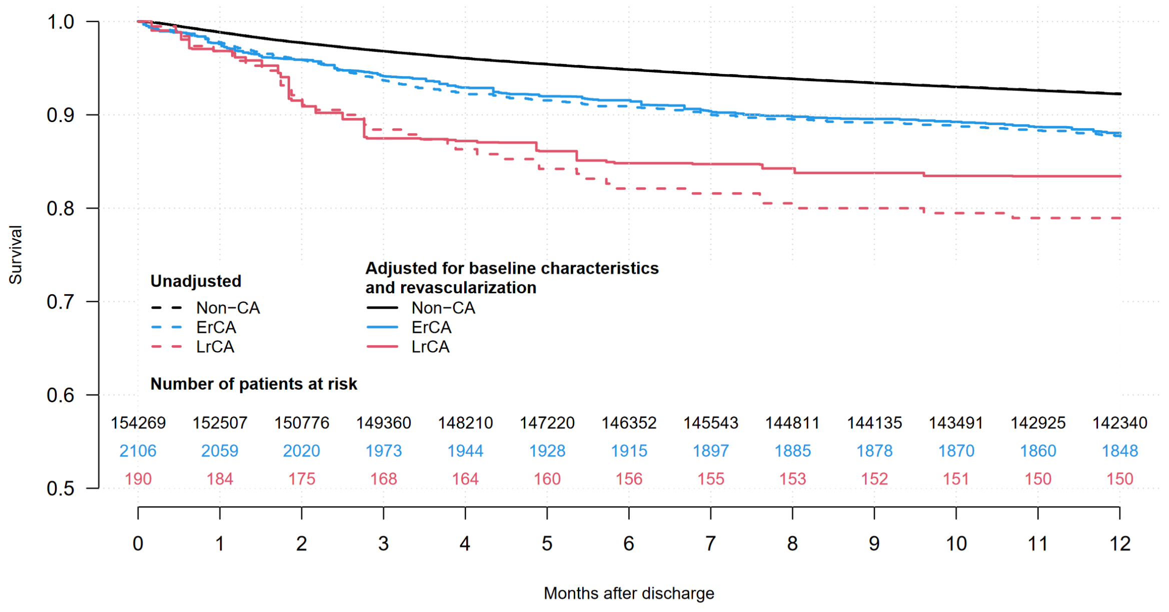

Adjusted for baseline characteristics, revascularization, and in−hospital complications

	1-year survival (95% CI)	Hazard ratio (95% CI)	P value
**Non-CA**	0.922 (0.920 - 0.923)	1	
**rCA**	0.880 (0.860 - 0.900)	1.58 (1.32 – 1.89)	<0.0001
**ErCA**	0.881 (0.860 - 0.904)	1.55 (1.28 – 1.90)	<0.0001
**LrCA**	0.840 (0.772 – 0.914)	2.17 (1.33 – 3.54)	0.00185

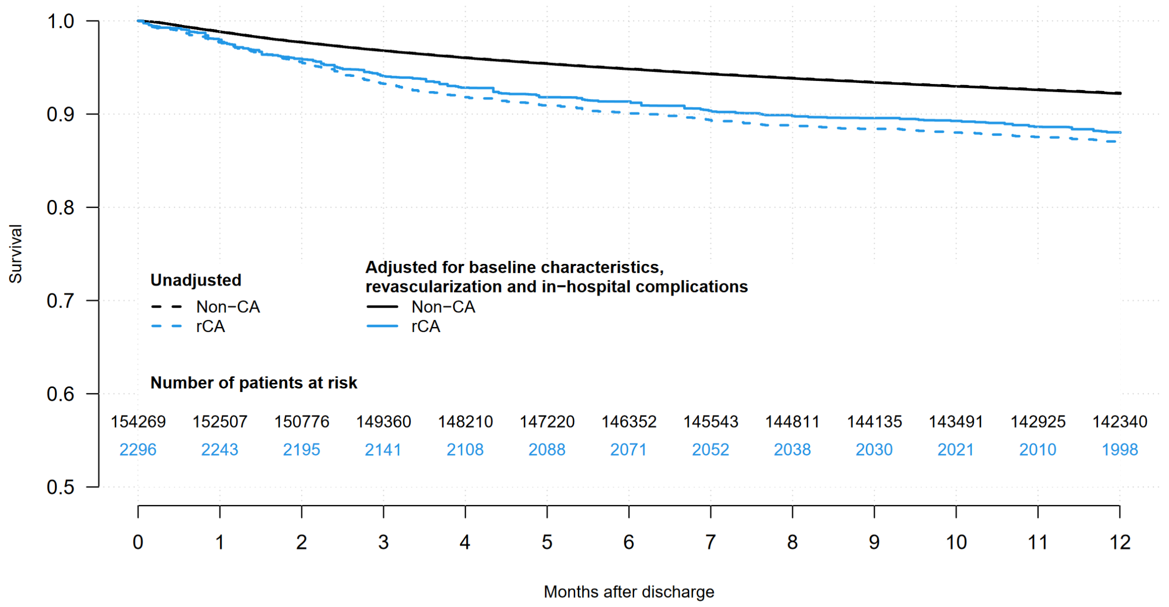

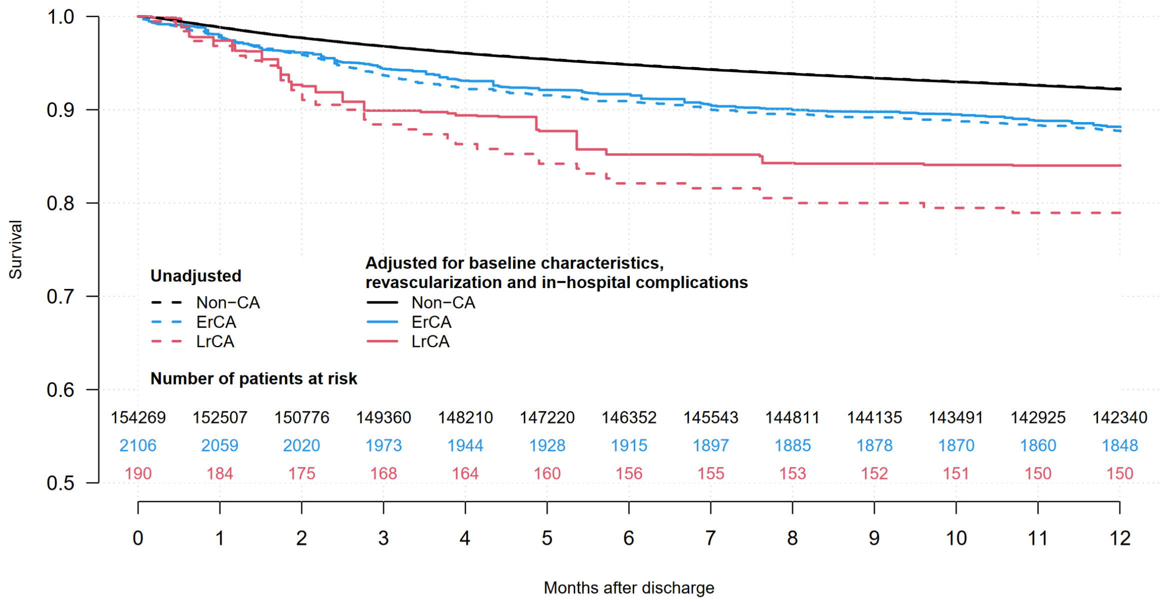

Adjusted for baseline characteristics, revascularization, in–hospital complications, and treatment procedures

	1-year survival (95% CI)	Hazard ratio (95% CI)	P value
**Non-CA**	0.922 (0.920 - 0.923)	1	
**rCA**	0.880 (0.860 - 0.901)	1.57 (1.30 – 1.87)	<0.0001
**ErCA**	0.882 (0.860 - 0.905)	1.54 (1.28 – 1.89)	<0.0001
**LrCA**	0.828 (0.751 – 0.913)	2.34 (1.39 – 3.93)	0.00134

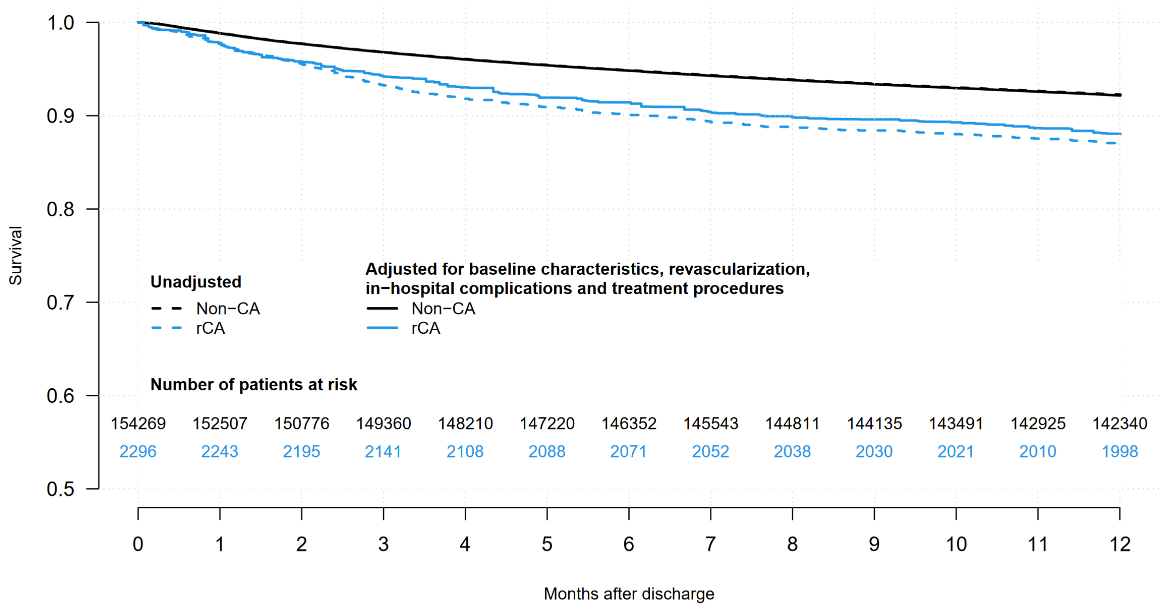

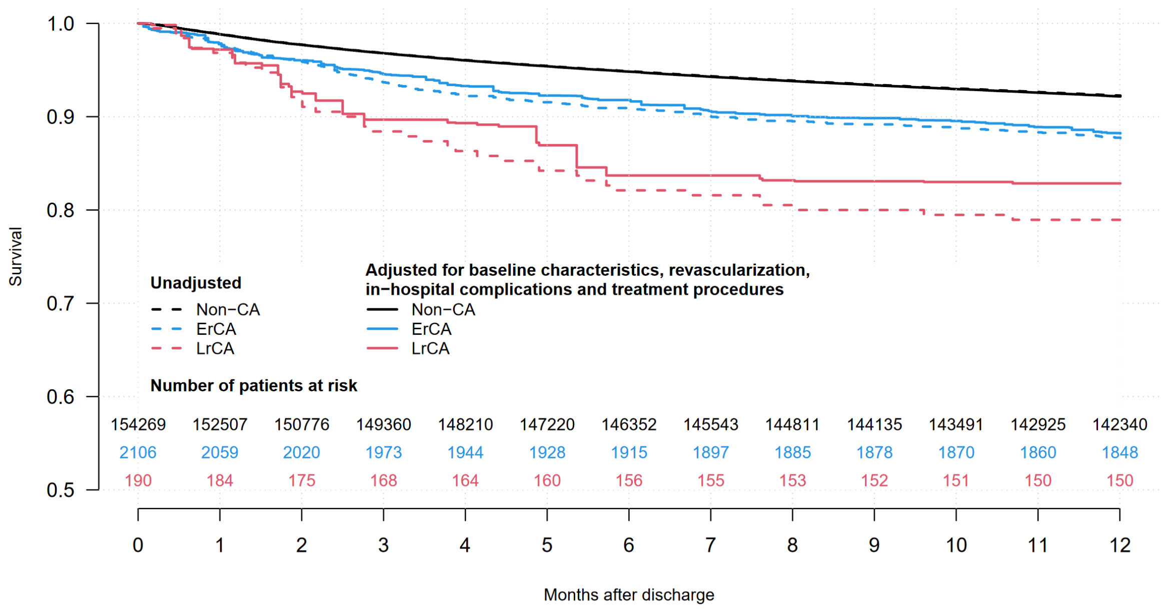

Relative risk of 1-year death from any reason

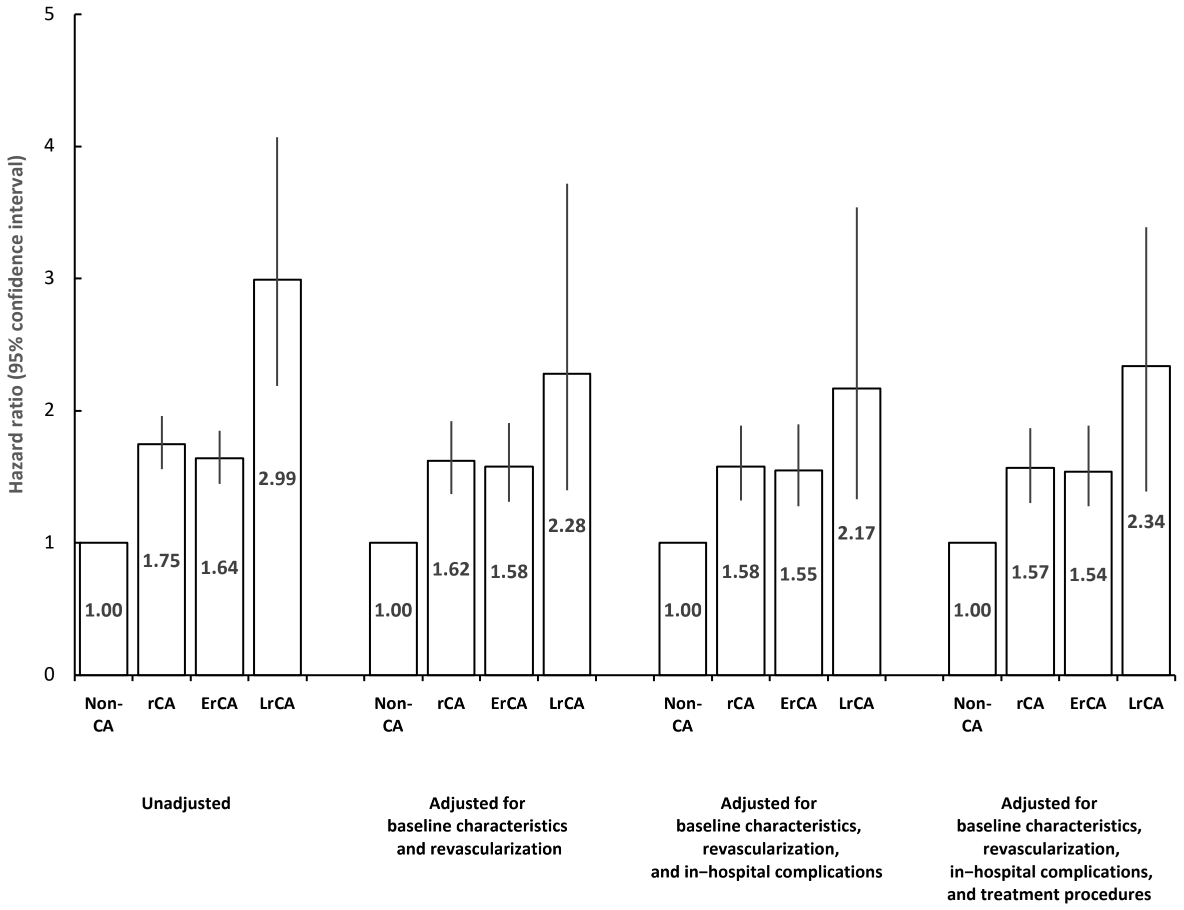

CA – cardiac arrest; rCA – resuscitated cardiac arrest; ErCA – early resuscitated cardiac arrest; LrCA – late resuscitated cardiac arrest

B) 1 year re-hospitalization due to acute myocardial infarction

Unadjusted

	1-year event-free (95% CI)	Hazard ratio (95% CI)	P value
**Non-CA**	0.944 (0.943 - 0.945)	1	
**rCA**	0.952 (0.943 - 0.961)	0.85 (0.71 – 1.03)	0.101
**ErCA**	0.954 (0.945 - 0.963)	0.82 (0.67 – 1.00)	0.0536
**LrCA**	0.932 (0.896 – 0.968)	1.23 (0.71– 2.11)	0.4637

CI – confidence interval; CA – cardiac arrest; rCA – resuscitated cardiac arrest; ErCA – early resuscitated cardiac arrest; LrCA – late resuscitated cardiac arrest

Adjusted for baseline characteristics and revascularization

	1-year event-free (95% CI)	Hazard ratio (95% CI)	P value
**Non-CA**	0.944 (0.943 - 0.946)	1	
**rCA**	0.951 (0.938 - 0.965)	0.88(0.66– 1.16)	0.365
**ErCA**	0.952 (0.937 - 0.967)	0.86 (0.63 – 1.19)	0.371
**LrCA**	0.931 (0.882 – 0.982)	1.25 (0.56– 2.62)	0.546

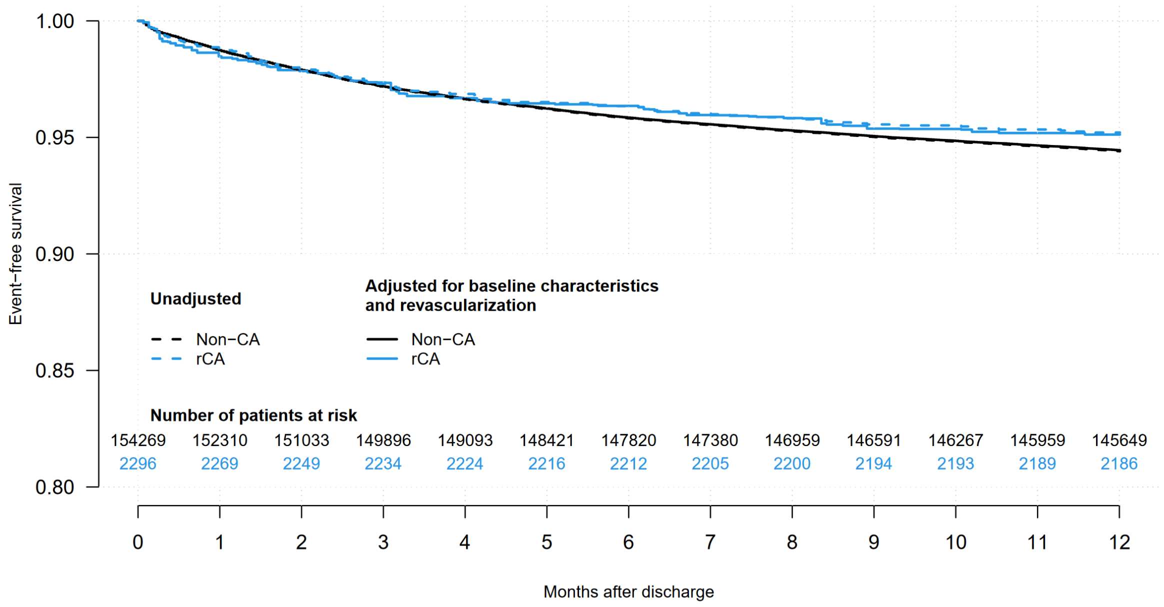

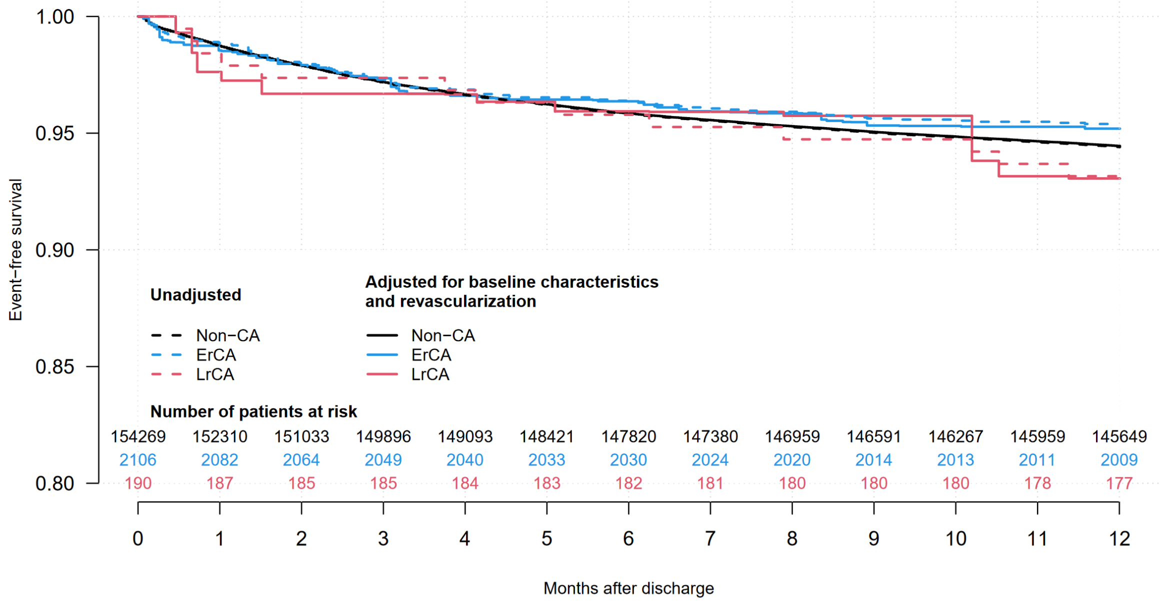

Adjusted for baseline characteristics, revascularization, and in−hospital complications

	1-year event-free (95% CI)	Hazard ratio (95% CI)	P value
**Non-CA**	0.945 (0.943 - 0.946)	1	
**rCA**	0.952 (0.939 - 0.966)	0.86 (0.65 – 1.15)	0.308
**ErCA**	0.952 (0.937 - 0.967)	0.87 (0.63– 1.19)	0.384
**LrCA**	0.929 (0.871 – 0.992)	1.28 (0.53– 3.06)	0.586

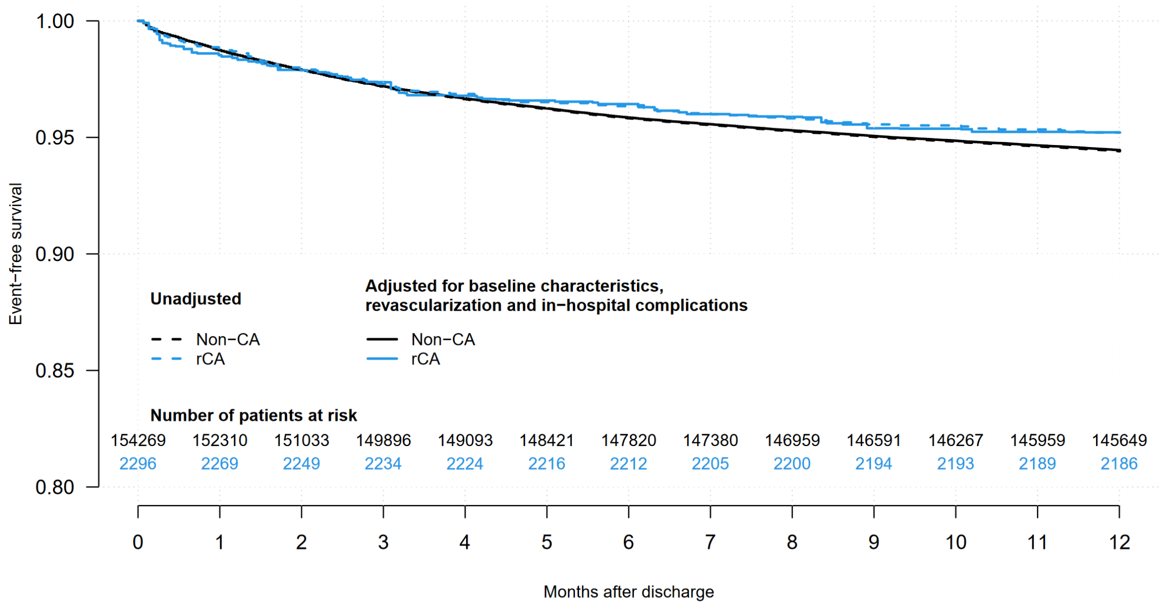

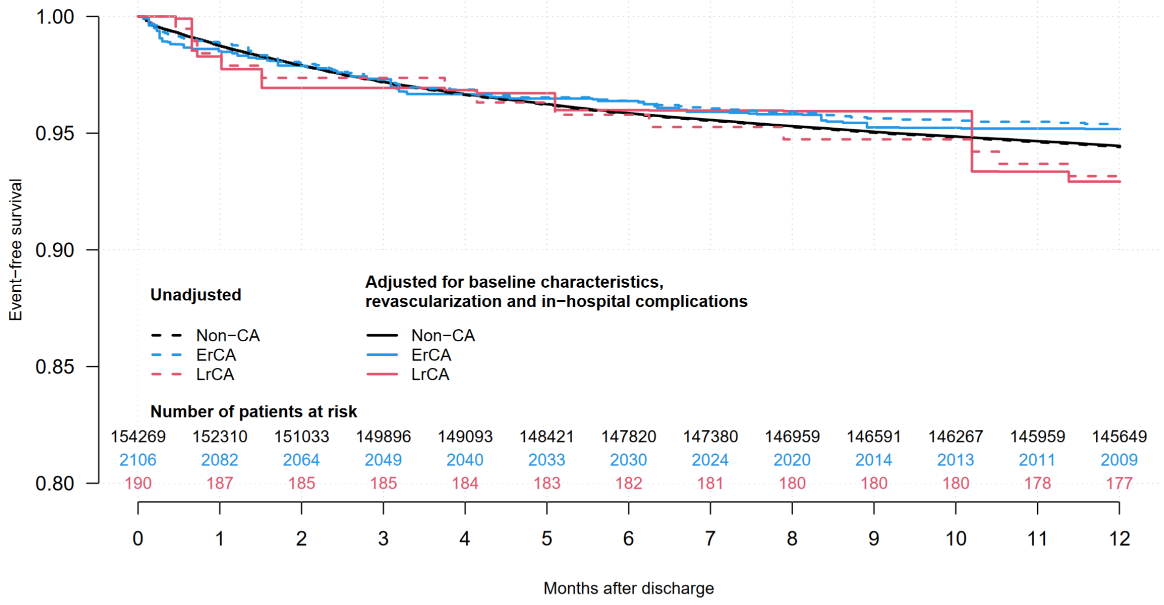

Adjusted for baseline characteristics, revascularization, in–hospital complications, and treatment procedures

	1-year event-free (95% CI)	Hazard ratio (95% CI)	P value
**Non-CA**	0.945 (0.943 – 0.946)	1	
**rCA**	0.952 (0.938 - 0.966)	0.87 (0.64 – 1.17)	0.348
**ErCA**	0.952 (0.936 - 0.967)	0.87 (0.63 – 1.21)	0.419
**LrCA**	0.922 (0.852 – 0.999)	1.40 (0.53 – 3.71)	0.497

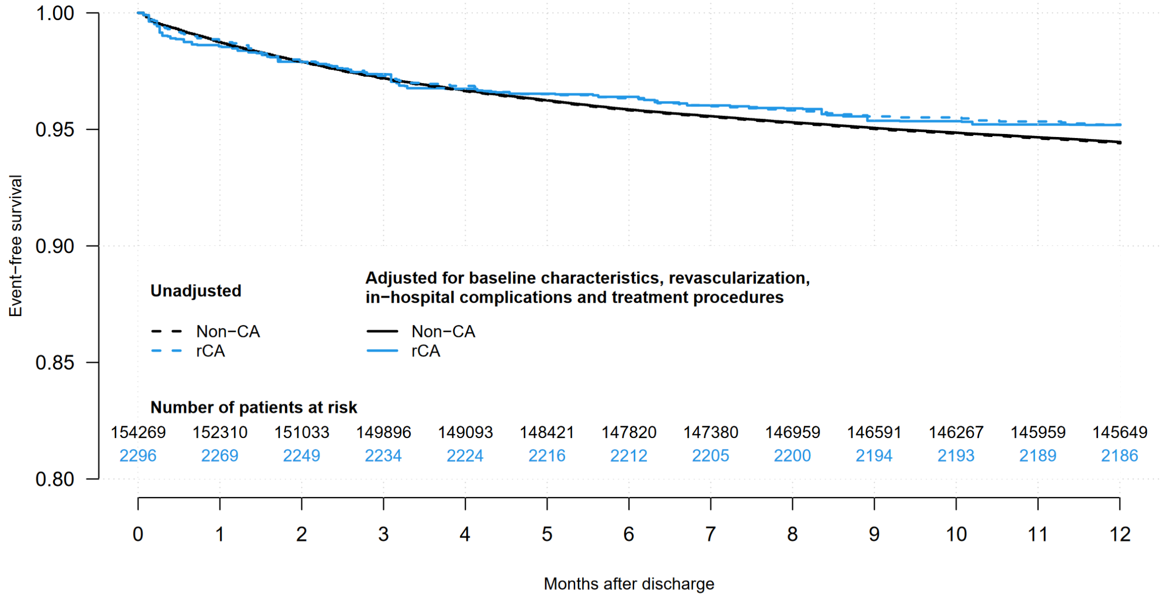

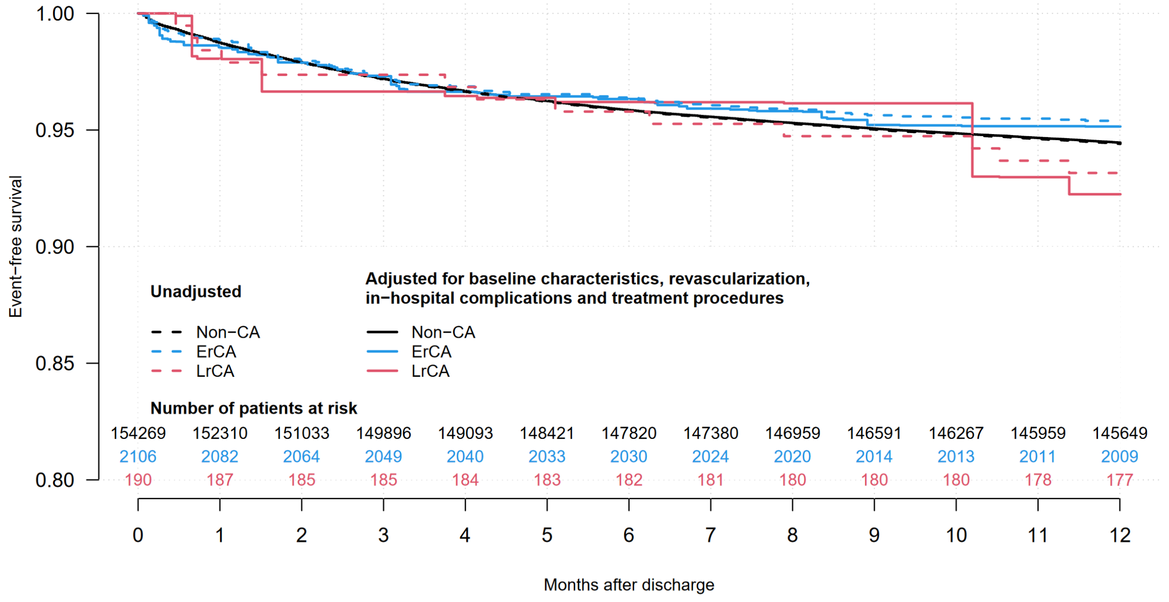

Relative risk of 1-year re-hospitalization due to acute myocardial infarction

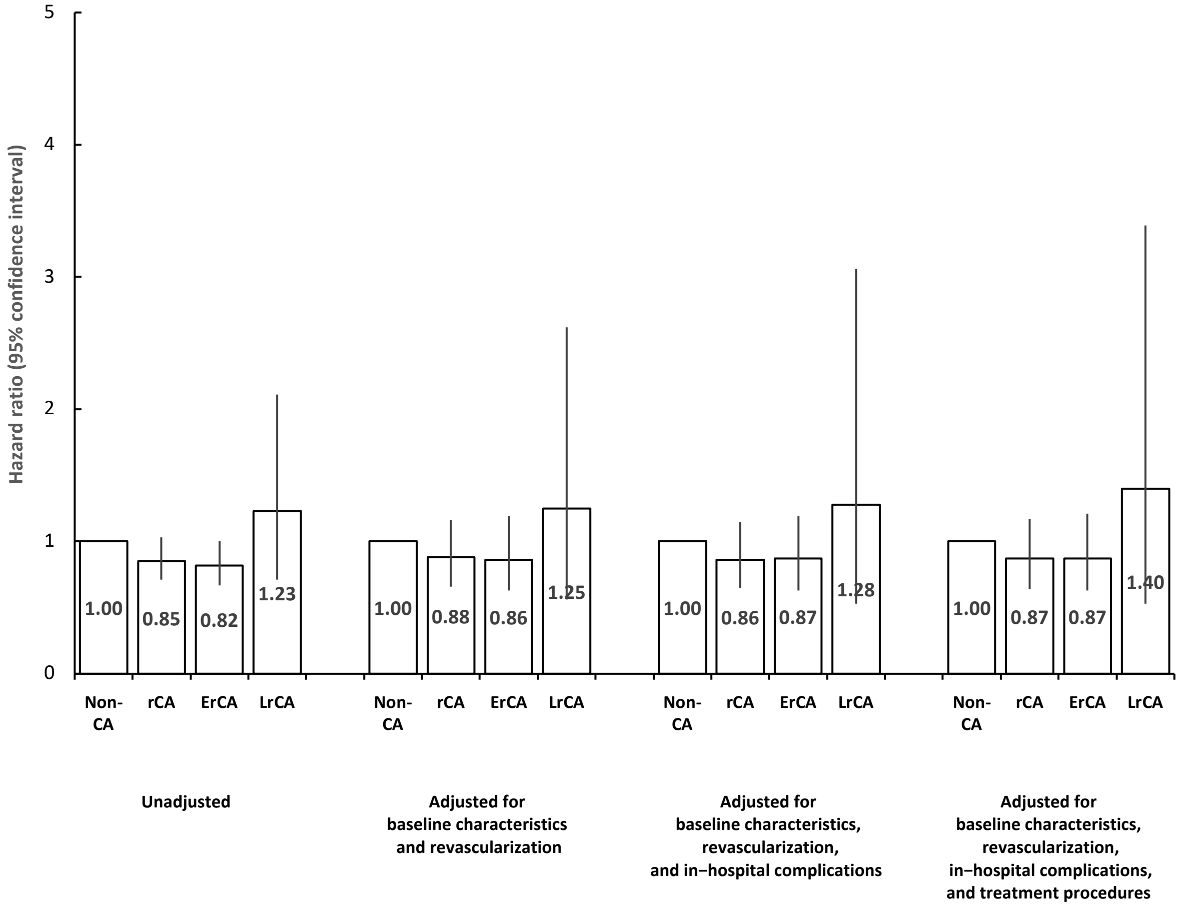

CA – cardiac arrest; rCA – resuscitated cardiac arrest; ErCA – early resuscitated cardiac arrest; LrCA – late resuscitated cardiac arrest

C) 1 year re-hospitalization due to unstable angina

Unadjusted

	1-year event-free (95% CI)	Hazard ratio (95% CI)	P value
**Non-CA**	0.874 (0.872 - 0.875)	1	
**rCA**	0.902 (0.889 - 0.914)	0.77 (0.67 – 0.87)	<0.0001
**ErCA**	0.898 (0.886 - 0.911)	0.79 (0.69 – 0.91)	0.0007
**LrCA**	0.937 (0.903 – 0.972)	0.48 (0.27– 0.85)	0.0111

CI – confidence interval; CA – cardiac arrest; rCA – resuscitated cardiac arrest; ErCA – early resuscitated cardiac arrest; LrCA – late resuscitated cardiac arrest

Adjusted for baseline characteristics and revascularization

	1-year event-free (95% CI)	Hazard ratio (95% CI)	P value
**Non-CA**	0.872 (0.870 - 0.873)	1	
**rCA**	0.889 (0.869 - 0.910)	0.86 (0.71– 1.04)	0.116
**ErCA**	0.886 (0.864 - 0.908)	0.89 (0.72 – 1.09)	0.244
**LrCA**	0.930 (0.877 – 0.987)	0.53 (0.23 – 1.21)	0.131

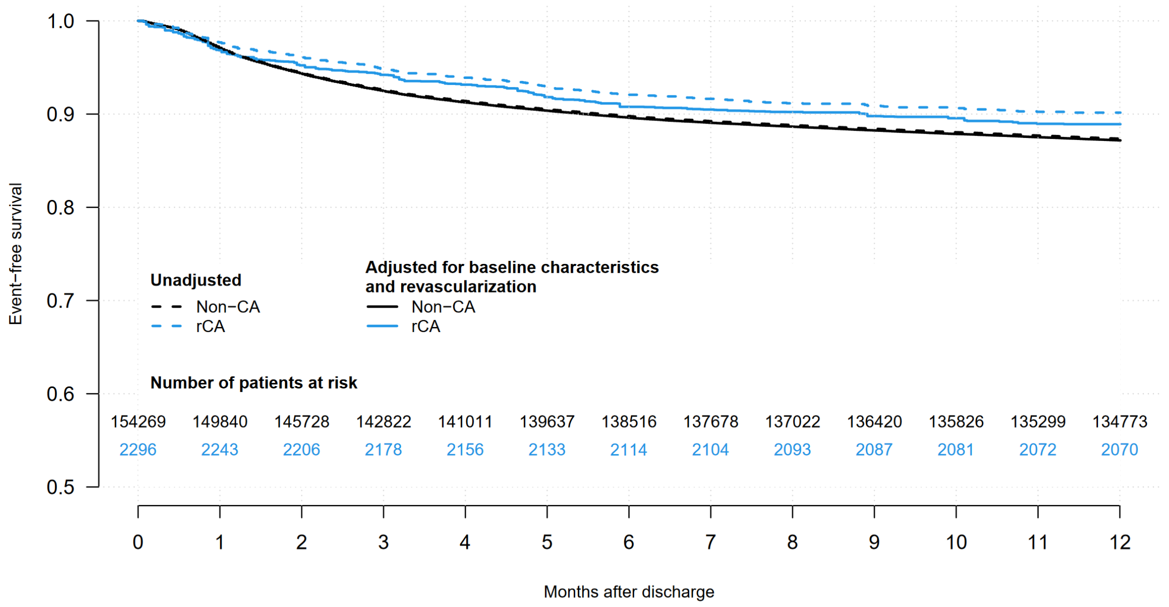

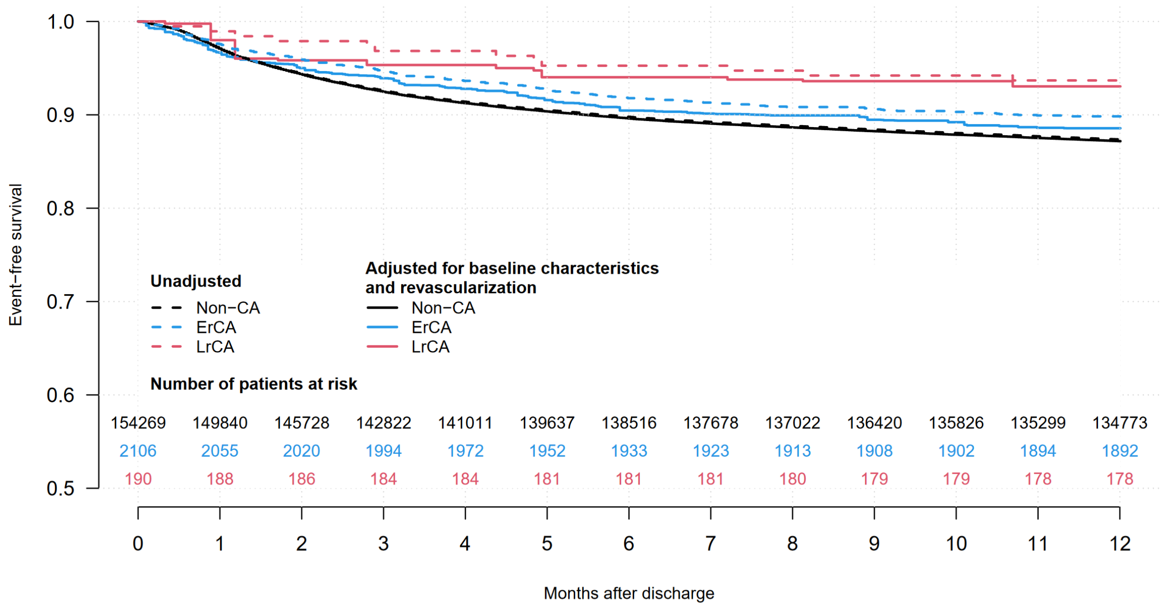

Adjusted for baseline characteristics, revascularization, and in–hospital complications

	1-year event-free (95% CI)	Hazard ratio (95% CI)	P value
**Non-CA**	0.872 (0.870 - 0.873)	1	
**rCA**	0.898 (0.880 - 0.916)	0.78 (0.65 – 0.95)	0.0131
**ErCA**	0.895 (0.875 - 0.915)	0.81 (0.66– 0.99)	0.0403
**LrCA**	0.929 (0.866 – 0.998)	0.54 (0.20– 1.43)	0.2134

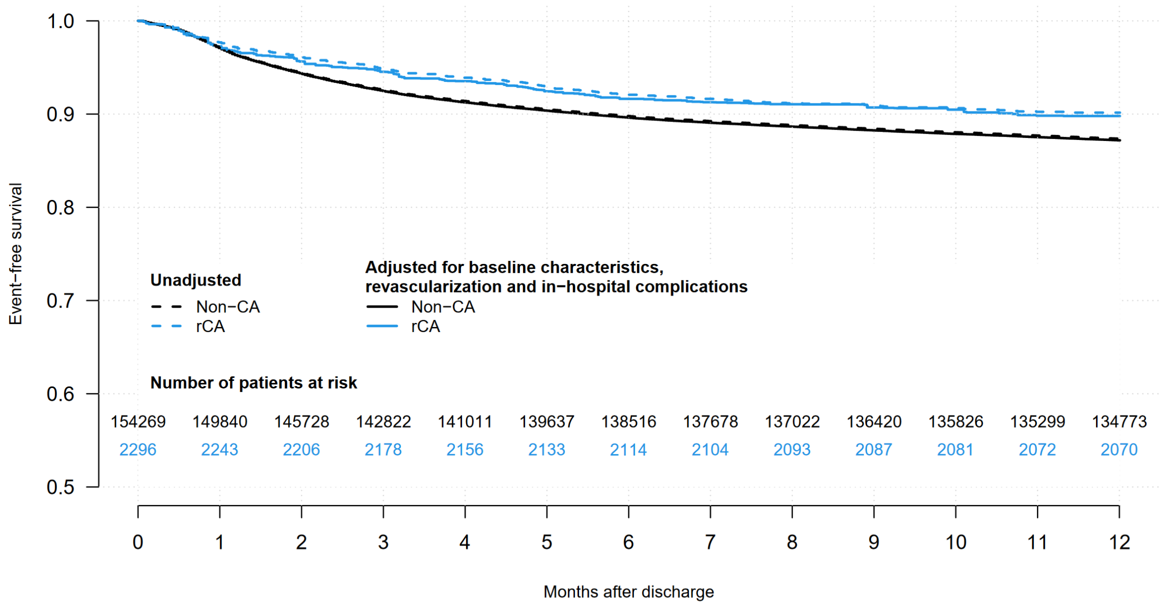

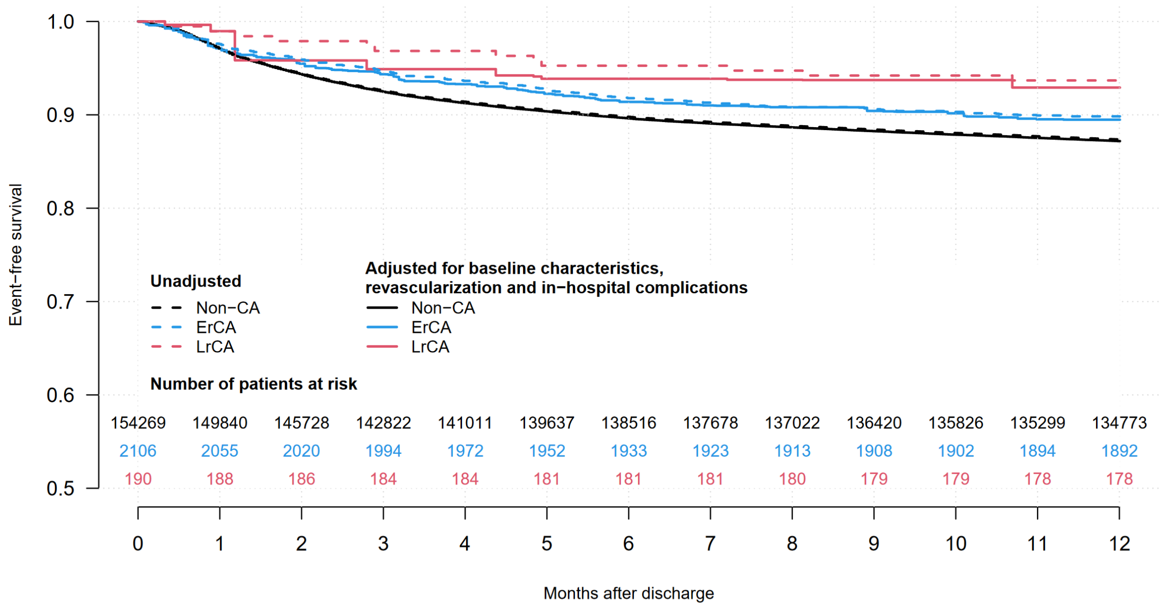

Adjusted for baseline characteristics, revascularization, in–hospital complications, and treatment procedures

	1-year event-free (95% CI)	Hazard ratio (95% CI)	P value
**Non-CA**	0.872 (0.870 – 0.874)	1	
**rCA**	0.896 (0.878 - 0.916)	0.80 (0.66 – 0.97)	0.0232
**ErCA**	0.893 (0.873 - 0.914)	0.82 (0.67 – 1.01)	0.0585
**LrCA**	0.919 (0.845 – 0.999)	0.62 (0.23 – 1.66)	0.3389

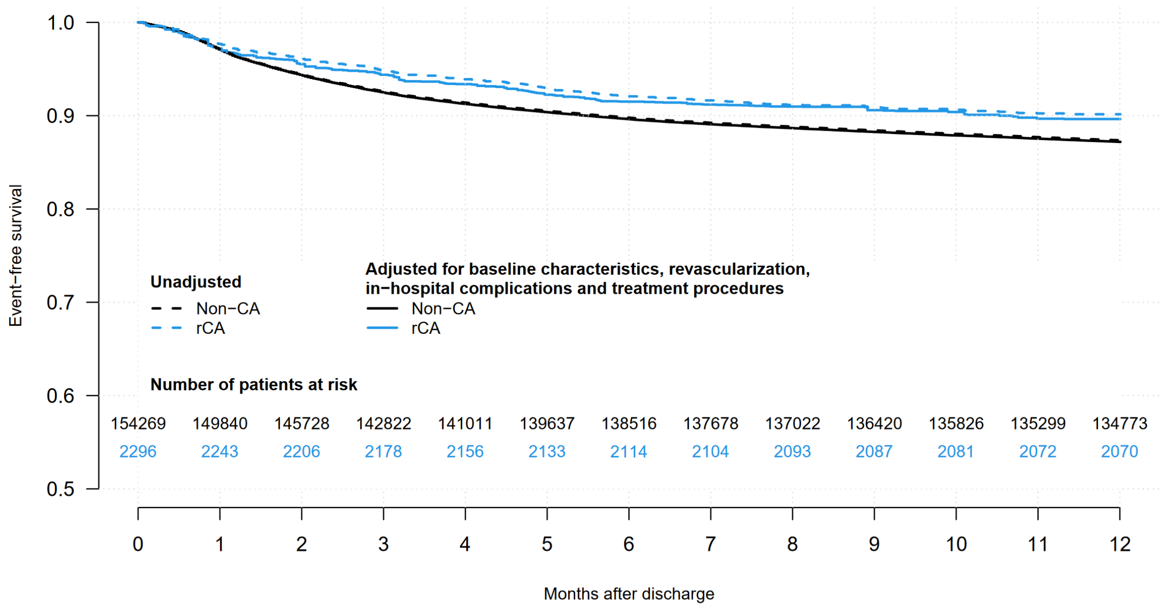

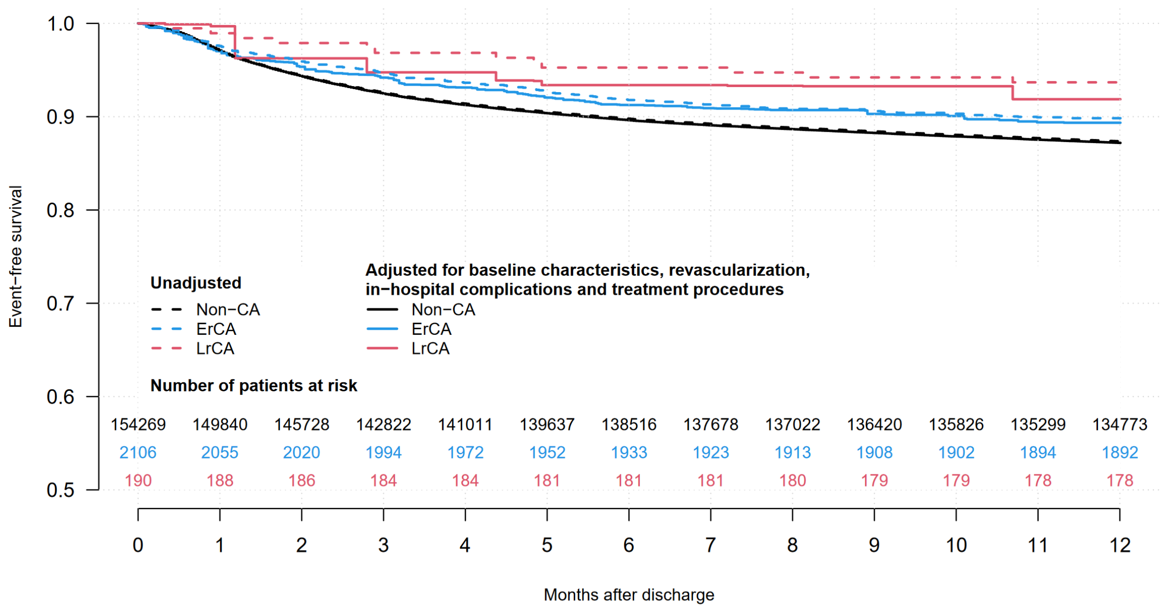

Relative risk of 1-year re-hospitalization due to unstable angina

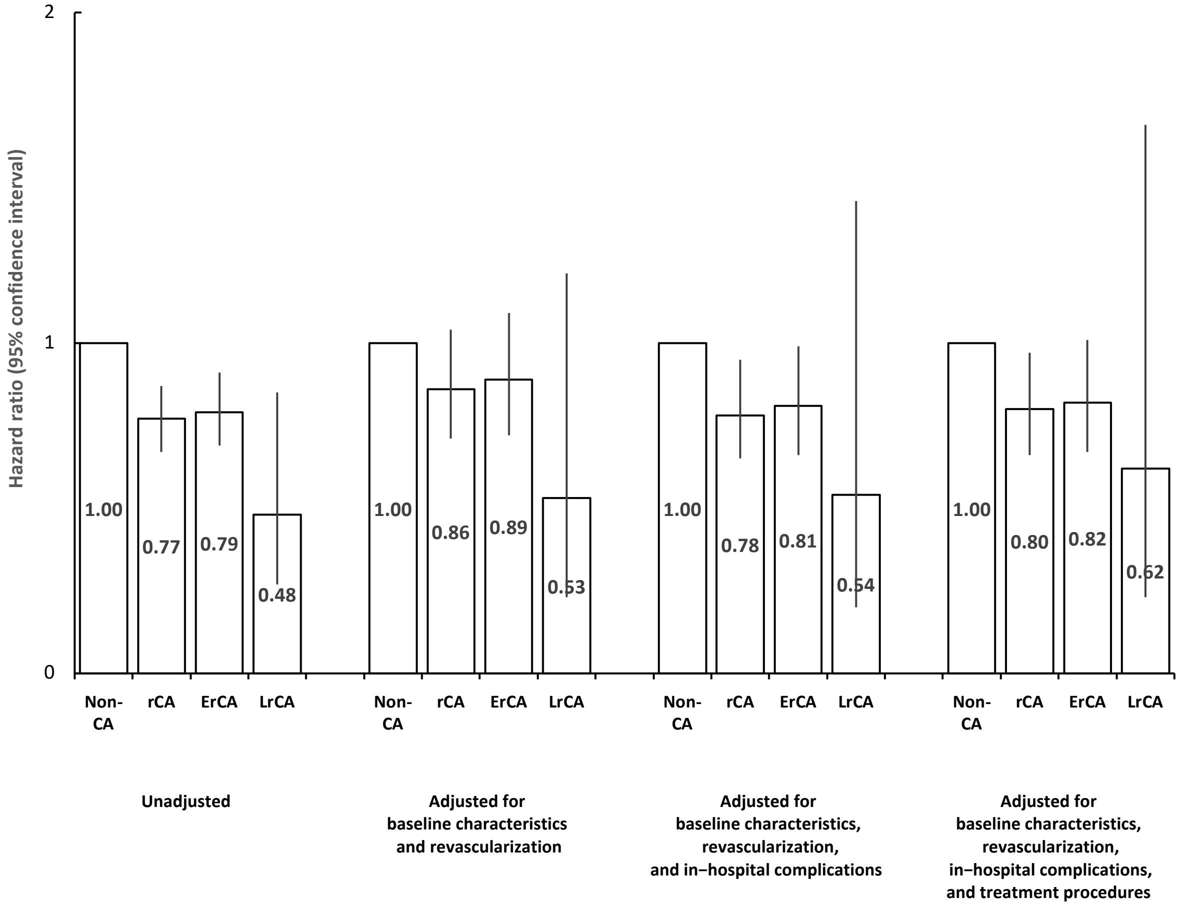

CA – cardiac arrest; rCA – resuscitated cardiac arrest; ErCA – early resuscitated cardiac arrest; LrCA – late resuscitated cardiac arrest

D) 1 year re-hospitalization due to chronic coronary syndrome

Unadjusted

	1-year event-free (95% CI)	Hazard ratio (95% CI)	P value
**Non-CA**	0.762 (0.759 - 0.764)	1	
**rCA**	0.756 (0.738 - 0.733)	1.02 (0.94 – 1.11)	0.577
**ErCA**	0.746 (0.728 - 0.765)	1.07 (0.98 – 1.16)	0.13029
**LrCA**	0.858 (0.810 – 0.909)	0.56 (0.39 – 0.82)	0.00286

CI – confidence interval; CA – cardiac arrest; rCA – resuscitated cardiac arrest; ErCA – early resuscitated cardiac arrest; LrCA – late resuscitated cardiac arrest

Adjusted for baseline characteristics and revascularization

	1-year event-free (95% CI)	Hazard ratio (95% CI)	P value
**Non-CA**	0.762 (0.760 - 0.764)	1	
**rCA**	0.770 (0.748 - 0.794)	0.96 (0.85 – 1.07)	0.443
**ErCA**	0.765 (0.740 - 0.790)	0.98 (0.87 – 1.11)	0.7919
**LrCA**	0.848 (0.778 – 0.924)	0.61 (0.36 – 1.04)	0.0668

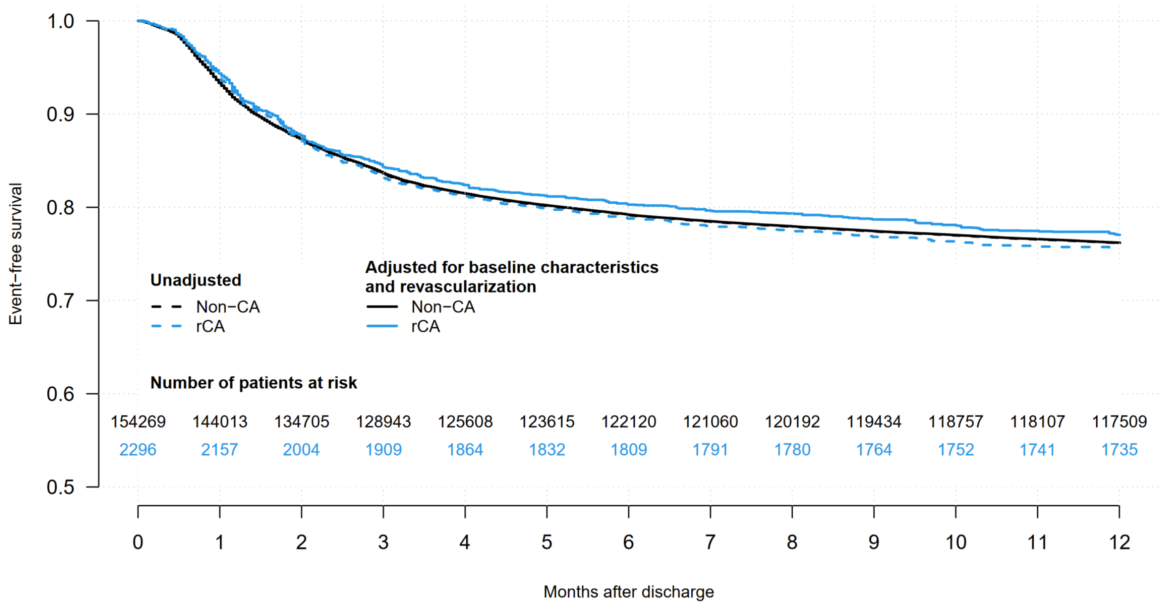

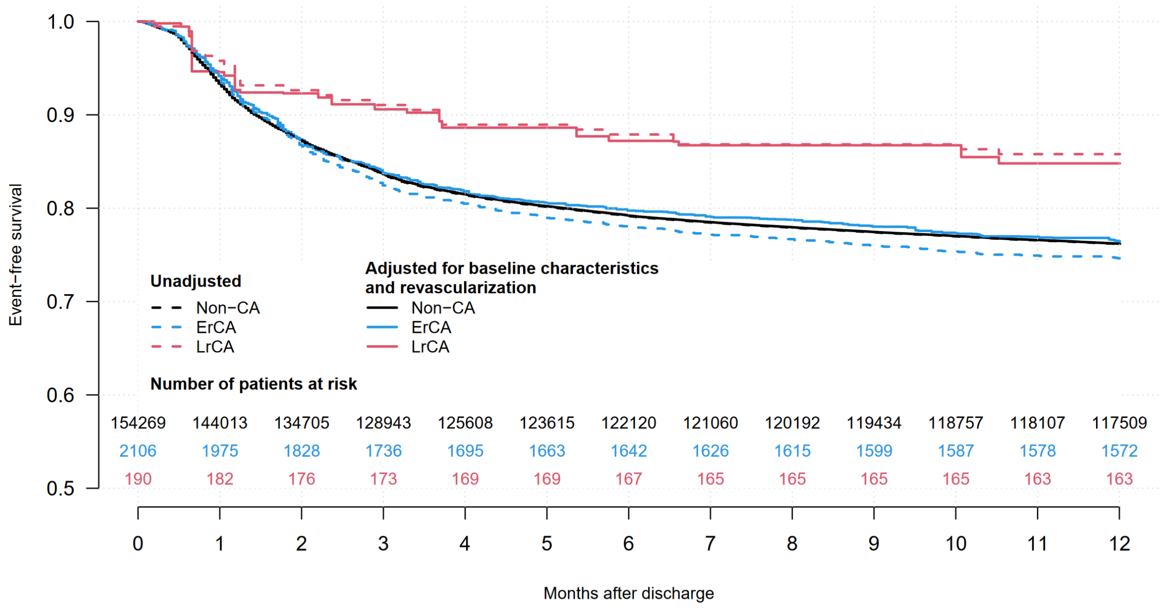

Adjusted for baseline characteristics, revascularization, and in–hospital complications

	1-year event-free (95% CI)	Hazard ratio (95% CI)	P value
**Non-CA**	0.762 (0.760 - 0.764)	1	
**rCA**	0.764 (0.740 - 0.789)	0.99 (0.88 – 1.11)	0.845
**ErCA**	0.758 (0.732 - 0.784)	1.02 (0.90 – 1.15)	0.7658
**LrCA**	0.872 (0.798 – 0.953)	0.51 (0.26 – 1.04)	0.0513

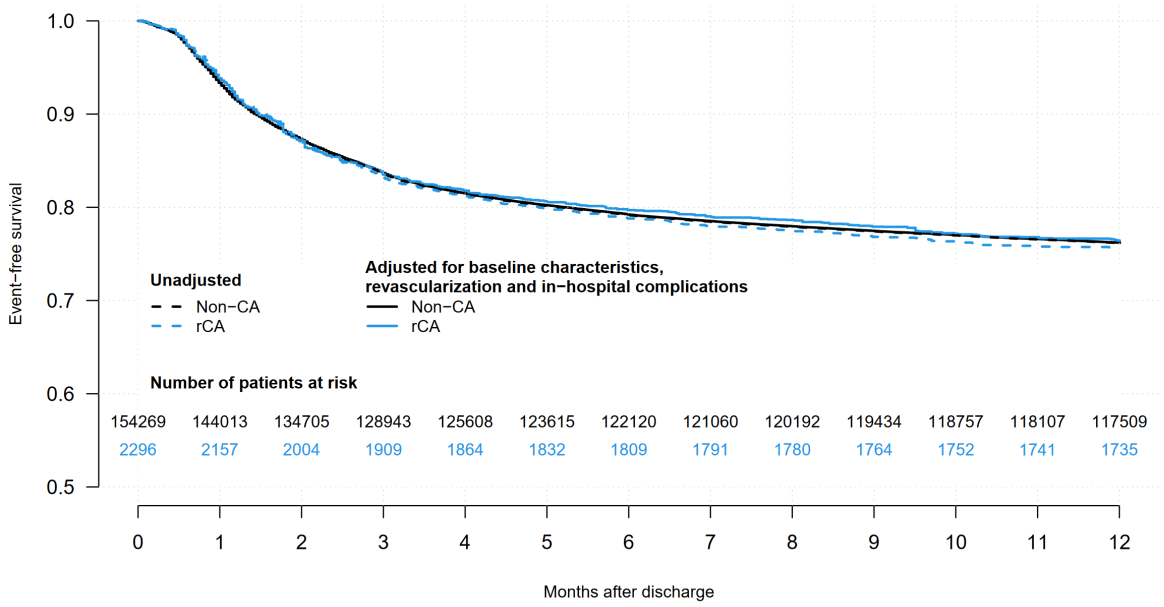

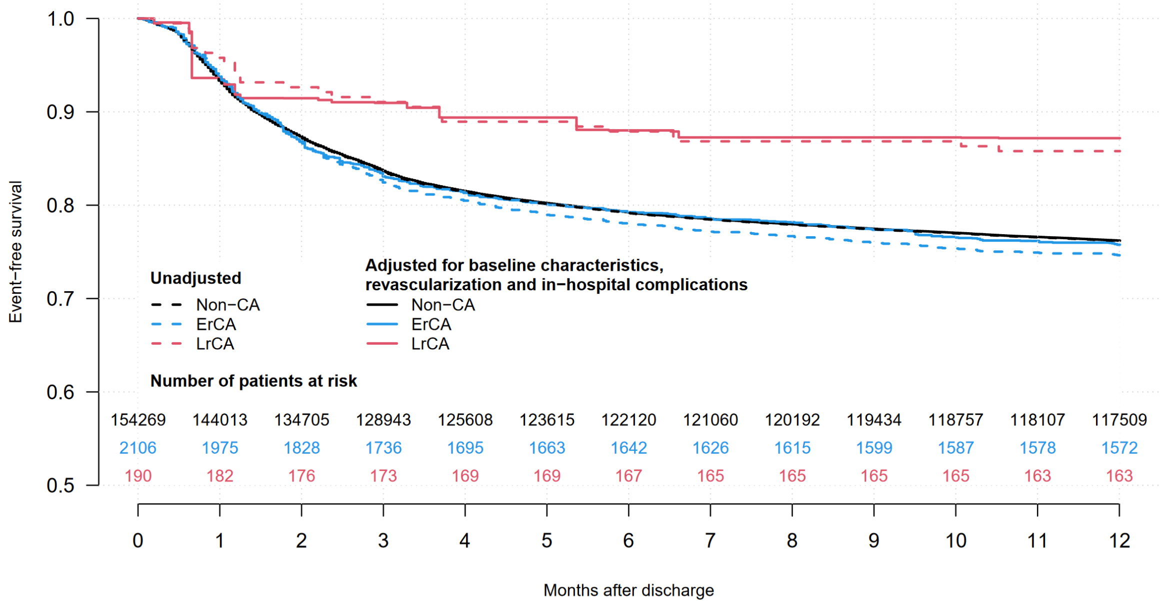

Adjusted for baseline characteristics, revascularization, in–hospital complications, and treatment procedures

	1-year event-free (95% CI)	Hazard ratio (95% CI)	P value
**Non-CA**	0.762 (0.760 - 0.764)	1	
**rCA**	0.757 (0.732 - 0.783)	1.02 (0.91 – 1.16)	0.702
**ErCA**	0.752 (0.726 - 0.779)	1.05 (0.92 – 1.18)	0.474
**LrCA**	0.854 (0.768 – 0.950)	0.59 (0.30 – 1.18)	0.138

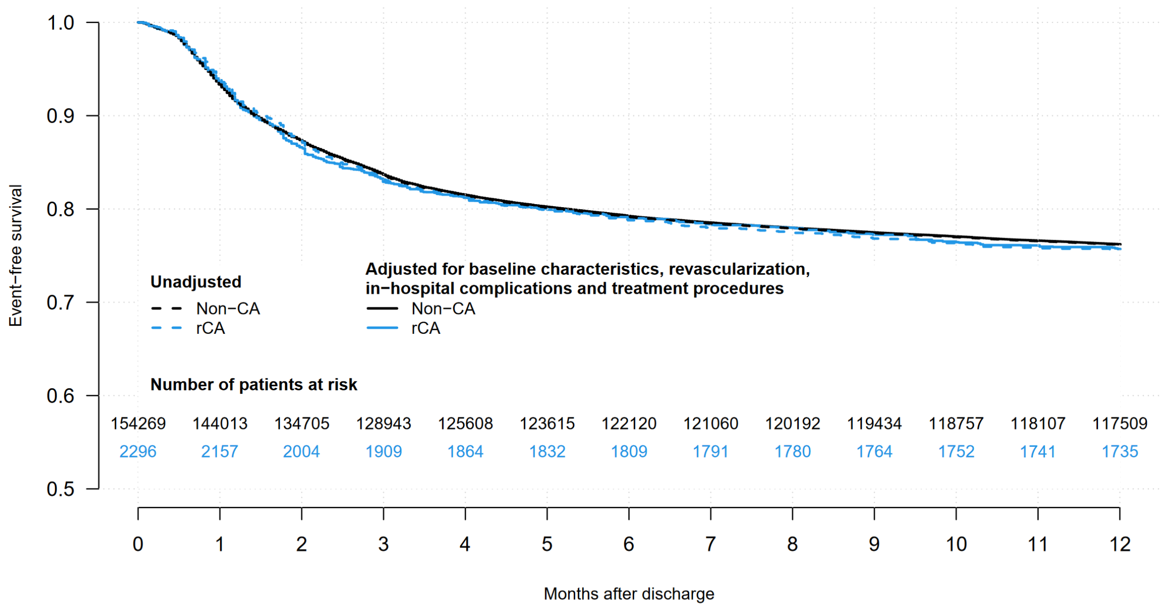

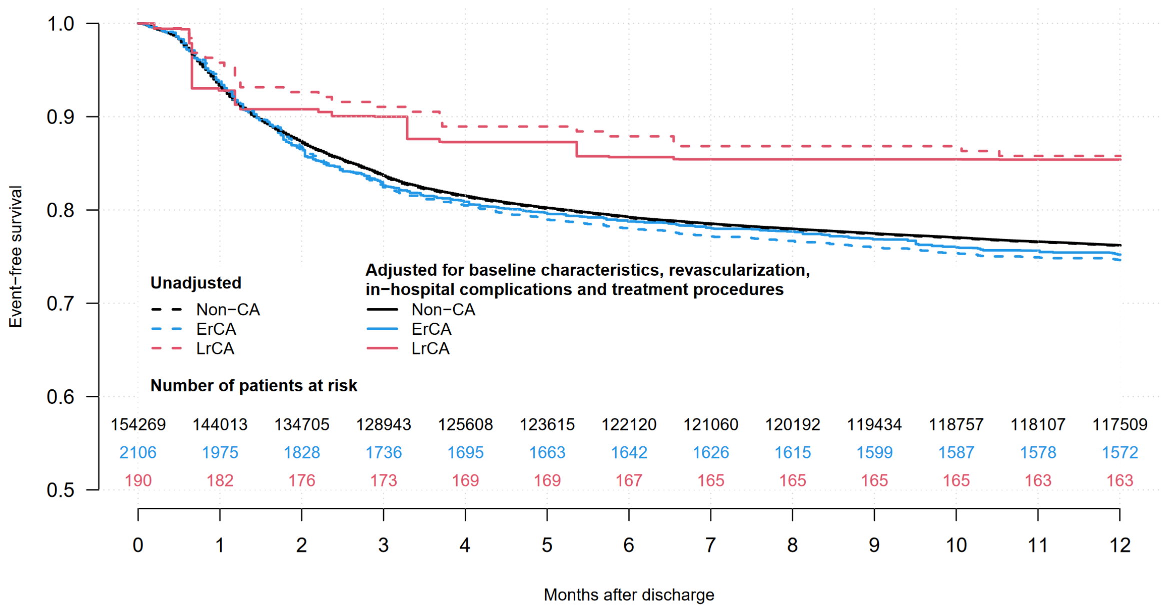

Relative risk of 1-year re-hospitalization due to chronic coronary syndrome

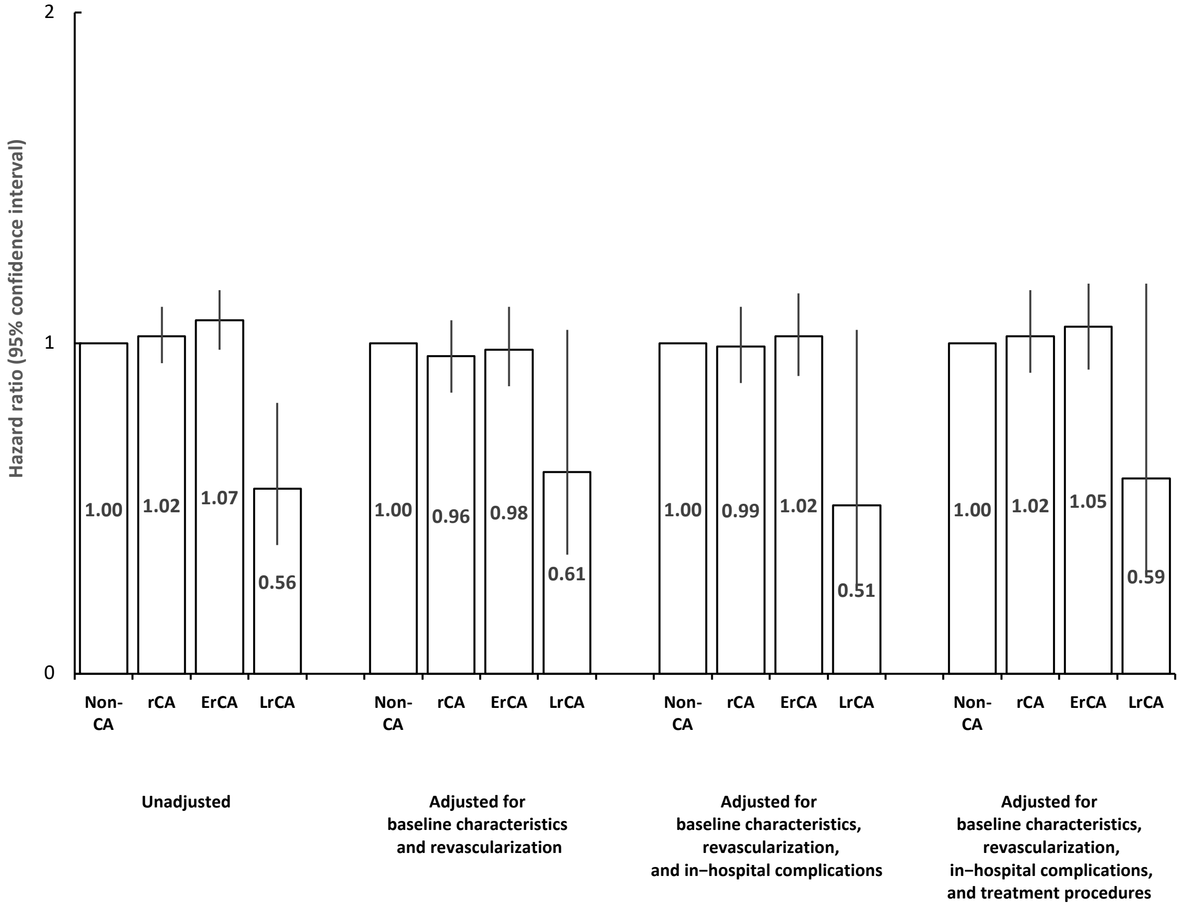

CA – cardiac arrest; rCA – resuscitated cardiac arrest; ErCA – early resuscitated cardiac arrest; LrCA – late resuscitated cardiac arrest

E) 1 year re-hospitalization due to ischemic heart disease (chronic coronary syndrome or unstable angina or acute myocardial infarction)

Unadjusted

	1-year event-free (95% CI)	Hazard ratio (95% CI)	P value
**Non-CA**	0.642 (0.640 - 0.644)	1	
**rCA**	0.664 (0.645 - 0.683)	0.92 (0.86 – 0.99)	0.023
**ErCA**	0.655 (0.635 - 0.676)	0.95 (0.88 – 1.02)	0.17235
**LrCA**	0.758 (0.699 – 0.821)	0.62 (0.46 – 0.83)	0.00115

CI – confidence interval; CA – cardiac arrest; rCA – resuscitated cardiac arrest; ErCA – early resuscitated cardiac arrest; LrCA – late resuscitated cardiac arrest

Adjusted for baseline characteristics and revascularization

	1-year event-free (95% CI)	Hazard ratio (95% CI)	P value
**Non-CA**	0.642 (0.639 - 0.644)	1	
**rCA**	0.662 (0.635 - 0.690)	0.93 (0.84 – 1.03)	0.14
**ErCA**	0.656 (0.627 - 0.686)	0.95 (0.86 – 1.06)	0.3431
**LrCA**	0.736 (0.649 – 0.835)	0.69 (0.45 – 1.05)	0.0816

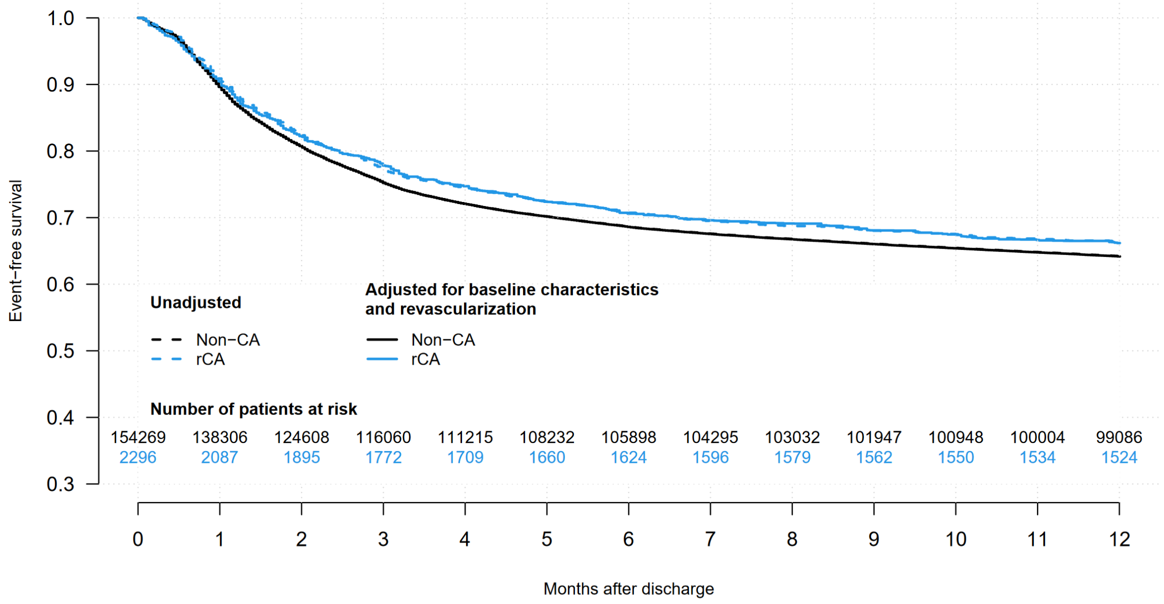

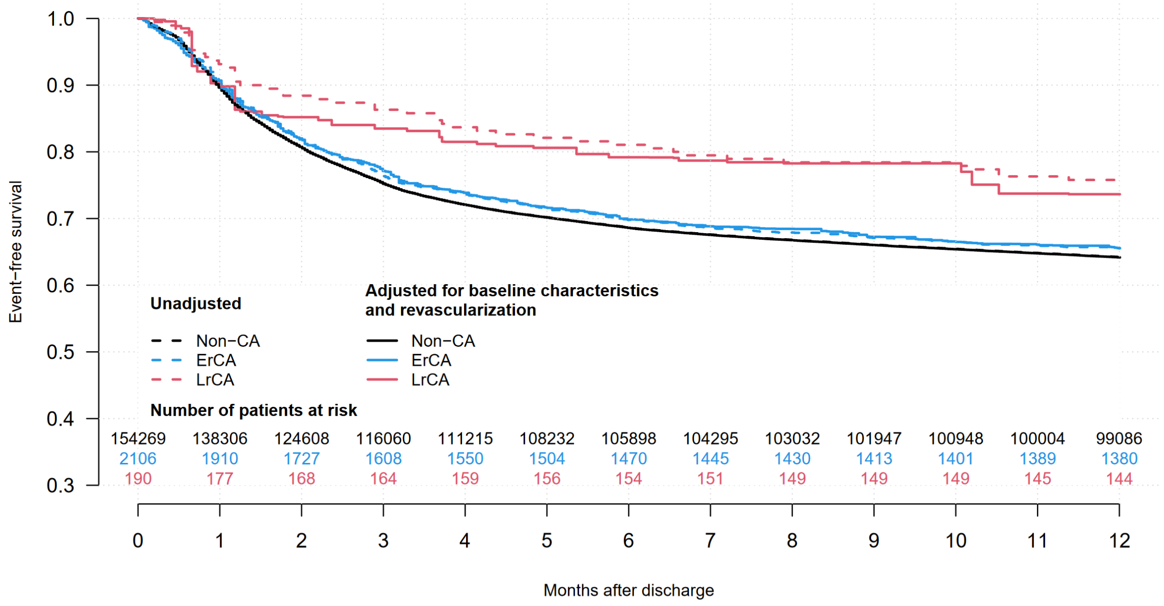

Adjusted for baseline characteristics, revascularization, and in−hospital complications

	1-year event-free (95% CI)	Hazard ratio (95% CI)	P value
**Non-CA**	0.642 (0.639 - 0.644)	1	
**rCA**	0.668 (0.640 - 0.696)	0.91 (0.82 – 1.01)	0.0789
**ErCA**	0.659 (0.631 - 0.689)	0.94 (0.85 – 1.05)	0.2556
**LrCA**	0.763 (0.666 – 0.874)	0.62 (0.37 – 1.03)	0.0672

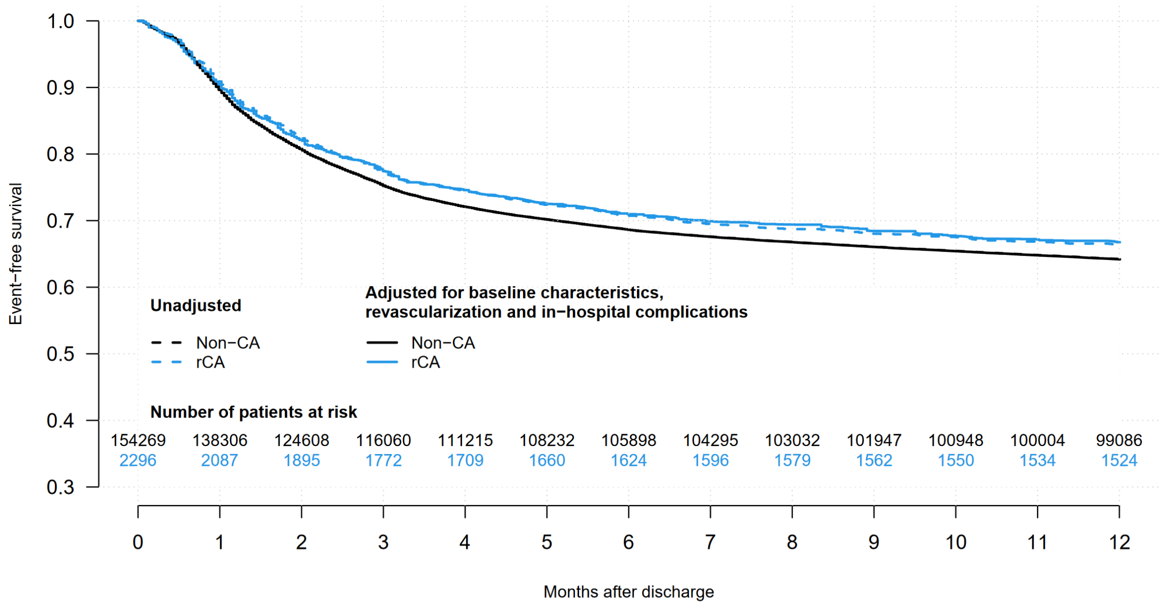

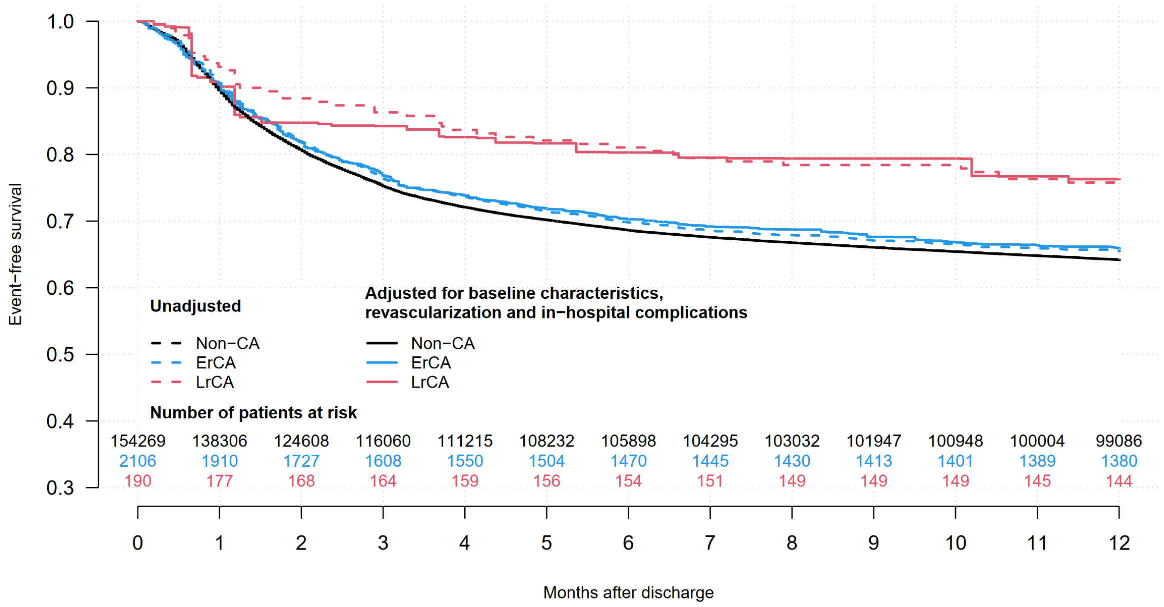

Adjusted for baseline characteristics, revascularization, in–hospital complications, and treatment procedures

	1-year event-free (95% CI)	Hazard ratio (95% CI)	P value
**Non-CA**	0.642 (0.640 - 0.644)	1	
**rCA**	0.660 (0.632 - 0.689)	0.94 (0.85 – 1.04)	0.25
**ErCA**	0.653 (0.624 - 0.684)	0.96 (0.86 – 1.07)	0.488
**LrCA**	0.731 (0.621 – 0.860)	0.71 (0.42 – 1.20)	0.206

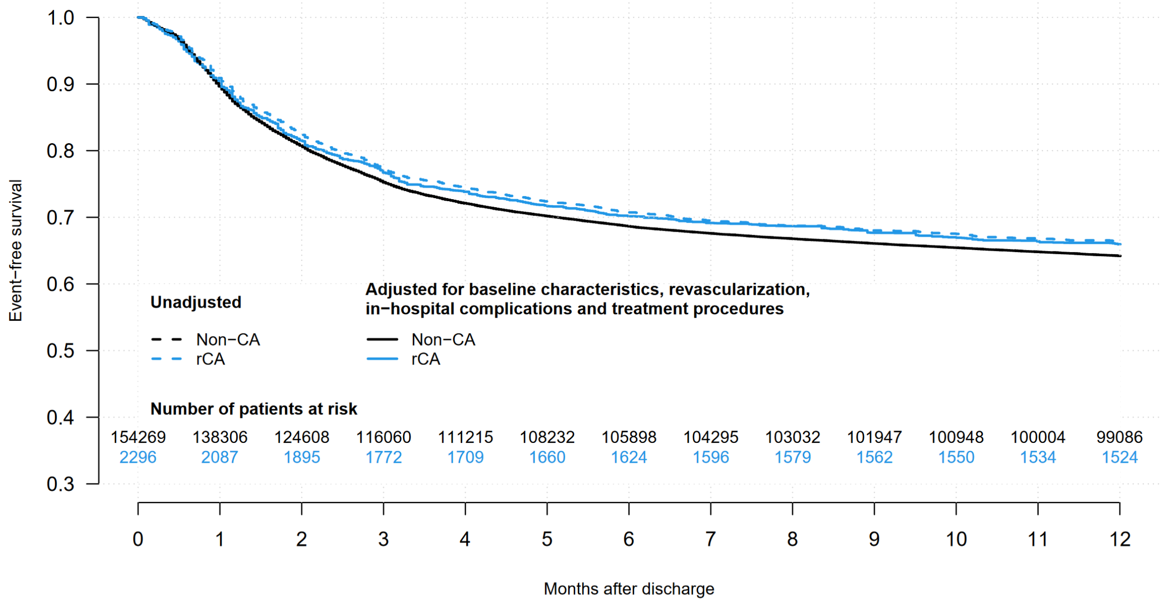

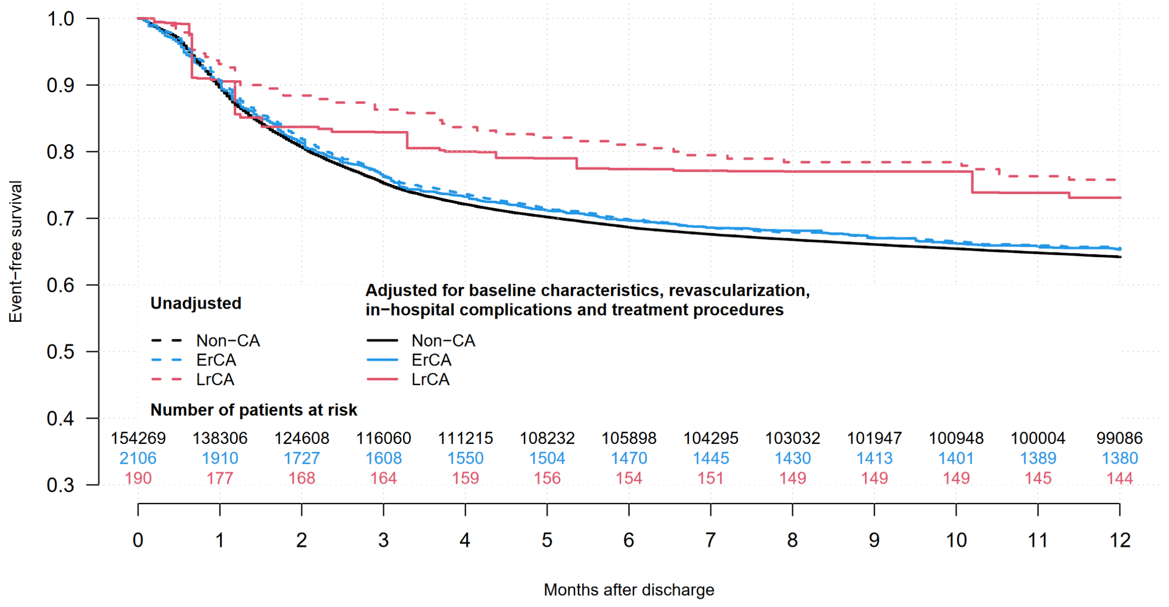

Relative risk of 1-year re-hospitalization due to ischemic heart disease

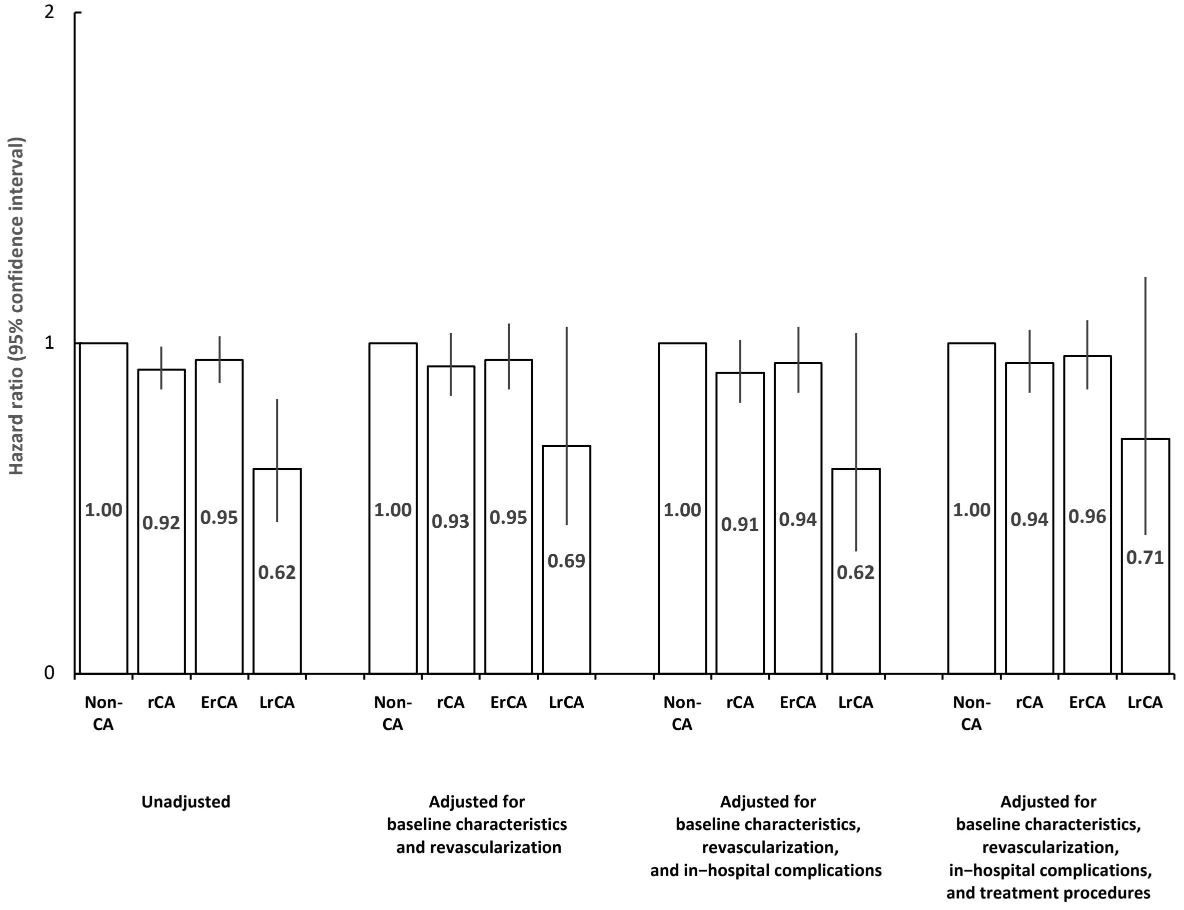

CA – cardiac arrest; rCA – resuscitated cardiac arrest; ErCA – early resuscitated cardiac arrest; LrCA – late resuscitated cardiac arrest

F) 1 year re-hospitalization due to heart failure

Unadjusted

	1-year event-free (95% CI)	Hazard ratio (95% CI)	P value
**Non-CA**	0.918 (0.917 - 0.919)	1	
**rCA**	0.885 (0.873 - 0.899)	1.43 (1.27 – 1.62)	<0.0001
**ErCA**	0.819 (0.878 - 0.905)	1.36 (1.19 – 1.55)	<0.0001
**LrCA**	0.821 (0.768 – 0.877)	2.34 (1.67– 3.27)	<0.0001

CI – confidence interval; CA – cardiac arrest; rCA – resuscitated cardiac arrest; ErCA – early resuscitated cardiac arrest; LrCA – late resuscitated cardiac arrest

Adjusted for baseline characteristics and revascularization

	1-year event-free (95% CI)	Hazard ratio (95% CI)	P value
**Non-CA**	0.918 (0.916 - 0.919)	1	
**rCA**	0.898 (0.881 - 0.916)	1.26 (1.05– 1.51)	0.0153
**ErCA**	0.898 (0.879 - 0.917)	1.26 (1.04 – 1.54)	0.0211
**LrCA**	0.903 (0.855 – 0.955)	1.20 (0.69 – 2.09)	0.5234

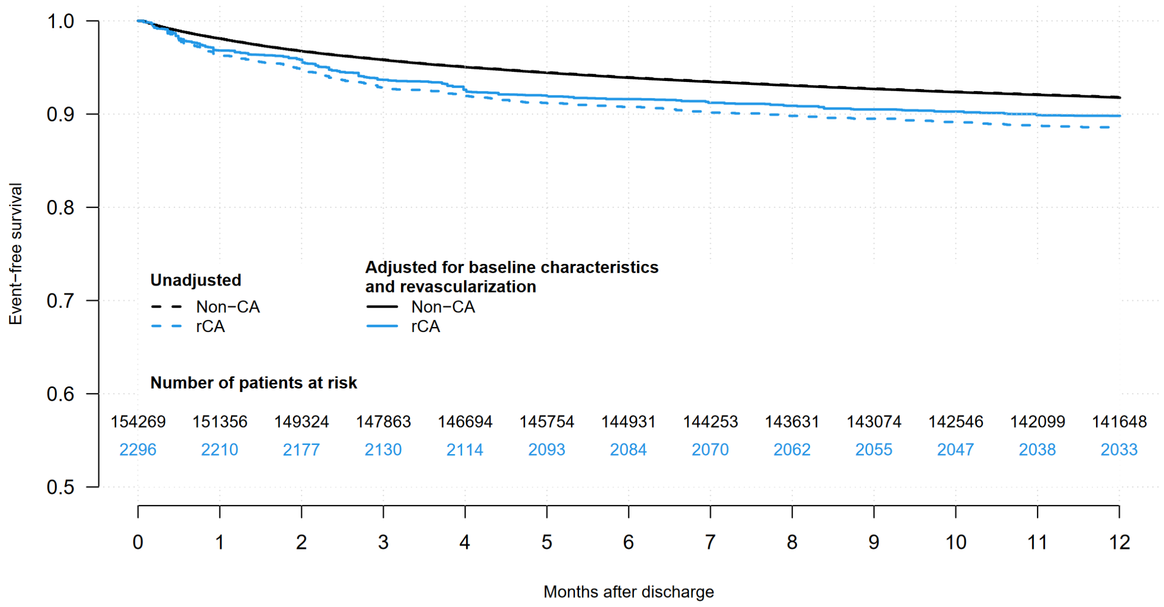

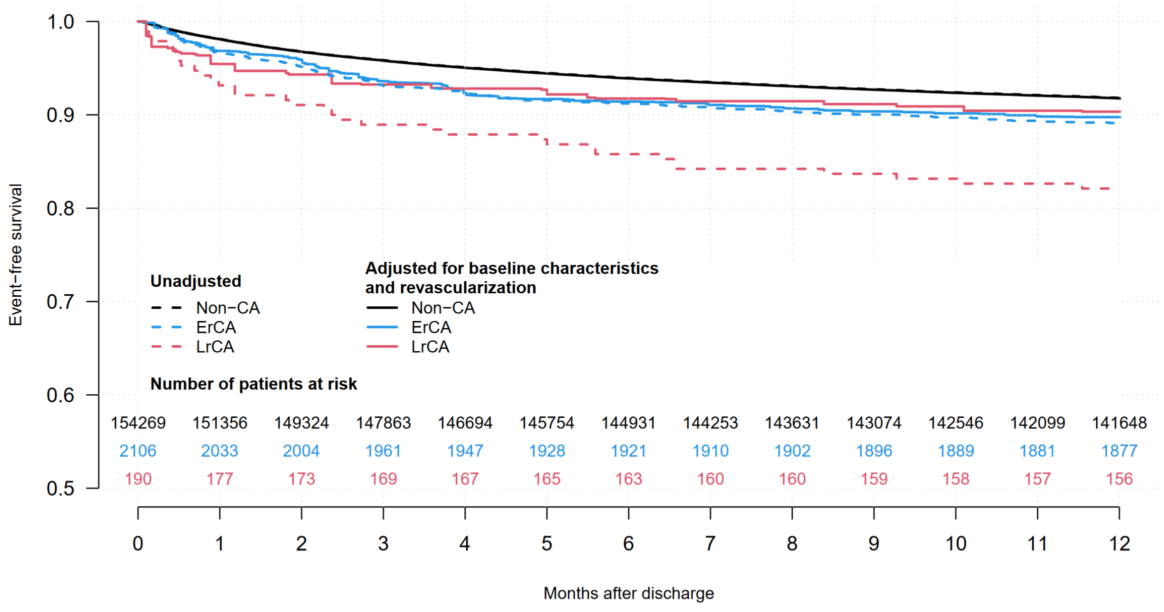

Adjusted for baseline characteristics, revascularization, and in–hospital complications

	1-year event-free (95% CI)	Hazard ratio (95% CI)	P value
**Non-CA**	0.917 (0.916 - 0.919)	1	
**rCA**	0.899 (0.882 - 0.917)	1.24 (1.03 – 1.49)	0.0232
**ErCA**	0.898 (0.879 - 0.917)	1.25 (1.03– 1.52)	0.0272
**LrCA**	0.877 (0.813 – 0.946)	1.54 (0.86– 2.77)	0.1506

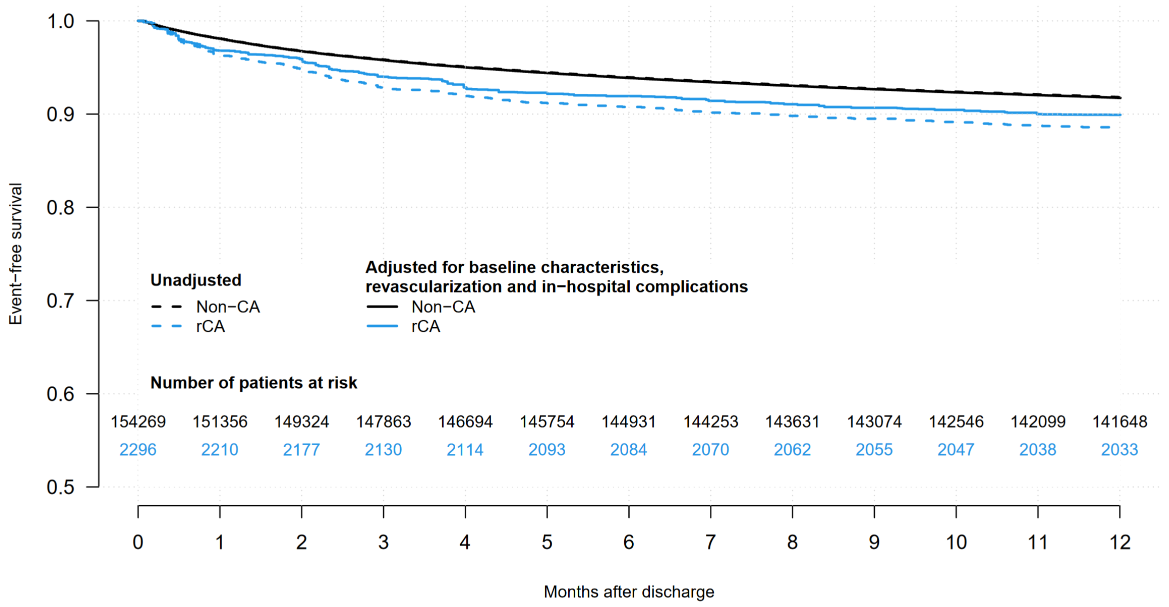

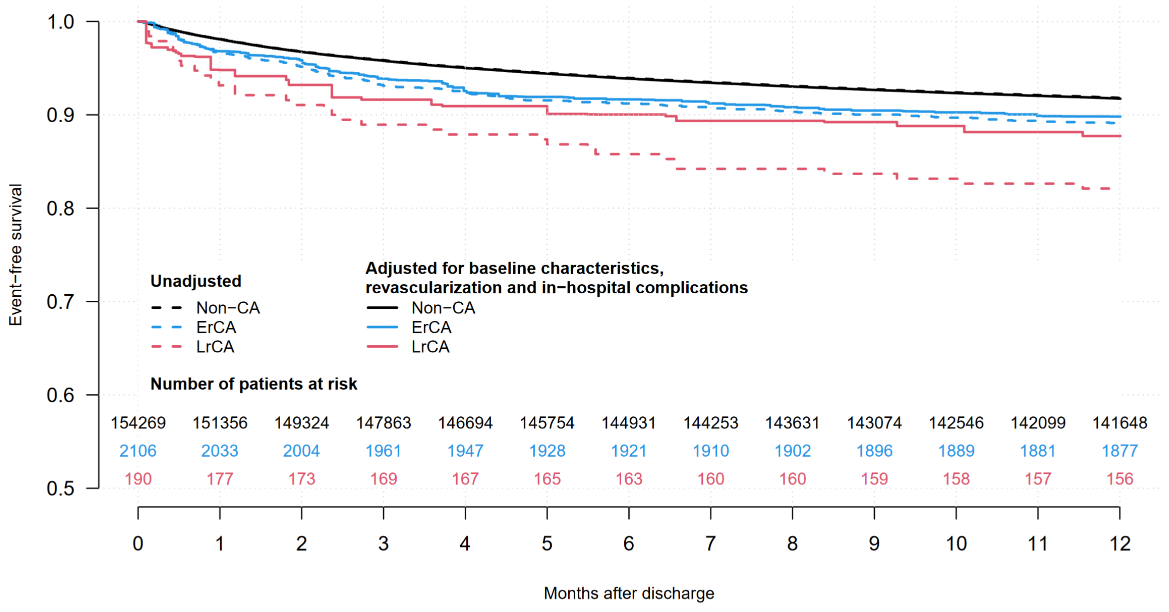

Adjusted for baseline characteristics, revascularization, in–hospital complications, and treatment procedures

	1-year event-free (95% CI)	Hazard ratio (95% CI)	P value
**Non-CA**	0.917 (0.916 – 0.918)	1	
**rCA**	0.902 (0.884 - 0.920)	1.20 (0.99 – 1.46)	0.0608
**ErCA**	0.900 (0.881 - 0.920)	1.22 (0.99 – 1.49)	0.0607
**LrCA**	0.881 (0.820 – 0.947)	1.47 (0.83 – 2.61)	0.1839

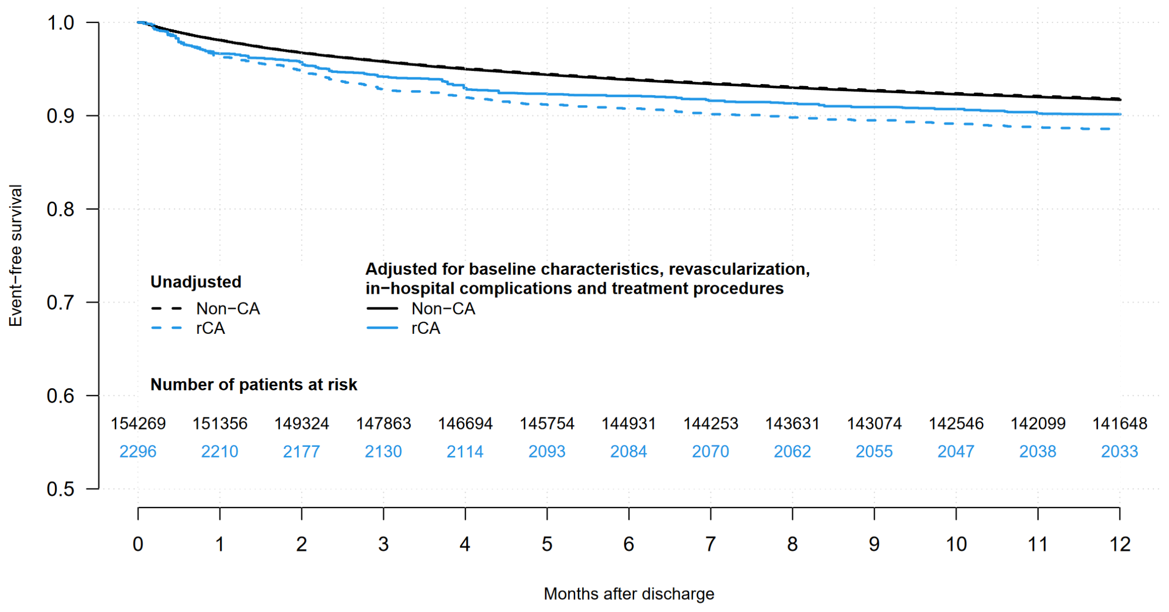

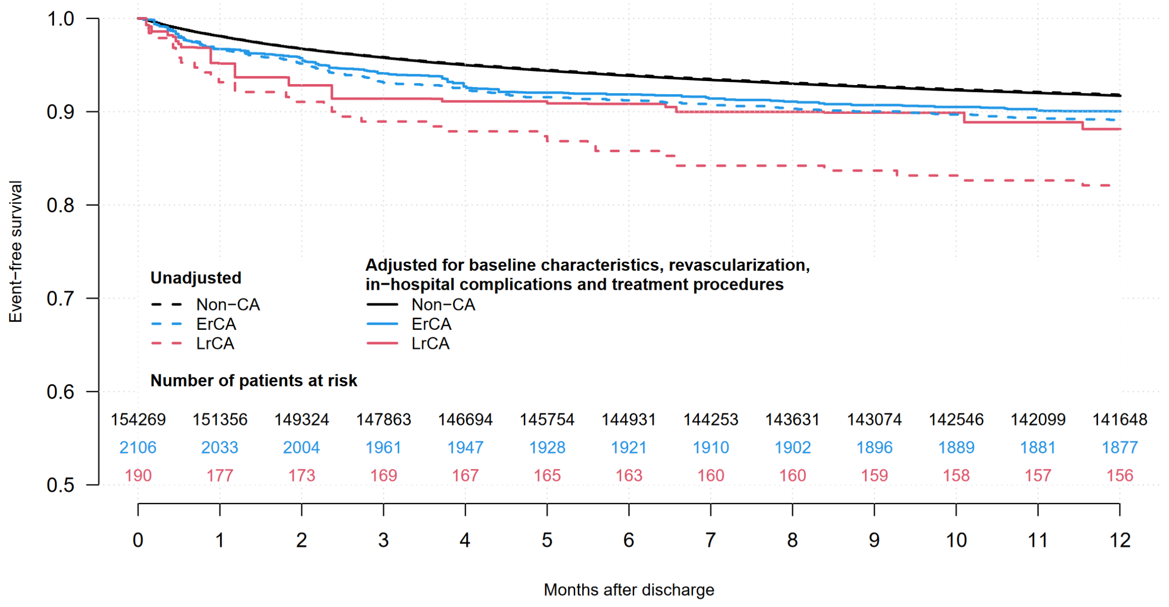

Relative risk of 1-year re-hospitalization due to heart failure

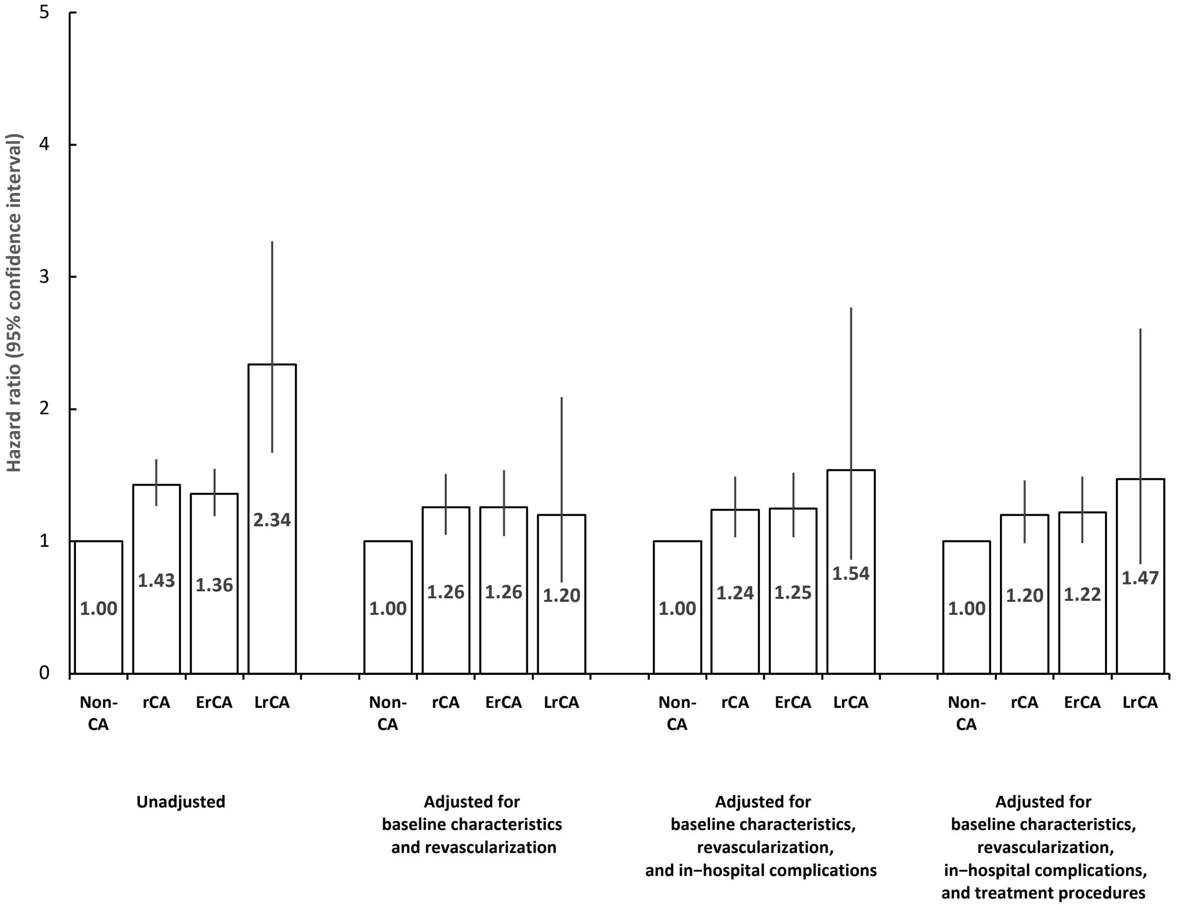

CA – cardiac arrest; rCA – resuscitated cardiac arrest; ErCA – early resuscitated cardiac arrest; LrCA – late resuscitated cardiac arrest

G) 1 year re-hospitalization due to cardiovascular reason

Unadjusted

	1-year event-free (95% CI)	Hazard ratio (95% CI)	P value
**Non-CA**	0.536 (0.533 - 0.538)	1	
**rCA**	0.521 (0.501 - 0.542)	1.05 (0.99 – 1.11)	0.121
**ErCA**	0.523 (0.502 - 0.545)	1.04 (0.98 – 1.10)	0.201
**LrCA**	0.500 (0.434 – 0.576)	1.13 (0.92 – 1.38)	0.246

CI – confidence interval; CA – cardiac arrest; rCA – resuscitated cardiac arrest; ErCA – early resuscitated cardiac arrest; LrCA – late resuscitated cardiac arrest

Adjusted for baseline characteristics and revascularization

	1-year event-free (95% CI)	Hazard ratio (95% CI)	P value
Non-CA	0.535 (0.533 - 0.538)	1	
rCA	0.526 (0.498 - 0.555)	1.03 (0.95 – 1.12)	0.455
ErCA	0.525 (0.495 - 0.556)	1.04 (0.95 – 1.13)	0.431
LrCA	0.533 (0.436 – 0.650)	1.05 (0.76 – 1.47)	0.753

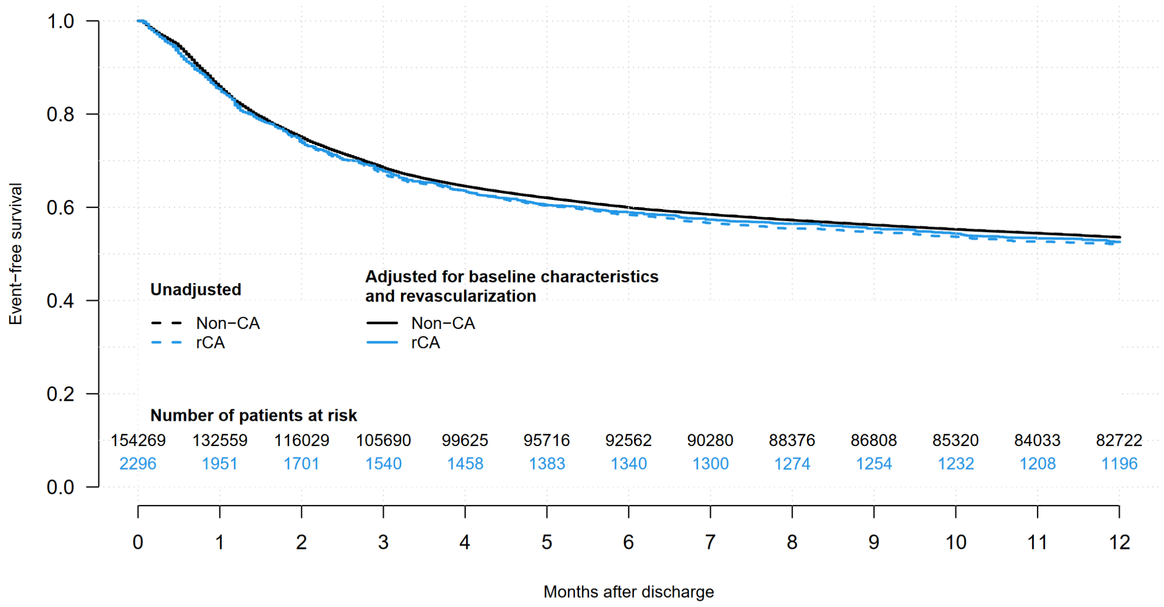

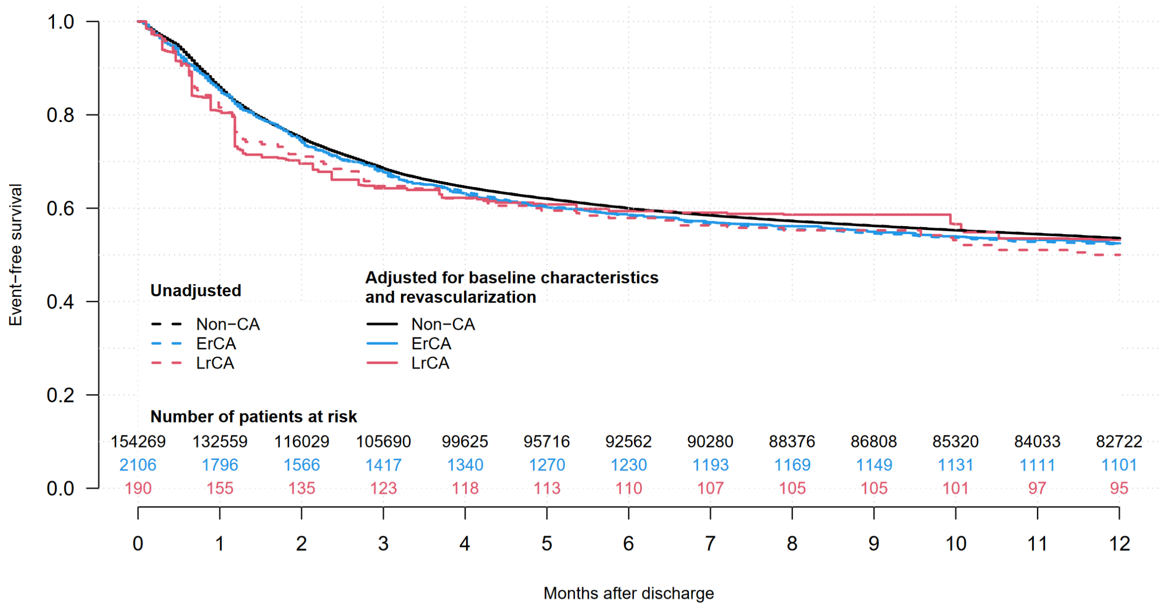

Adjusted for baseline characteristics, revascularization, and in–hospital complications

	1-year event-free (95% CI)	Hazard ratio (95% CI)	P value
**Non-CA**	0.535 (0.533 - 0.538)	1	
**rCA**	0.528 (0.499 - 0.558)	1.03 (0.94 – 1.12)	0.512
**ErCA**	0.526 (0.496 - 0.558)	1.03 (0.94 – 1.13)	0.481
**LrCA**	0.495 (0.390 – 0.627)	1.20 (0.84 – 1.72)	0.309

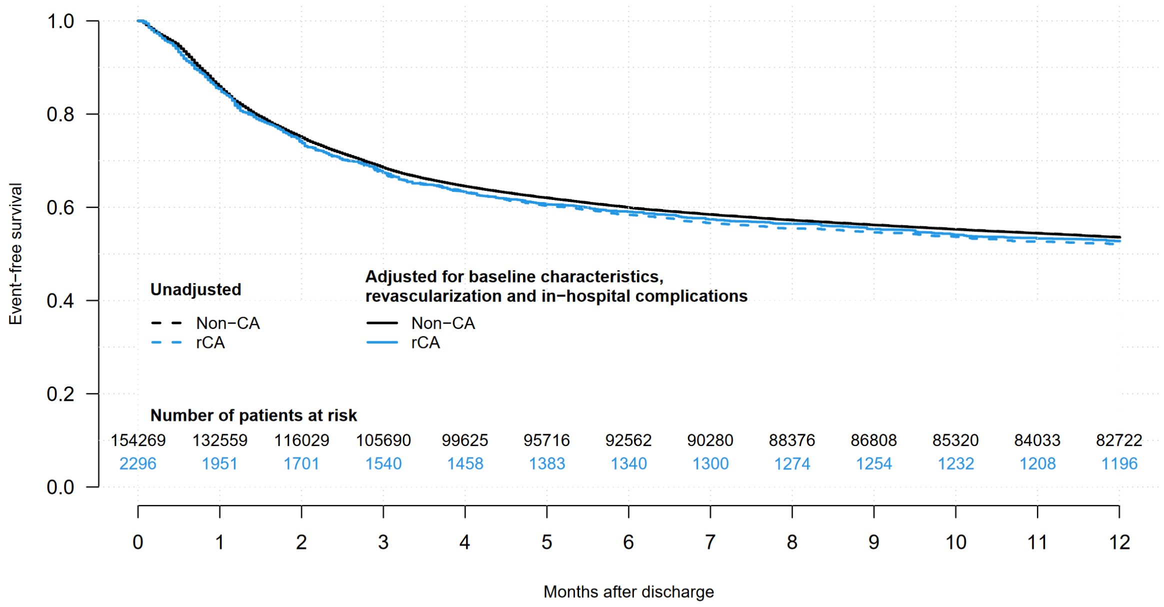

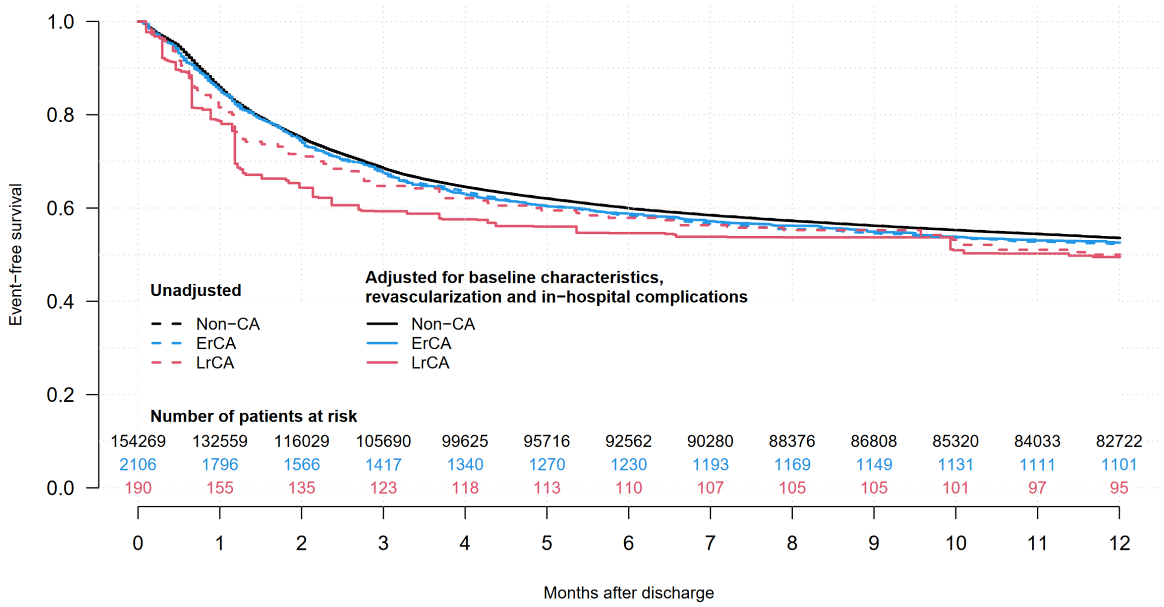

Adjusted for baseline characteristics, revascularization, in–hospital complications, and treatment procedures

	1-year event-free (95% CI)	Hazard ratio (95% CI)	P value
**Non-CA**	0.535 (0.533 - 0.538)	1	
**rCA**	0.519 (0.490 - 0.550)	1.06 (0.97 – 1.16)	0.226
**ErCA**	0.519 (0.489 - 0.552)	1.05 (0.96 – 1.15)	0.255
**LrCA**	0.451 (0.341 – 0.597)	1.33 (0.93 – 1.91)	0.116

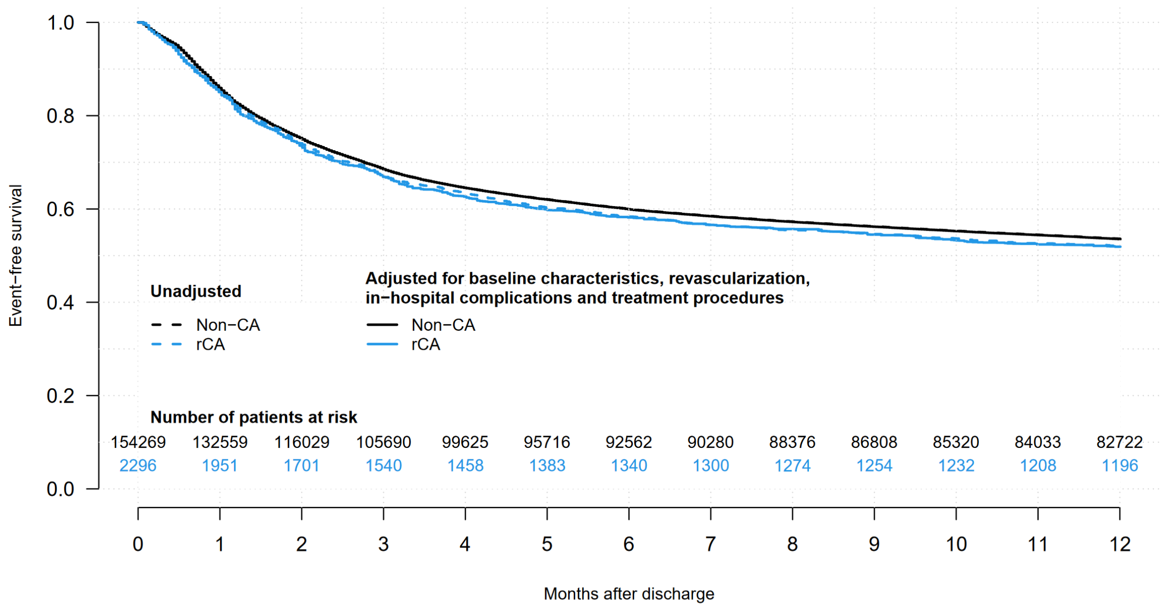

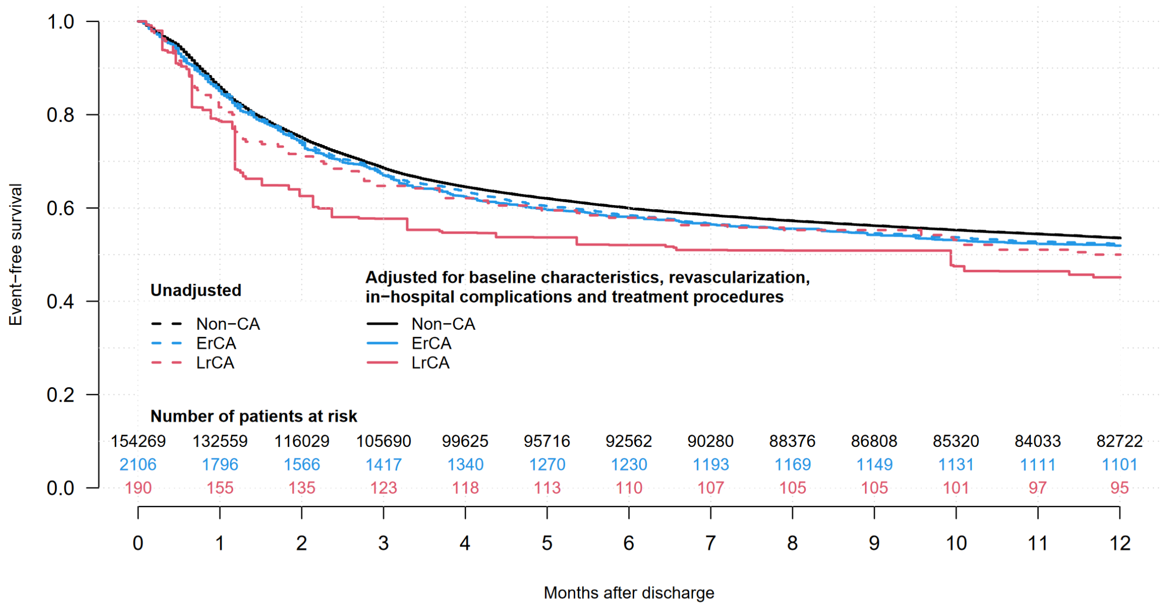

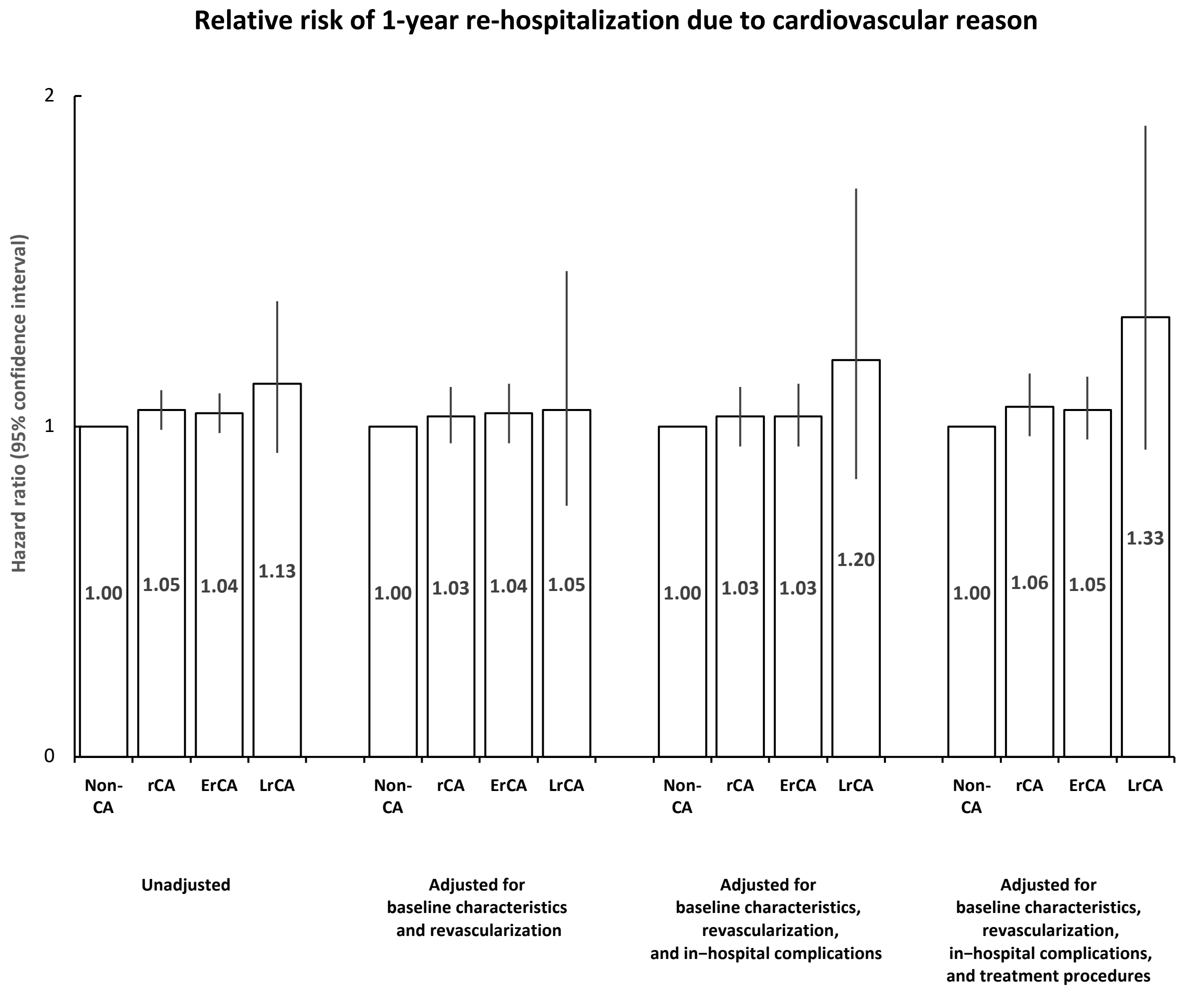

CA – cardiac arrest; rCA – resuscitated cardiac arrest; ErCA – early resuscitated cardiac arrest; LrCA – late resuscitated cardiac arrest

H) 1 year re-hospitalization due to any reason

Unadjusted

	1-year event-free (95% CI)	Hazard ratio (95% CI)	P value
**Non-CA**	0.427 (0.424 - 0.429)	1	
**rCA**	0.409 (0.389 - 0.430)	1.06 (1.00 – 1.11)	0.0452
**ErCA**	0.415 (0.394 - 0.436)	1.04 (0.98 – 1.10)	0.22958
**LrCA**	0.347 (0.286 – 0.422)	1.33 (1.11 – 1.58)	0.00162

CI – confidence interval; CA – cardiac arrest; rCA – resuscitated cardiac arrest; ErCA – early resuscitated cardiac arrest; LrCA – late resuscitated cardiac arrest

Adjusted for baseline characteristics and revascularization

	1-year event-free (95% CI)	Hazard ratio (95% CI)	P value
**Non-CA**	0.426 (0.424 - 0.429)	1	
**rCA**	0.427 (0.399 - 0.456)	1.01 (0.94 – 1.10)	0.755
**ErCA**	0.430 (0.401 - 0.461)	1.00 (0.92 – 1.08)	0.986
**LrCA**	0.389 (0.298 – 0.509)	1.19 (0.89 – 1.60)	0.235

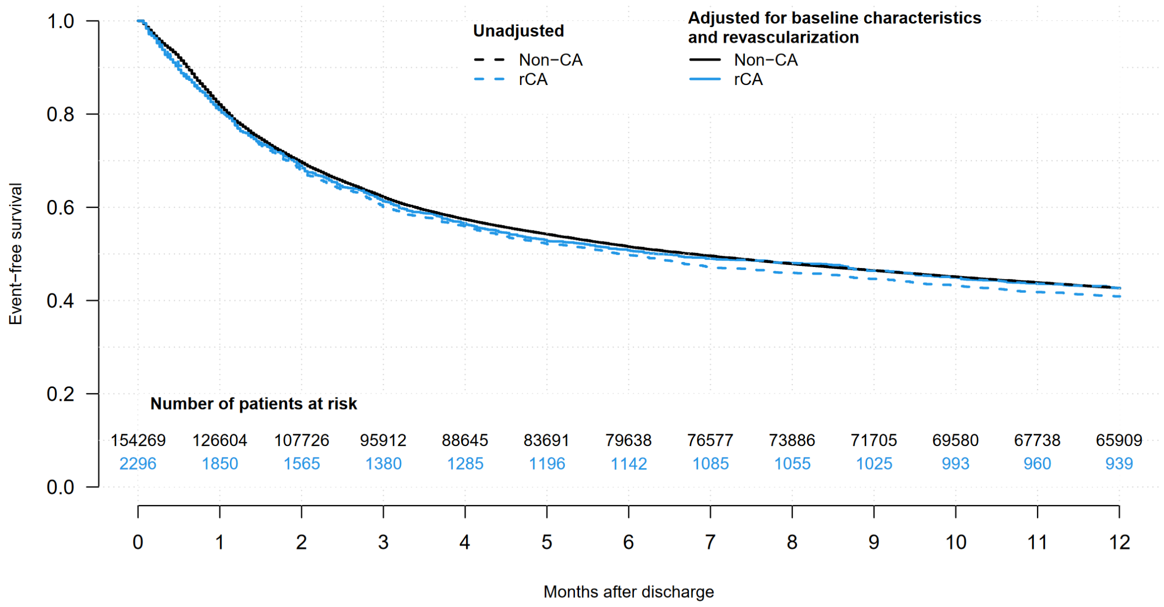

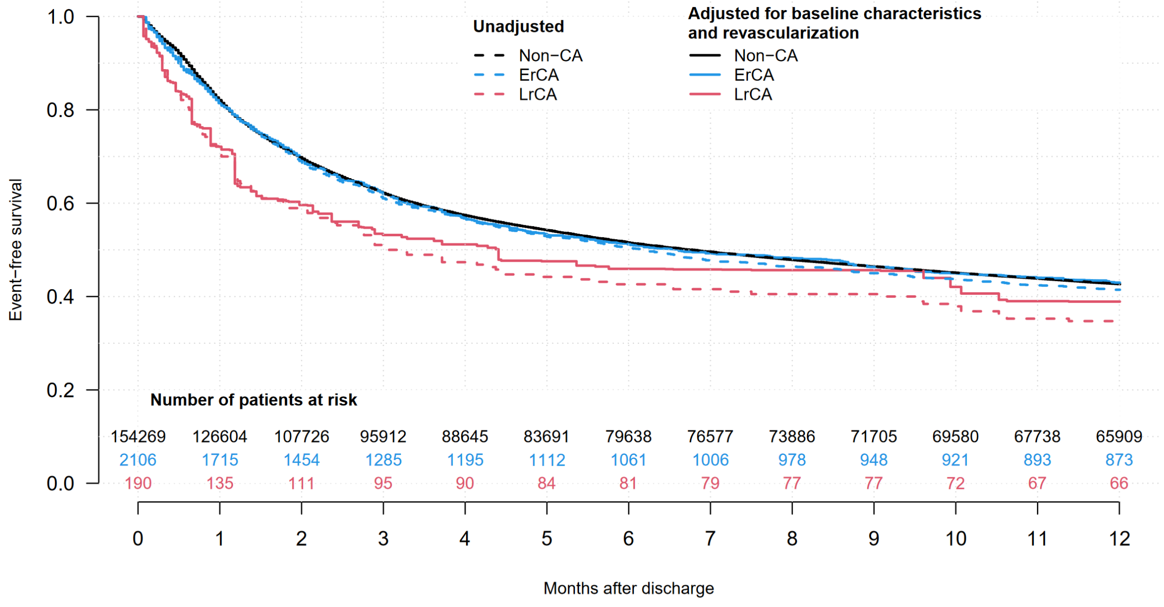

Adjusted for baseline characteristics, revascularization, and in–hospital complications

	1-year event-free (95% CI)	Hazard ratio (95% CI)	P value
**Non-CA**	0.426 (0.424 - 0.429)	1	
**rCA**	0.428 (0.400 - 0.459)	1.00 (0.93 – 1.09)	0.912
**ErCA**	0.431 (0.401 - 0.463)	0.99 (0.92 – 1.08)	0.8825
**LrCA**	0.338 (0.249 – 0.457)	1.37 (1.03 – 1.82)	0.0322

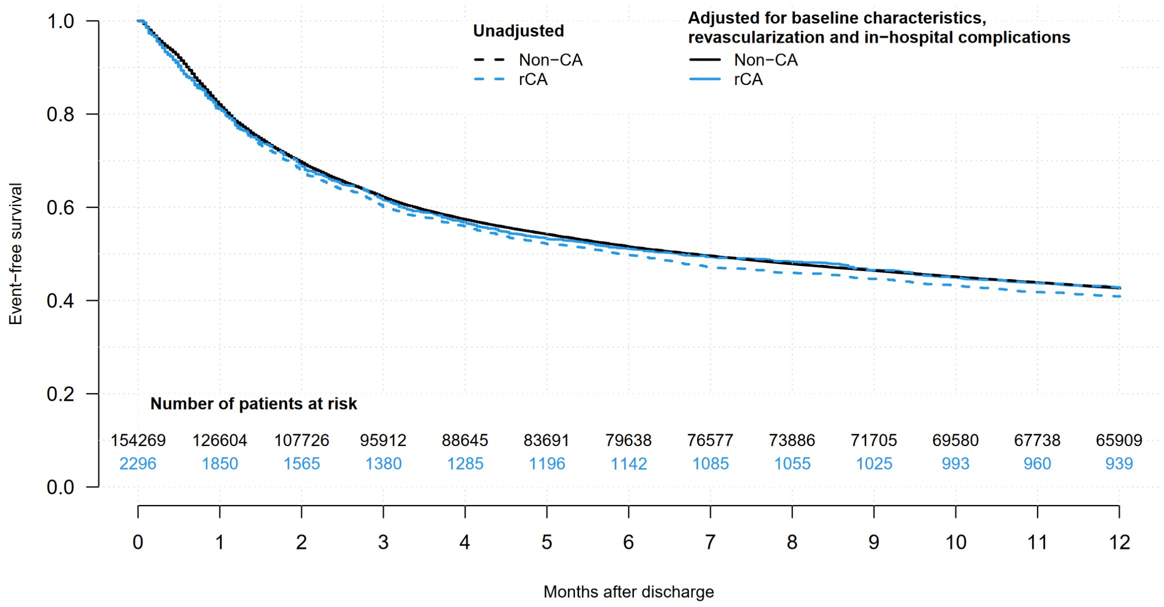

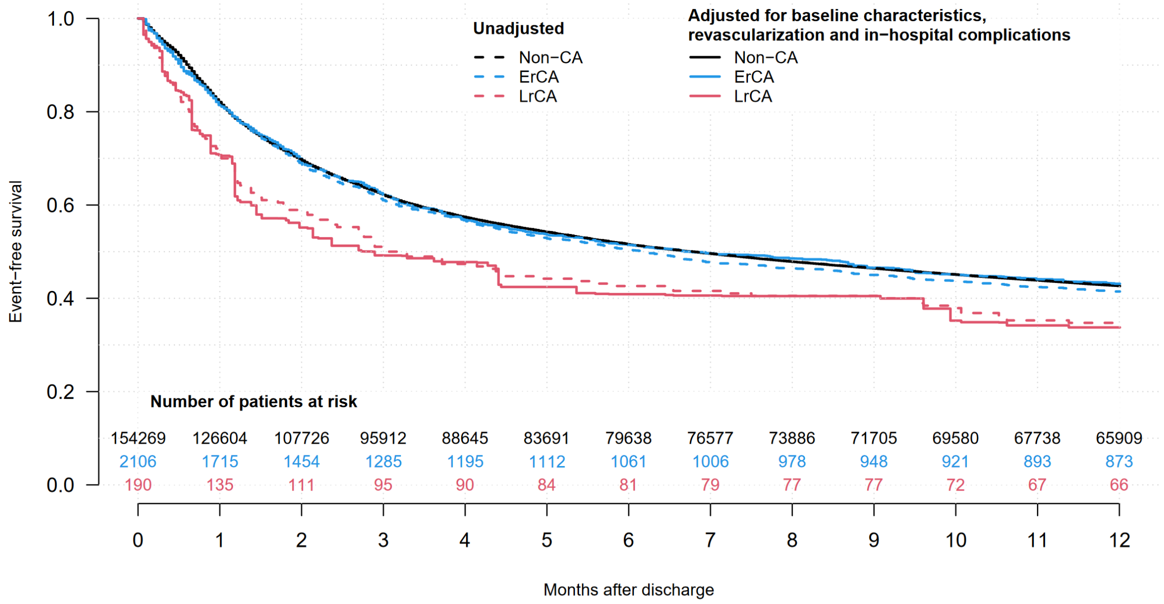

Adjusted for baseline characteristics, revascularization, in–hospital complications, and treatment procedures

	1-year event-free (95% CI)	Hazard ratio (95% CI)	P value
**Non-CA**	0.426 (0.424 - 0.429)	1	
**rCA**	0.423 (0.395 - 0.454)	1.02 (0.94 – 1.11)	0.624
**ErCA**	0.882 (0.860 - 0.905)	1.01 (0.93 – 1.10)	0.8297
**LrCA**	0.828 (0.751 – 0.913)	1.42 (1.05 – 1.90)	0.0217

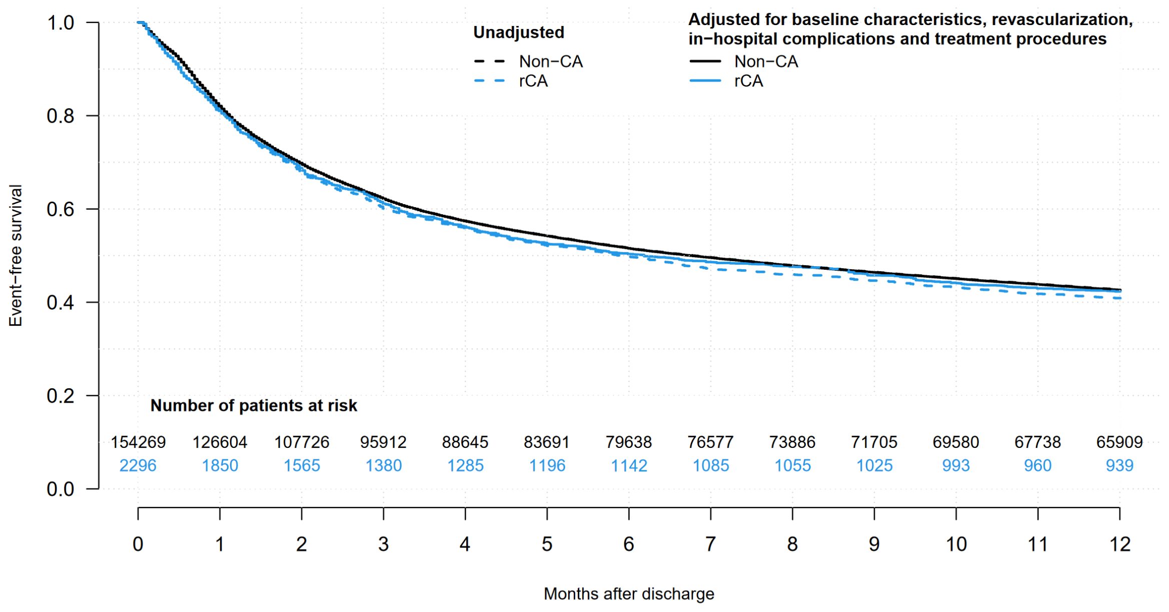

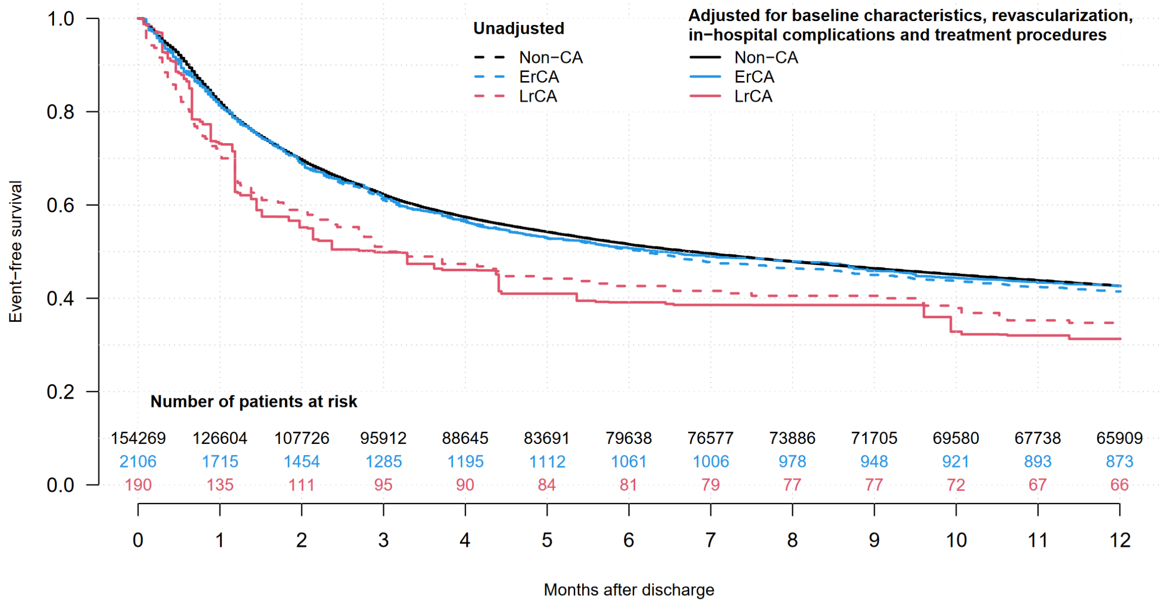

Relative risk of 1-year re-hospitalization due to any reason

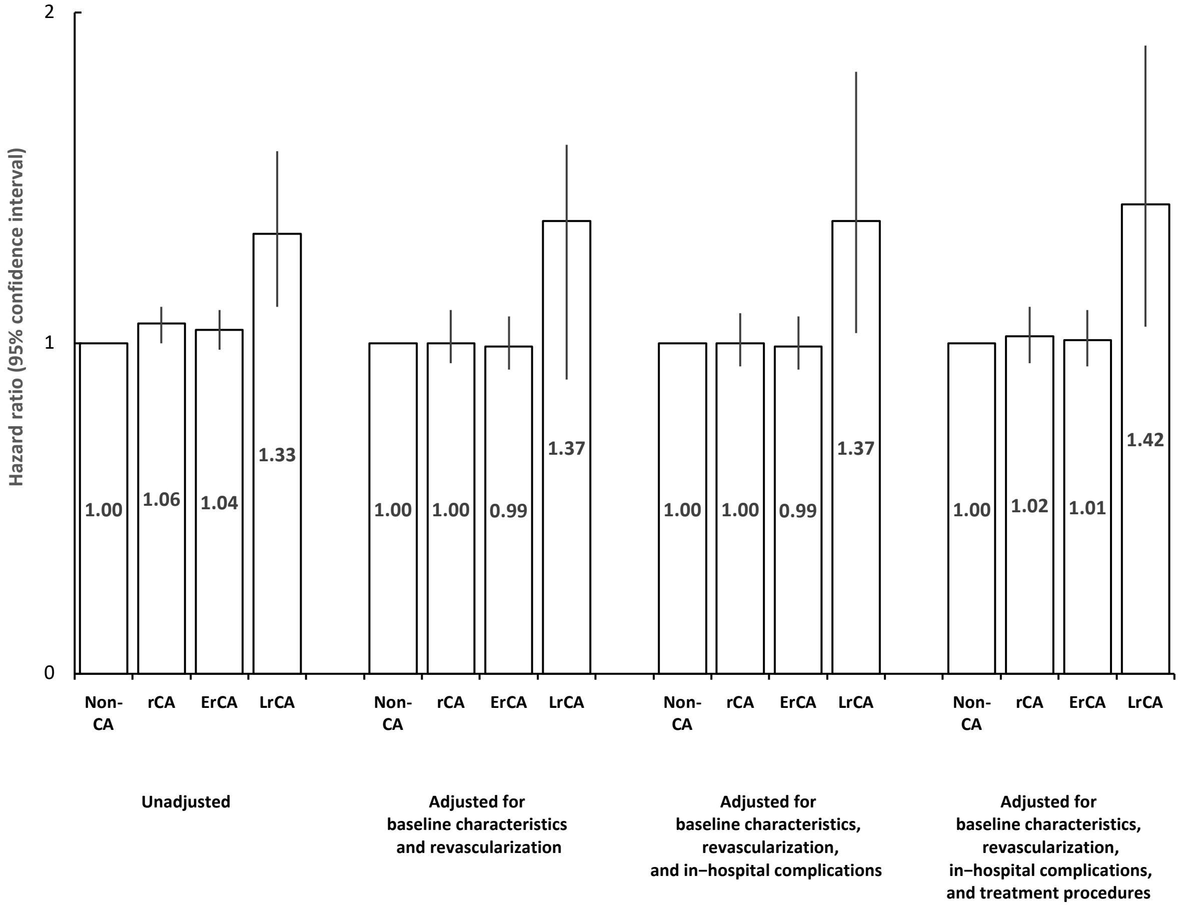

CA – cardiac arrest; rCA – resuscitated cardiac arrest; ErCA – early resuscitated cardiac arrest; LrCA – late resuscitated cardiac arrest

I) 1 year re-hospitalization with coronary angiography

Unadjusted

	1-year event-free (95% CI)	Hazard ratio (95% CI)	P value
**Non-CA**	0.743 (0.740 - 0.745)	1	
**rCA**	0.756 (0.739 - 0.774)	0.94 (0.86 – 1.02)	0.121
**ErCA**	0.749 (0.731 - 0.768)	0.97 (0.89 – 1.06)	0.47093
**LrCA**	0.837 (0.786 – 0.891)	0.59 (0.42 – 0.84)	0.00371

CI – confidence interval; CA – cardiac arrest; rCA – resuscitated cardiac arrest; ErCA – early resuscitated cardiac arrest; LrCA – late resuscitated cardiac arrest

Adjusted for baseline characteristics and revascularization

	1-year event-free (95% CI)	Hazard ratio (95% CI)	P value
**Non-CA**	0.743 (0.741 - 0.745)	1	
**rCA**	0.774 (0.751 - 0.797)	0.87 (0.77 – 0.97)	0.0152
**ErCA**	0.768 (0.743 - 0.793)	0.89 (0.79 – 1.01)	0.0663
**LrCA**	0.818 (0.743 – 0.901)	0.68 (0.42 – 1.10)	0.1162

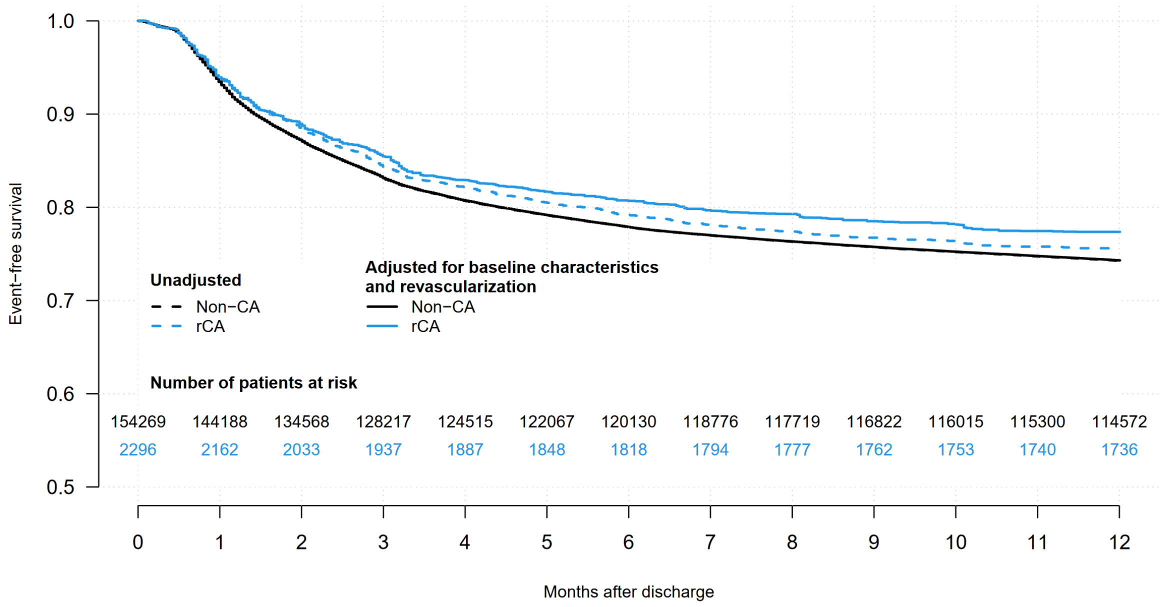

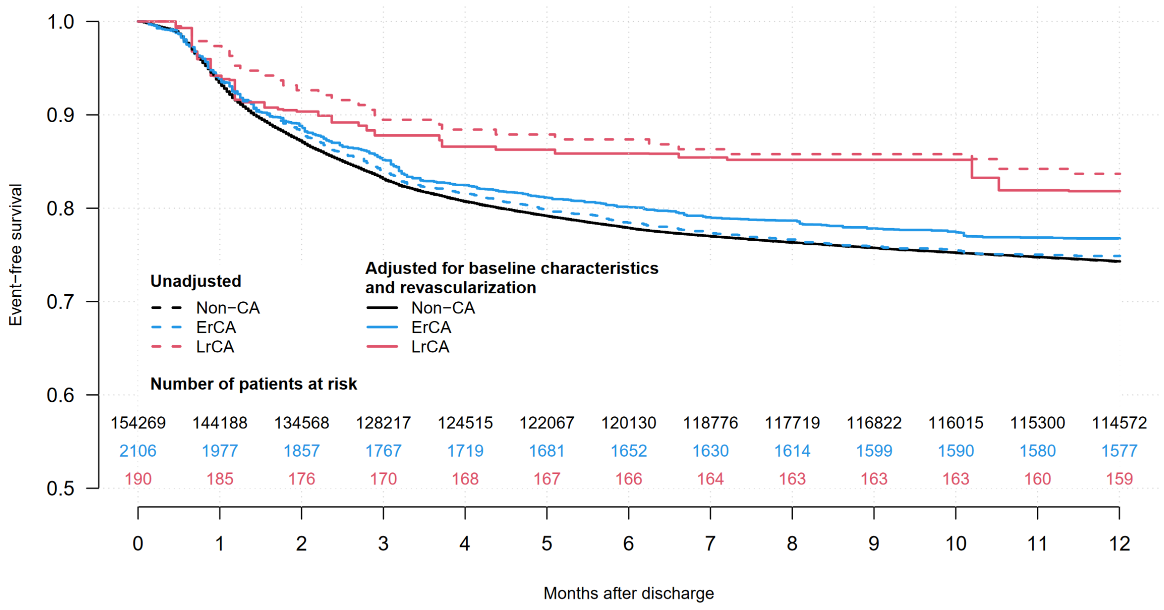

Adjusted for baseline characteristics, revascularization, and in−hospital complications

	1-year event-free (95% CI)	Hazard ratio (95% CI)	P value
**Non-CA**	0.743 (0.741 - 0.745)	1	
**rCA**	0.770 (0.747 - 0.795)	0.88 (0.78 – 0.99)	0.0391
**ErCA**	0.764 (0.739 - 0.790)	0.91 (0.80 – 1.03)	0.136
**LrCA**	0.832 (0.744 – 0.930)	0.62 (0.34 – 1.15)	0.128

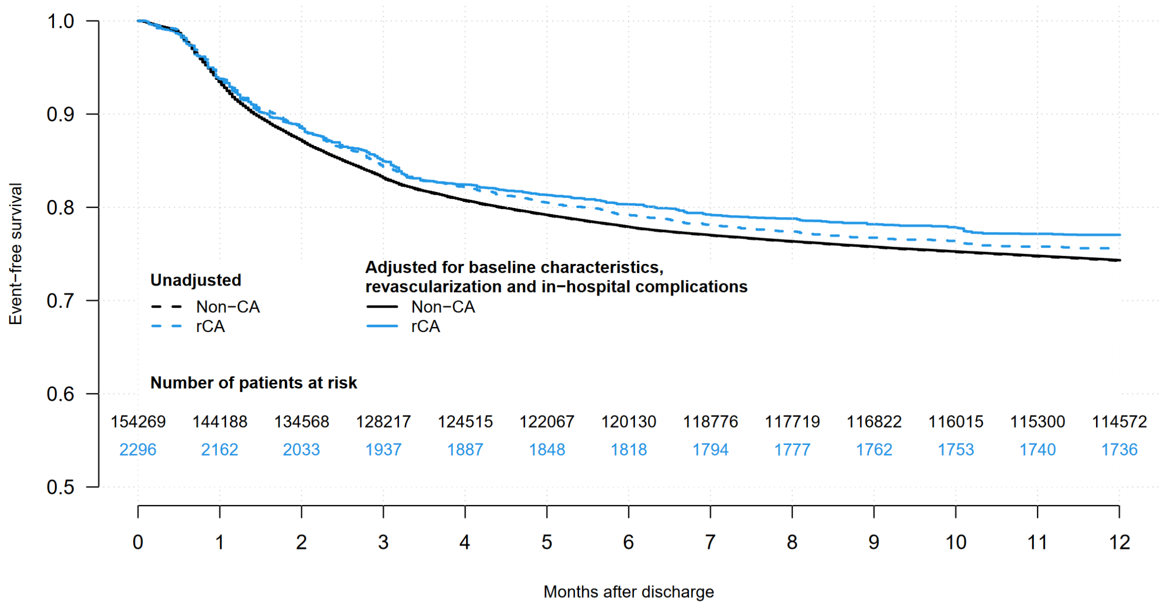

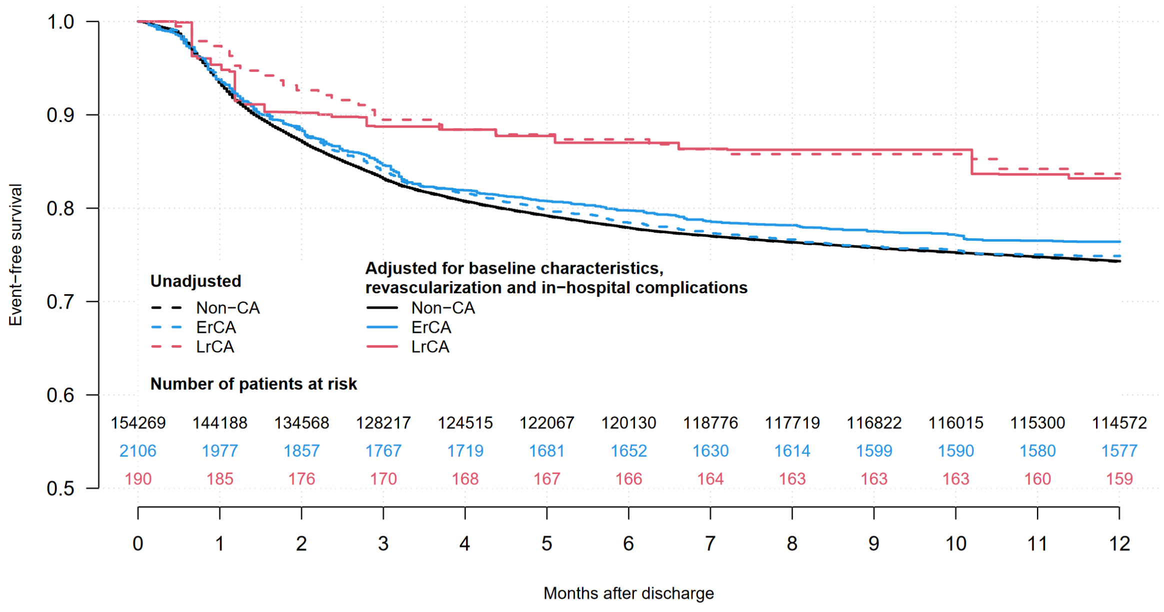

Adjusted for baseline characteristics, revascularization, in–hospital complications, and treatment procedures

	1-year event-free (95% CI)	Hazard ratio (95% CI)	P value
**Non-CA**	0.743 (0.741 - 0.746)	1	
**rCA**	0.765 (0.741 - 0.790)	0.91 (0.80 – 1.02)	0.107
**ErCA**	0.759 (0.734 - 0.786)	0.93 (0.82 – 1.06)	0.269
**LrCA**	0.826 (0.729 – 0.936)	0.65 (0.34 – 1.24)	0.190

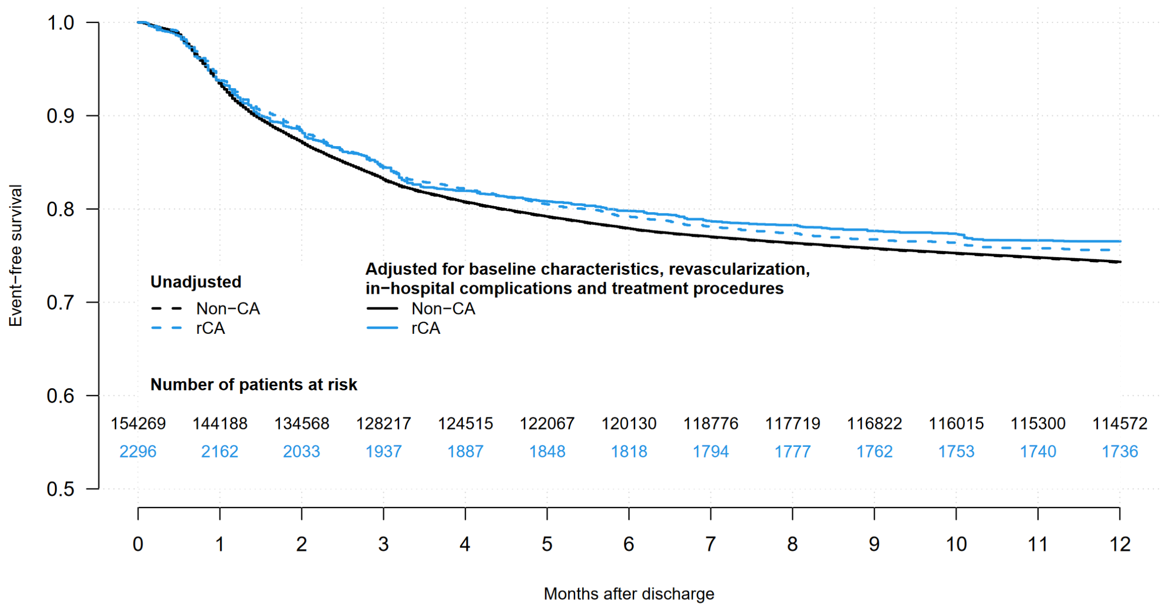

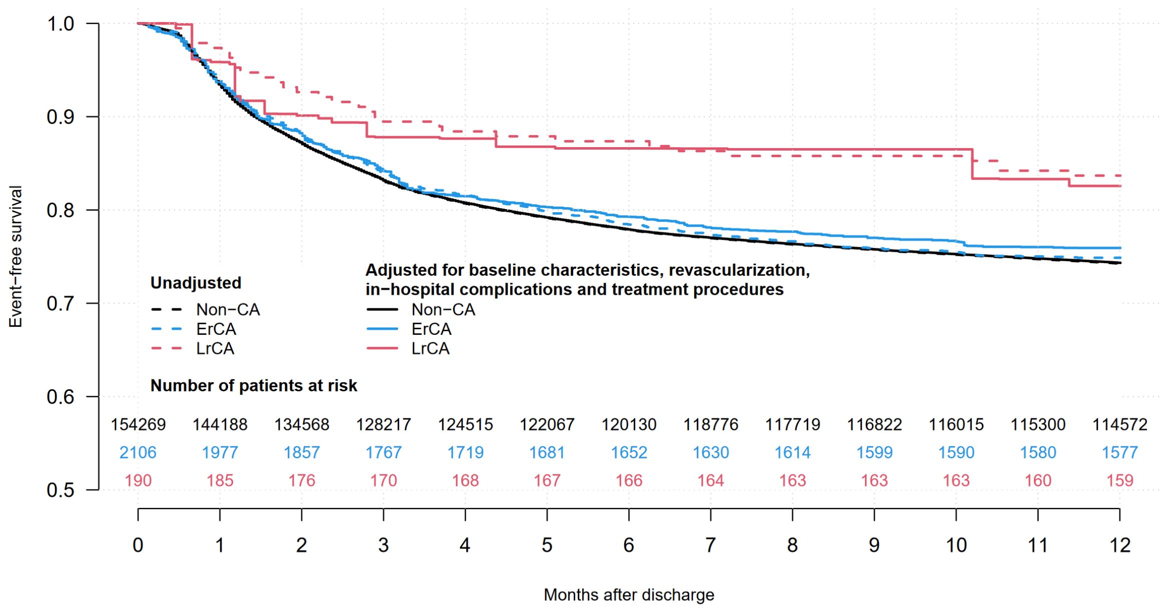

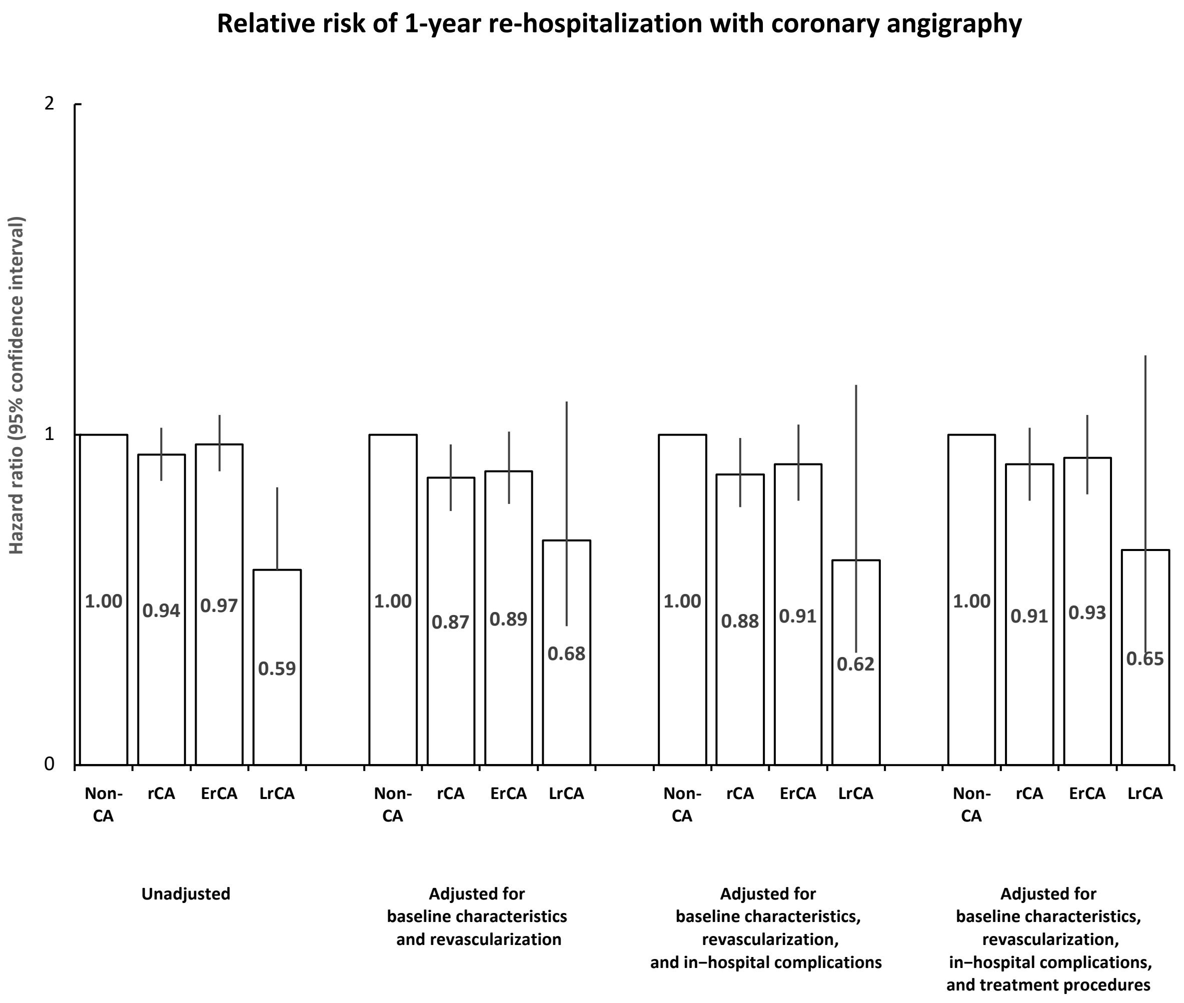

CA – cardiac arrest; rCA – resuscitated cardiac arrest; ErCA – early resuscitated cardiac arrest; LrCA – late resuscitated cardiac arrest

J) 1 year re-hospitalization with PCI or CABG

Unadjusted

	1-year event-free (95% CI)	Hazard ratio (95% CI)	P value
**Non-CA**	0.751 (0.749 - 0.753)	1	
**rCA**	0.790 (0.774 - 0.807)	0.82 (0.75 – 0.90)	<0.0001
**ErCA**	0.782 (0.765 - 0.800)	0.86 (0.78 – 0.94)	0.00101
**LrCA**	0.879 (0.834 – 0.927)	0.45 (0.30– 0.67)	0.00012

CI – confidence interval; CA – cardiac arrest; rCA – resuscitated cardiac arrest; ErCA – early resuscitated cardiac arrest; LrCA – late resuscitated cardiac arrest

Adjusted for baseline characteristics and revascularization

	1-year event-free (95% CI)	Hazard ratio (95% CI)	P value
**Non-CA**	0.751 (0.749 - 0.754)	1	
**rCA**	0.789 (0.766 - 0.812)	0.83 (0.74– 0.94)	0.0041
**ErCA**	0.783 (0.759 - 0.808)	0.86 (0.75 – 0.98)	0.0193
**LrCA**	0.839 (0.764 – 0.920)	0.62 (0.36 – 1.05)	0.0748

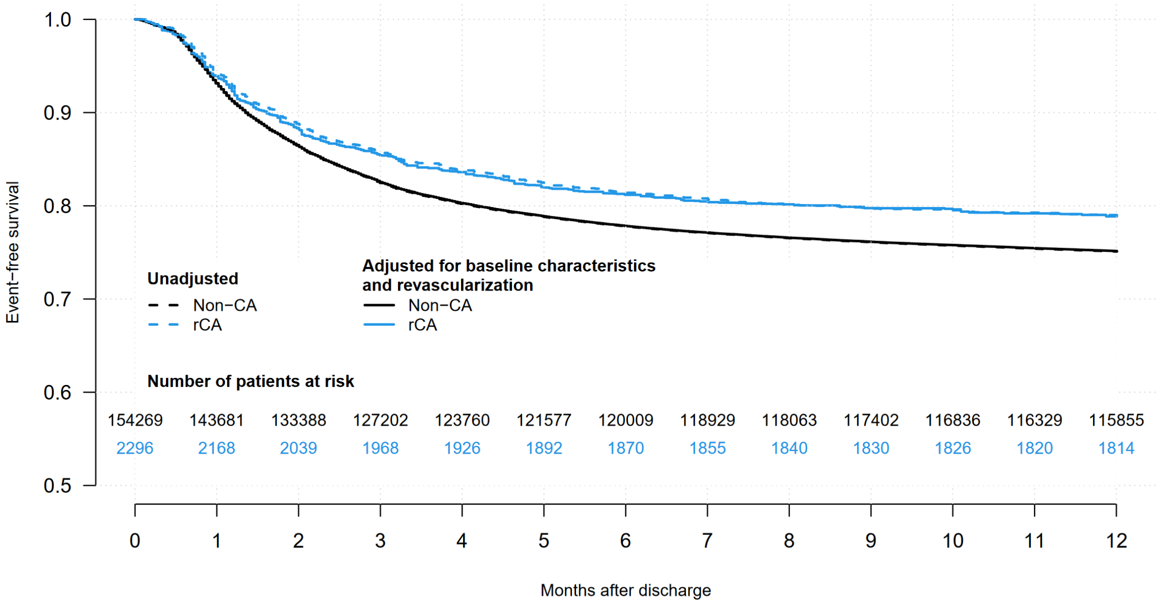

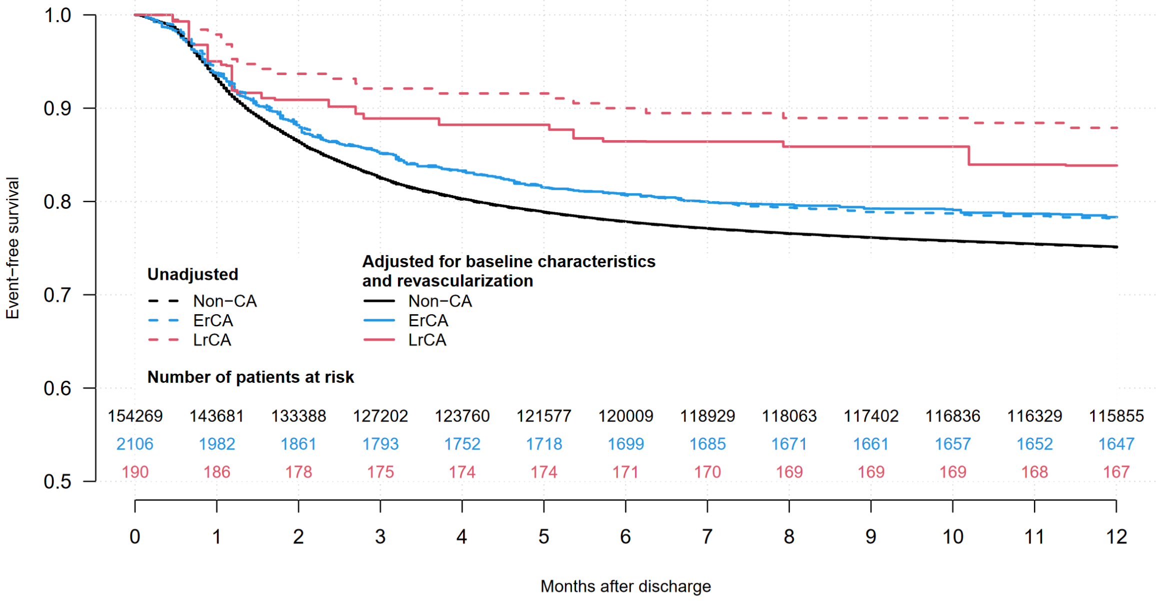

Adjusted for baseline characteristics, revascularization, and in−hospital complications

	1-year event-free (95% CI)	Hazard ratio (95% CI)	P value
**Non-CA**	0.752 (0.749 - 0.754)	1	
**rCA**	0.784 (0.761 - 0.808)	0.86 (0.75 – 0.97)	0.0159
**ErCA**	0.782 (0.757 - 0.808)	0.86 (0.76– 0.99)	0.0307
**LrCA**	0.821 (0.730 – 0.922)	0.69 (0.38– 1.25)	0.2213

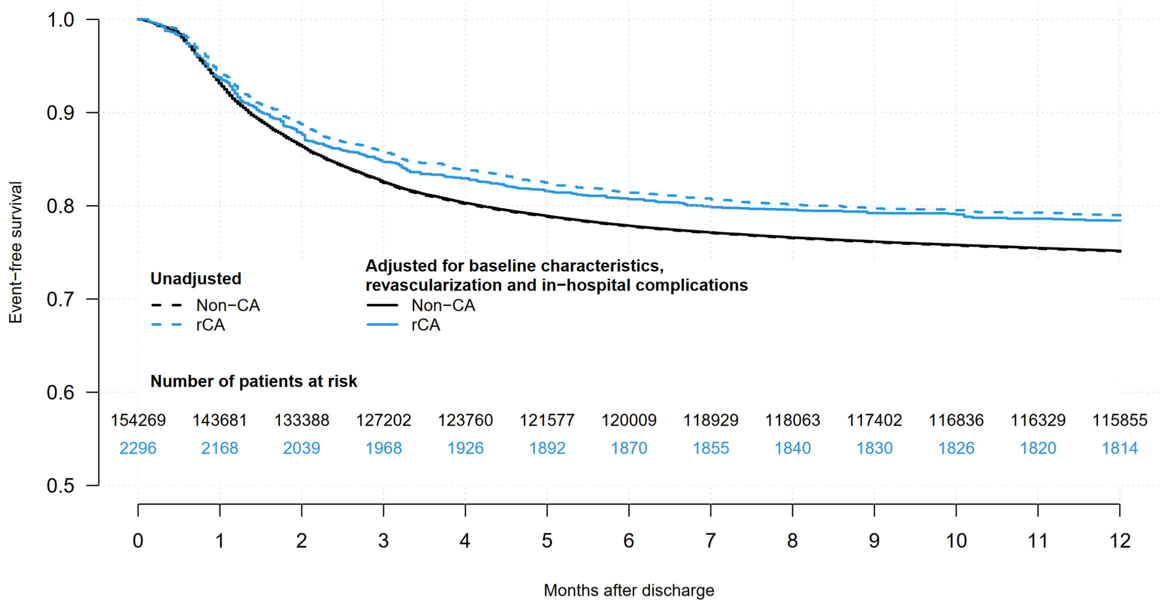

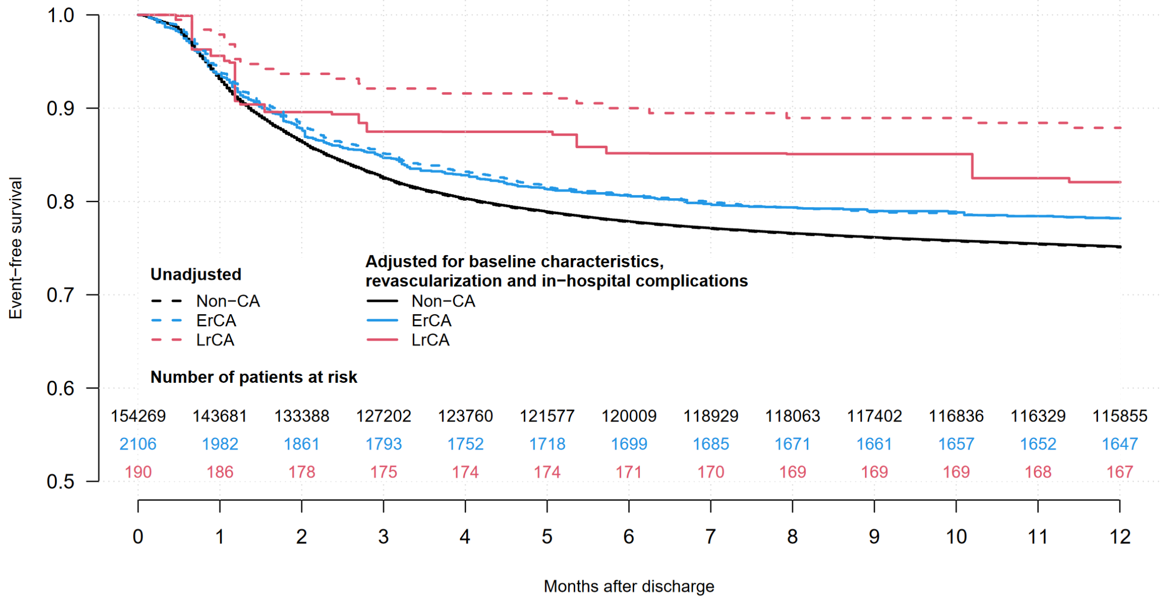

Adjusted for baseline characteristics, revascularization, in–hospital complications, and treatment procedures

	1-year event-free (95% CI)	Hazard ratio (95% CI)	P value
**Non-CA**	0.752 (0.750 – 0.754)	1	
**rCA**	0.779 (0.755 - 0.804)	0.88 (0.77 – 1.00)	0.0492
**ErCA**	0.778 (0.752 - 0.804)	0.89 (0.78 – 1.01)	0.0743
**LrCA**	0.803 (0.702 – 0.918)	0.76 (0.42 – 1.40)	0.3805

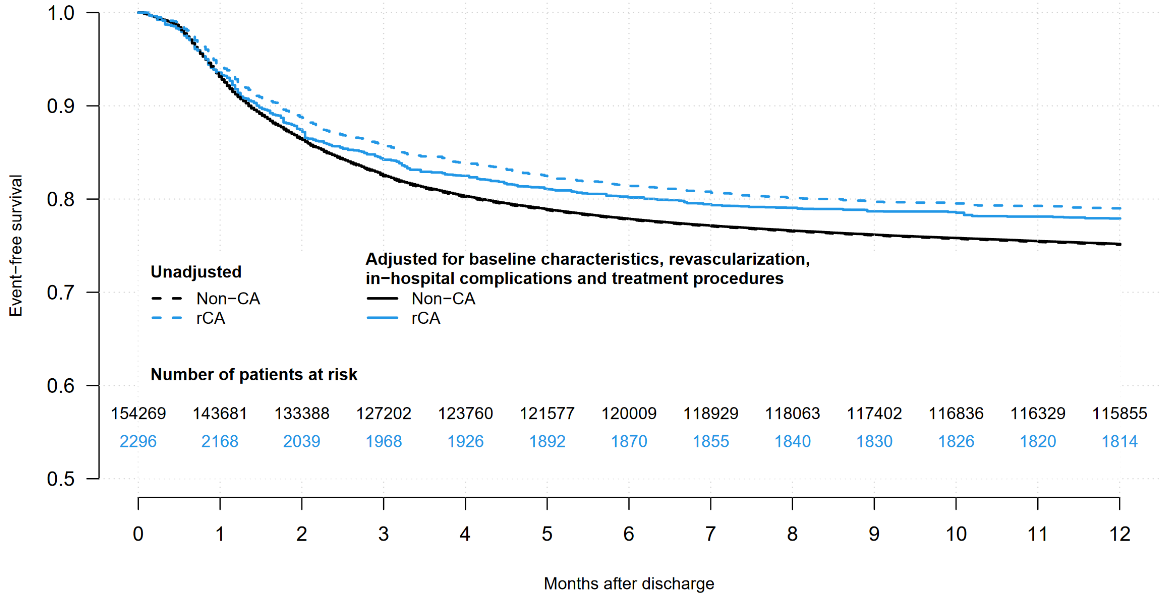

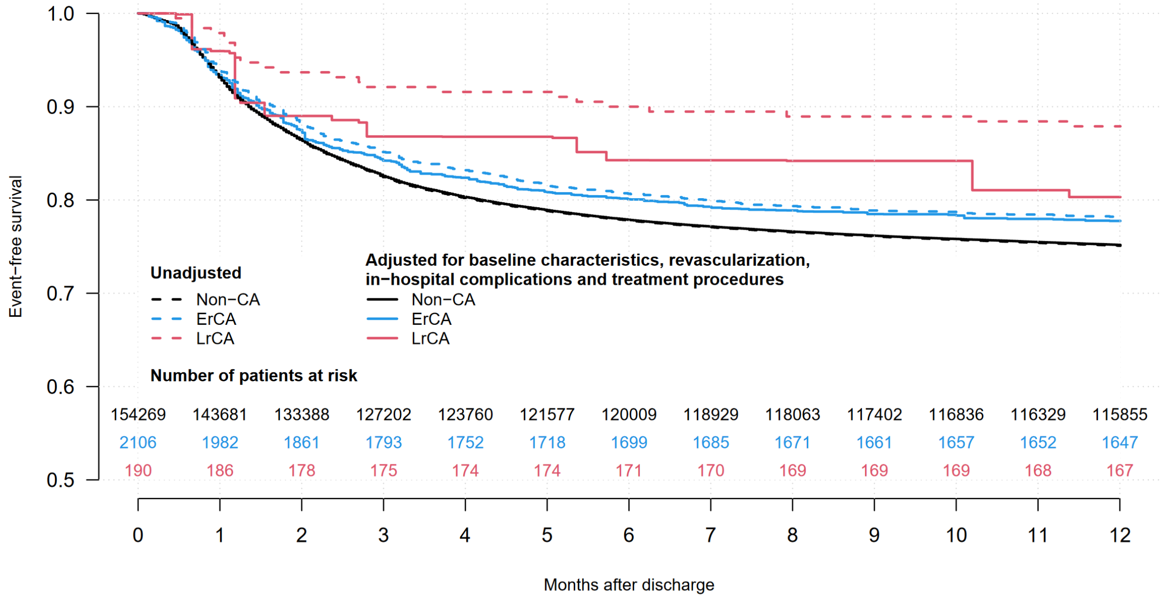

Relative risk of 1-year re-hospitalization with PCI and CABG

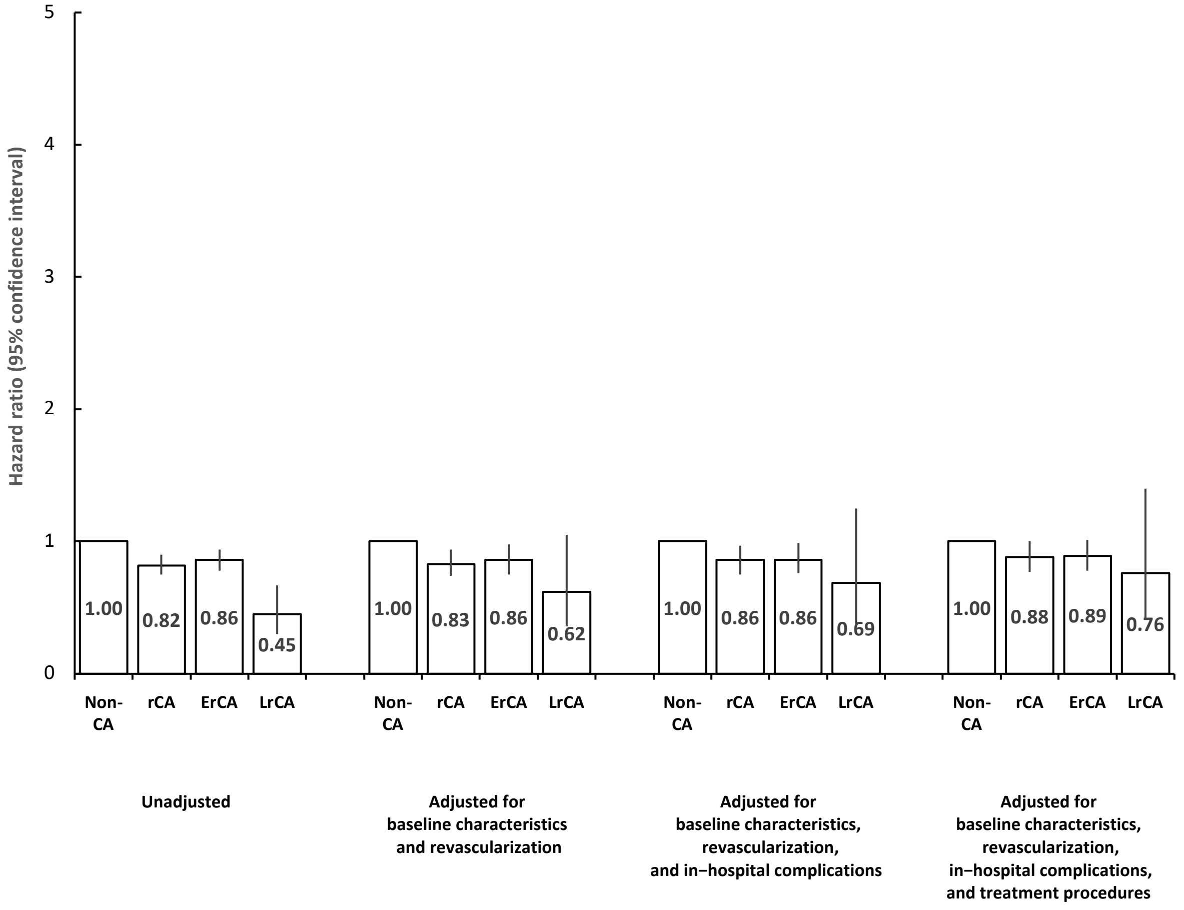

CA – cardiac arrest; rCA – resuscitated cardiac arrest; ErCA – early resuscitated cardiac arrest; LrCA – late resuscitated cardiac arrest

K) 1 year re-hospitalization with ICD or CRT-D implantation

Unadjusted

	1-year event-free (95% CI)	Hazard ratio (95% CI)	P value
**Non-CA**	0.983 (0.983 - 0.984)	1	
**rCA**	0.951 (0.942 - 0.960)	3.04 (2.52 – 3.67)	<0.0001
**ErCA**	0.952 (0.943 - 0.961)	2.96 (2.42 – 3.61)	<0.0001
**LrCA**	0.937 (0.903 – 0.972)	3.98 (2.26 – 7.02)	<0.0001

CI – confidence interval; CA – cardiac arrest; rCA – resuscitated cardiac arrest; ErCA – early resuscitated cardiac arrest; LrCA – late resuscitated cardiac arrest

Adjusted for baseline characteristics and revascularization

	1-year event-free (95% CI)	Hazard ratio (95% CI)	P value
**Non-CA**	0.983 (0.983 - 0.984)	1	
**rCA**	0.967 (0.958 - 0.975)	2.01 (1.54 – 2.63)	<0.0001
**ErCA**	0.967 (0.957 - 0.976)	2.01 (1.51 – 2.67)	<0.0001
**LrCA**	0.933 (0.872 – 0.998)	4.15 (1.57 – 11.05)	0.00445

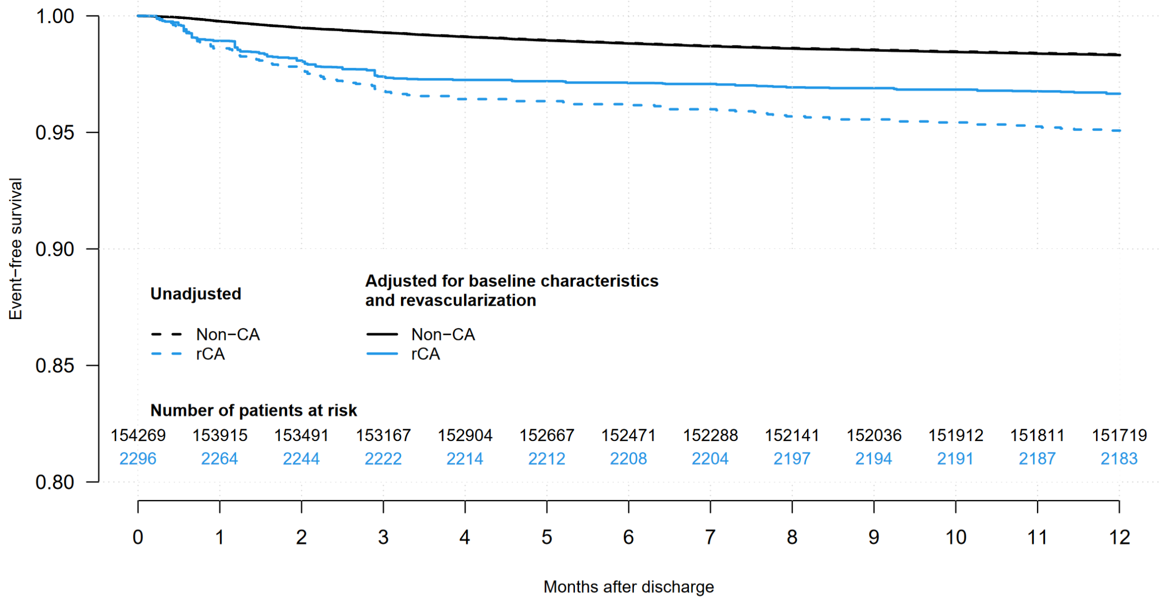

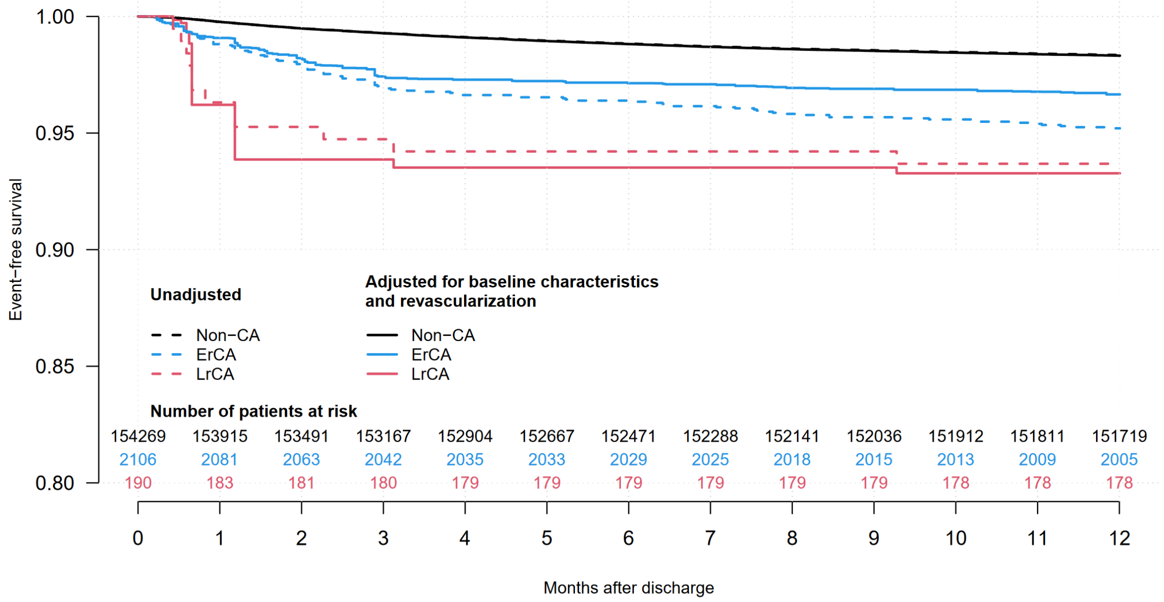

Adjusted for baseline characteristics, revascularization, and in–hospital complications

	1-year event-free (95% CI)	Hazard ratio (95% CI)	P value
**Non-CA**	0.983 (0.982 - 0.984)	1	
**rCA**	0.965 (0.955 - 0.975)	2.09 (1.57 – 2.79)	<0.0001
**ErCA**	0.965 (0.954 - 0.975)	2.10 (1.55 – 2.84)	<0.0001
**LrCA**	0.927 (0.861 – 0.997)	4.50 (2.22 – 11.86)	0.00243

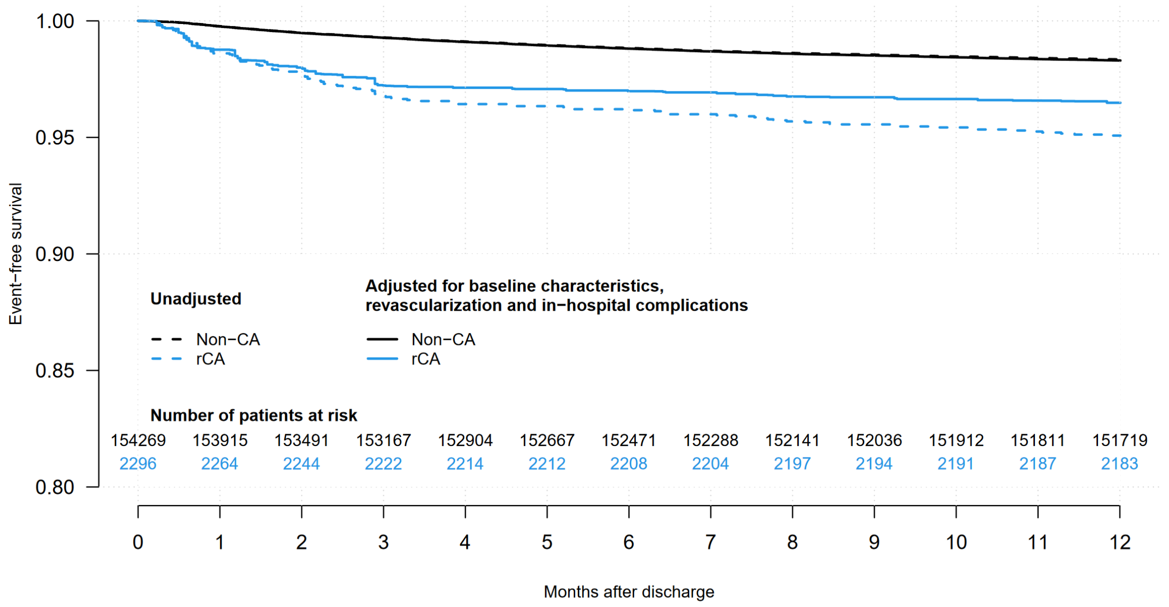

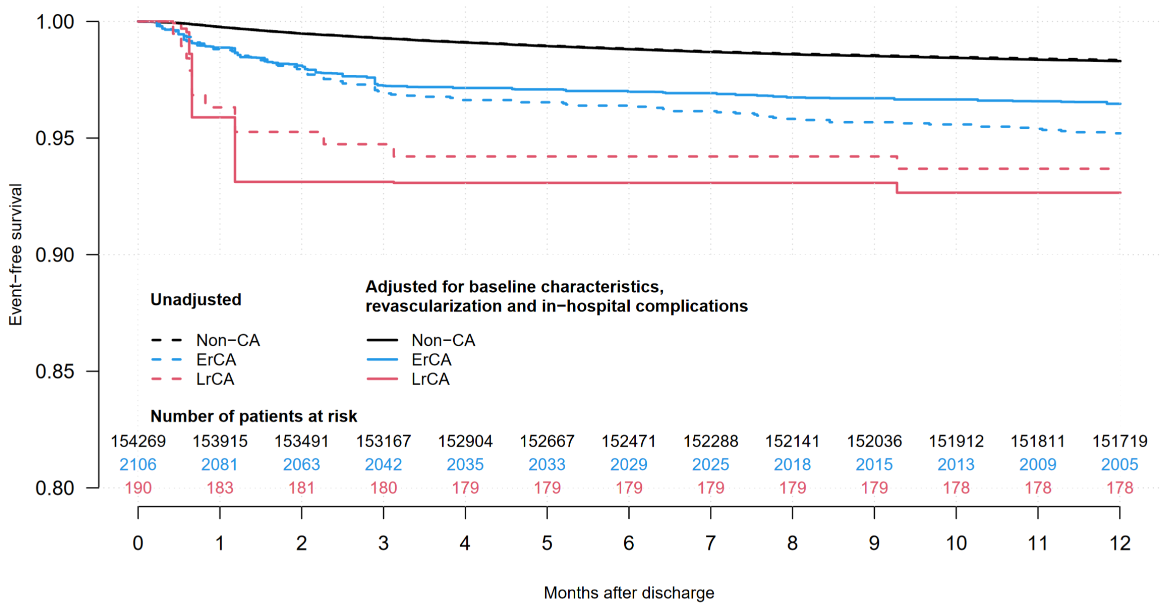

Adjusted for baseline characteristics, revascularization, in–hospital complications, and treatment procedures

	1-year event-free (95% CI)	Hazard ratio (95% CI)	P value
**Non-CA**	0.983 (0.982 – 0.984)	1	
**rCA**	0.962 (0.951 - 0.972)	2.28 (1.71 – 3.04)	<0.0001
**ErCA**	0.962 (0.951 - 0.974)	2.23 (1.65 – 3.03)	<0.0001
**LrCA**	0.911 (0.836 – 0.992)	5.50 (2.19 – 13.83)	0.000295

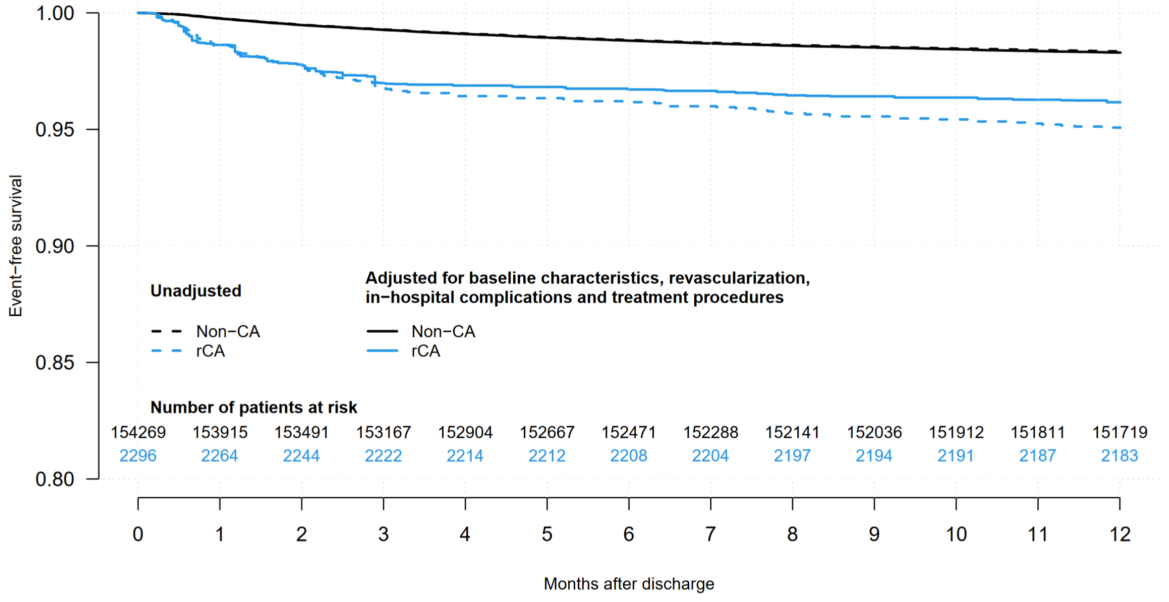

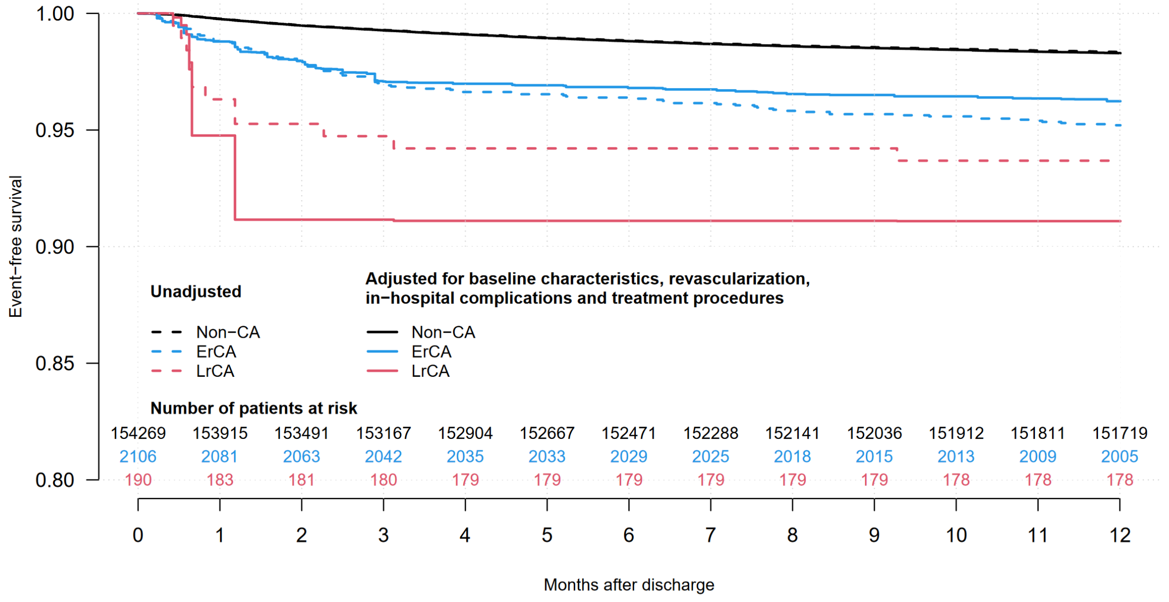

Relative risk of 1-year re-hospitalization with ICD or CRT-D implantation

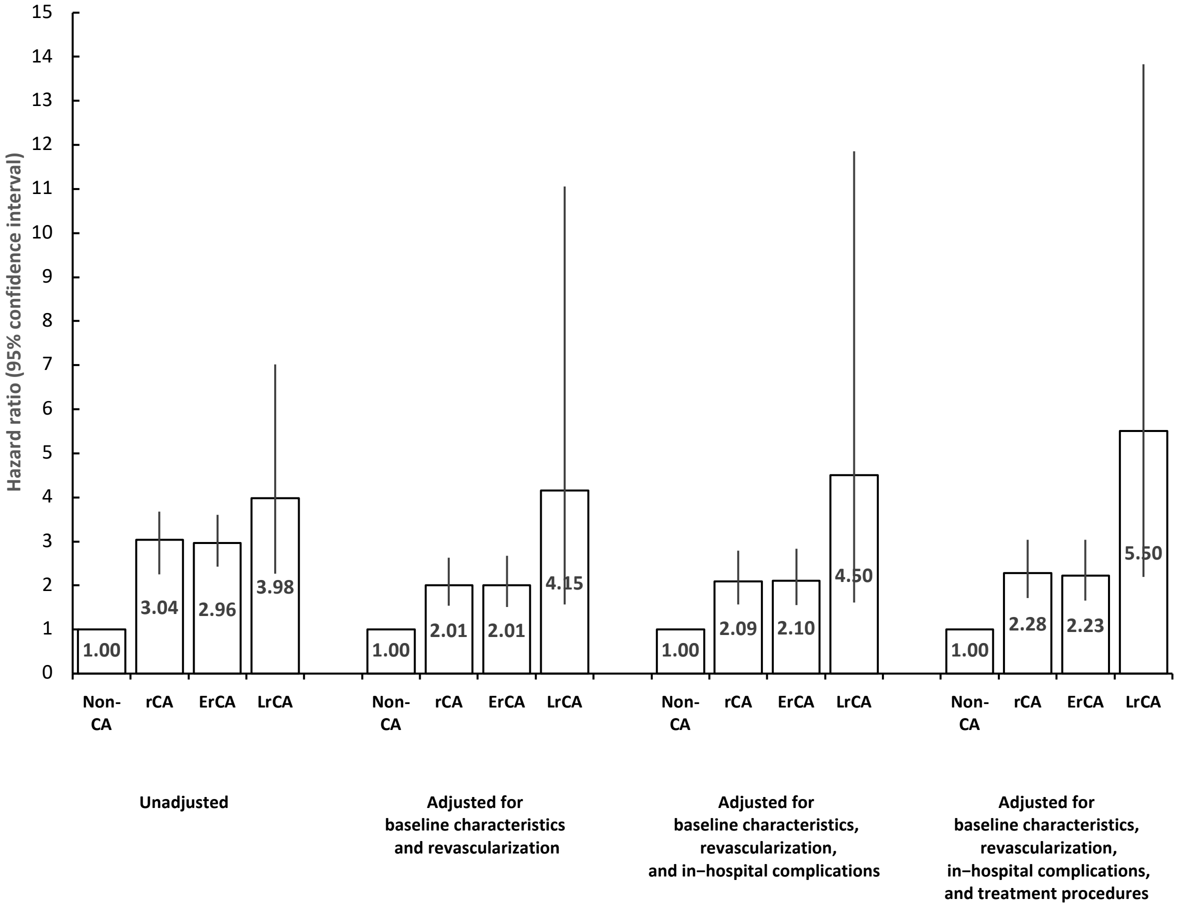

CA – cardiac arrest; rCA – resuscitated cardiac arrest; ErCA – early resuscitated cardiac arrest; LrCA – late resuscitated cardiac arrest
